# Heterotelechelic Silicones: Facile Synthesis and Functionalization Using Silane-Based Initiators

**DOI:** 10.1021/acs.macromol.3c01802

**Published:** 2023-10-29

**Authors:** Yoichi Okayama, Taejun Eom, Michael Czuczola, Allison Abdilla, Jacob R. Blankenship, Kaitlin R. Albanese, Javier Read de Alaniz, Christopher M. Bates, Craig J. Hawker

**Affiliations:** † Materials Research Laboratory, University of California, Santa Barbara, California 93106, United States; ‡ Department of Chemistry & Biochemistry, University of California, Santa Barbara, California 93106, United States; § Materials Department, University of California, Santa Barbara, California 93106, United States; ∥ Department of Chemical Engineering, University of California, Santa Barbara, California 93106, United States

## Abstract

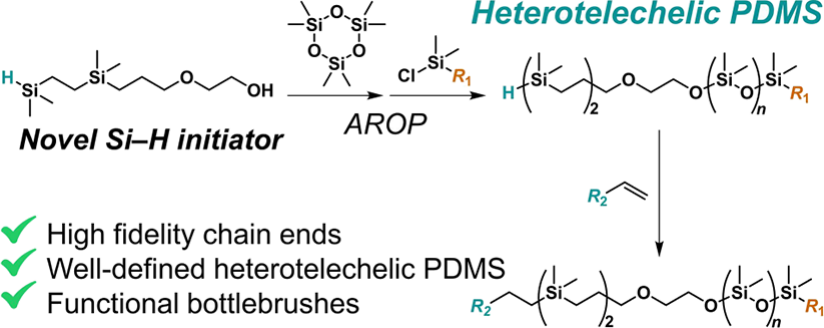

The synthetic utility of heterotelechelic polydimethylsiloxane (PDMS) derivatives is limited due to challenges in preparing materials with high chain-end fidelity. In this study, anionic ring-opening polymerization (AROP) of hexamethylcyclotrisiloxane (D_3_) monomers using a specifically designed silyl hydride (Si–H)-based initiator provides a versatile approach toward a library of heterotelechelic PDMS polymers. A novel initiator, where the Si–H terminal group is connected to a C atom (H–Si–C) and not an O atom (H–Si–O) as in traditional systems, suppresses intermolecular transfer of the Si–H group, leading to heterotelechelic PDMS derivatives with a high degree of control over chain ends. In situ termination of the D_3_ propagating chain end with commercially available chlorosilanes (alkyl chlorides, methacrylates, and norbornenes) yields an array of chain-end-functionalized PDMS derivatives. This diversity can be further increased by hydrosilylation with functionalized alkenes (alcohols, esters, and epoxides) to generate a library of heterotelechelic PDMS polymers. Due to the living nature of ring-opening polymerization and efficient initiation, narrow-dispersity (*Đ* < 1.2) polymers spanning a wide range of molar masses (2–11 kg mol^–1^) were synthesized. With facile access to α-Si–H and ω-norbornene functionalized PDMS macromonomers (H–PDMS–Nb), the synthesis of well-defined supersoft (*G*′ = 30 kPa) PDMS bottlebrush networks, which are difficult to prepare using established strategies, was demonstrated.

## Introduction

Polydimethylsiloxane (PDMS) is the most common siloxane polymer due to excellent optical, electrical, and mechanical properties including low glass-transition temperature (*T*_g_), low surface energy, and high gas permeability.^1−4^ Based on these properties, PDMS is used in a broad range of applications such as coatings,^3,5^ photolithography,^5−11^ microfluidics,^12−14^ electronics,^15−19^ and biomedicine.^20−22^ For these and many other applications, simple derivatives with identical chain ends are employed which is in direct contrast to vinyl and anionic ring-opening systems, such as poly(ethylene oxide), where the synthesis of polymers with heterotelechelic chain ends is readily achieved and their orthogonal reactivity enhances the performance of imaging agents,^23^ targeting ligands,^24,25^ nanoparticles,^26−28^ engineered surfaces, and proteins.^29−31^ To similarly translate PDMS to additional high-value applications, a robust and user-friendly synthesis of heterotelechelic PDMS derivatives with different functional groups at each chain end is required.

Synthetic methods for PDMS can be classified into three polymerization types: polycondensation, thermodynamically controlled ring-opening polymerization, and kinetically controlled anionic ring-opening polymerization (AROP).^32−34^ Among them, AROP is the most common polymerization method to access polymers with high chain-end fidelity, high molecular weight, and low molar mass dispersity. Examples of conventional AROP for PDMS include the polymerization of hexamethylcyclotrisiloxane (D_3_) initiated with an alkyl-lithium reagent, such as *n*-butyl lithium, followed by termination with a functionalized chlorosilane,^34−38^ to give monofunctionalized (ω-functionalized) PDMS derivatives.

**Figure 1:**
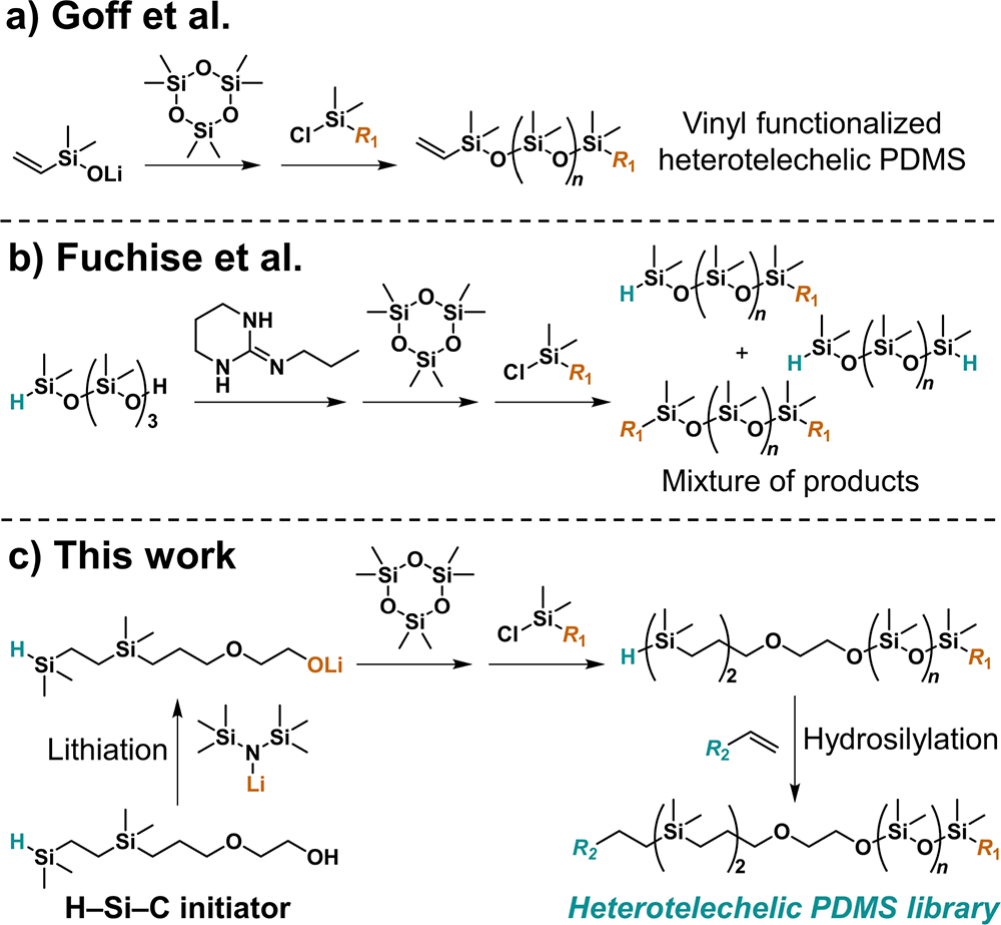
Comparison of synthetic approaches to heterotelechelic PDMS derivatives.

While a wide range of low-dispersity, ω-monofunctionalized PDMS derivatives are commercially available, access to functional heterotelechelic PDMS systems is limited. In 2016, Goff et al. reported the AROP of D_3_ using lithium vinyldimethylsilanolate as an initiator (Figure 1a).^39^ After termination with dimethylchlorosilane, α-vinyl-ω-silyl hydride (Si–H) PDMS with good chain-end fidelity was obtained, although the coreactivity of vinyl and Si–H groups precluded secondary functionalization without chain–chain coupling. In 2020, Fuchise et al. reported the AROP of D_3_ initiated from functionalized silanols and catalyzed by a strong organic base leading to a mixture of hetero- and homofunctionalized PDMS derivatives.^40,41^ In this system, the synthesis and purification of different silanol initiators are required to control the α-chain-end functional group. Notably, initiation with Si–H containing silanols results in an undesired mixture of telechelic byproducts as well as the desired heterotelechelic derivative. This mixture was attributed to intermolecular transfer of the Si–H group between propagating chains. Based on these challenges, the allure of preparing a library of heterotelechelic PDMS derivatives with a single Si–H chain end is driven by the versatility of hydrosilylation chemistry. Using a variety of catalysts, hydrosilylation is widely employed in industry and proceeds under mild conditions with high selectivity and yield.^42−44^ Furthermore, the Si–H moiety can undergo other quantitative reactions including carbonyl hydrosilylation,^45−49^ Piers–Rubinsztajn chemistry,^50−52^ and oxidative coupling.^2,53^

To enable new opportunities with PDMS-based materials, a novel synthetic route is reported to access a wide range of heterotelechelic PDMS derivatives via a scalable one-pot AROP process. Key to the success of this strategy is the development of an initiator containing an H–Si–C motif that suppresses chain scrambling (Figure 1), which replaces the more labile Si–O bond found in the aforementioned traditional systems. As a result, a single Si–H α-chain-end is maintained throughout the polymerization, which can be further functionalized via hydrosilylation. This orthogonality also allows a wide variety of ω-chain-ends to be easily incorporated by simple termination of the propagating siloxy anion with a range of commercially available functionalized chlorosilanes. To showcase the high chain-end fidelity and utility of these materials, we also demonstrate a simple and scalable method for preparing well-defined PDMS bottlebrush networks. Access to these PDMS-based super-soft materials has otherwise been limited by the synthetic availability of ω-functionalized PDMS.

## Results and Discussion

**Scheme 1:**
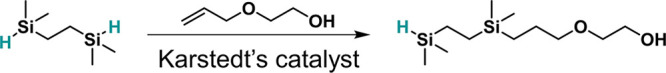
Synthesis of H–Si–C Initiator via Hydrosilylation

A key feature of this strategy is the design of a novel Si–H-functionalized initiator. In order to prevent the formation of telechelic byproducts, we hypothesized that replacing the labile H–Si–O terminal unit with a H–Si–C motif would significantly reduce nucleophilic cleavage and intermolecular chain transfer due to the increased stability of the Si–C bonds. The synthesis of this new initiator was achieved by hydrosilylation of 1,2-bis(dimethylsilyl)ethane with ethylene glycol allyl ether catalyzed by Karstedt’s catalyst (10 ppm in toluene) (Scheme 1). Following purification, the H–Si–C initiator was obtained as a colorless oil in 52% yield, with purity and long-term stability confirmed by ^1^H-, ^13^C-, and ^29^Si-nuclear magnetic resonance (NMR) spectroscopy (Figures S1–S3).

**Figure 2:**
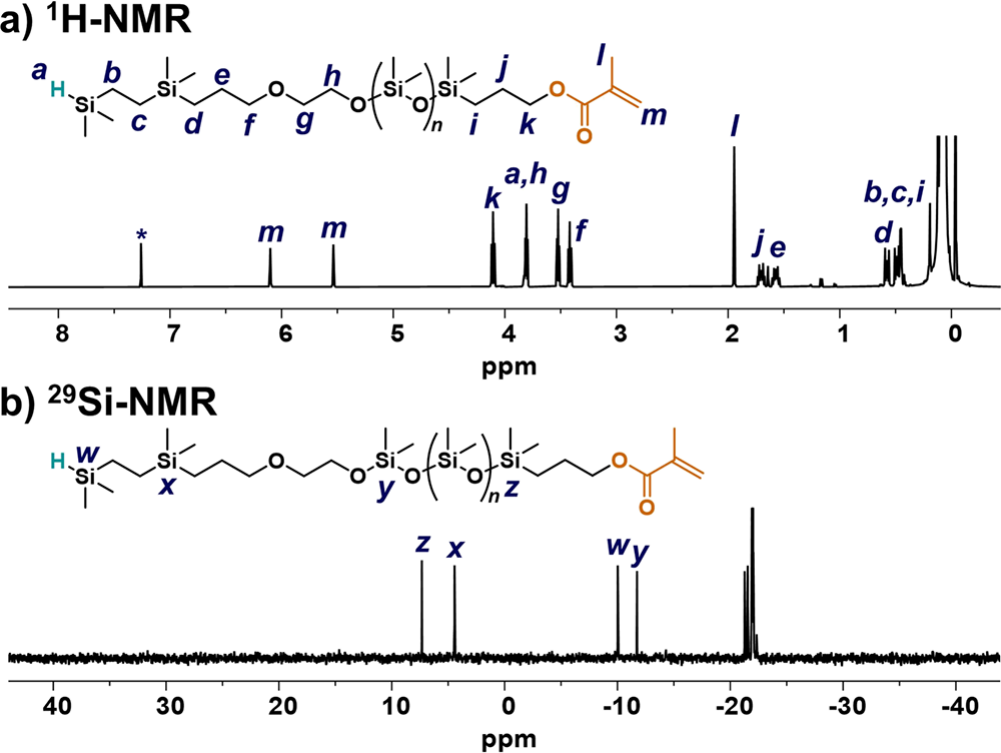
(a) ^1^H NMR spectrum of H–PDMS–MA with unique resonances for the α-Si–H and ω-methacrylate chain ends labeled. (b) ^29^Si-NMR spectrum of H–PDMS–MA shows four distinct Si resonances well-separated from the PDMS backbone (−23 to −20 ppm) that are attributed to the well-defined chain ends.

Anionic ring-opening polymerization of D_3_ was then investigated by initial lithiation of the H–Si–C initiator with lithiumhexamethyldisilazane (LiHMDS) in anhydrous hexanes. After the mixture was stirred for 10 min at room temperature, D_3_ monomer was added followed by DMF as a promoter. While AROP of D_3_ does not proceed in nonpolar solvents such as hexanes, polymerization does occur once a polar cosolvent, commonly referred to as a promoter, is added.^35^ After 15 min, the polymerization was quenched at 60% conversion (calculated from ^1^H-NMR) by adding 3-methacryloxypropyldimethylchlorosilane to obtain the desired heterotelechelic PDMS derivative with a 1:1 ratio of α-Si–H and ω-methacrylate PDMS (H–PDMS–MA) chain ends as confirmed by integration of unique resonances in the ^1^H-NMR spectrum (Figure 2a, peaks “a” and “m”). In addition, ^29^Si-NMR revealed resonances fully consistent with both unique chain ends and a high degree of chain-end fidelity (Figure 2b). The retention of the reactive and orthogonal Si–H chain end was further confirmed by Fourier transform infrared (FT-IR) spectroscopy, where a strong absorbance at 2100 cm^–1^ is observed (Figure S7).

**Figure 3:**
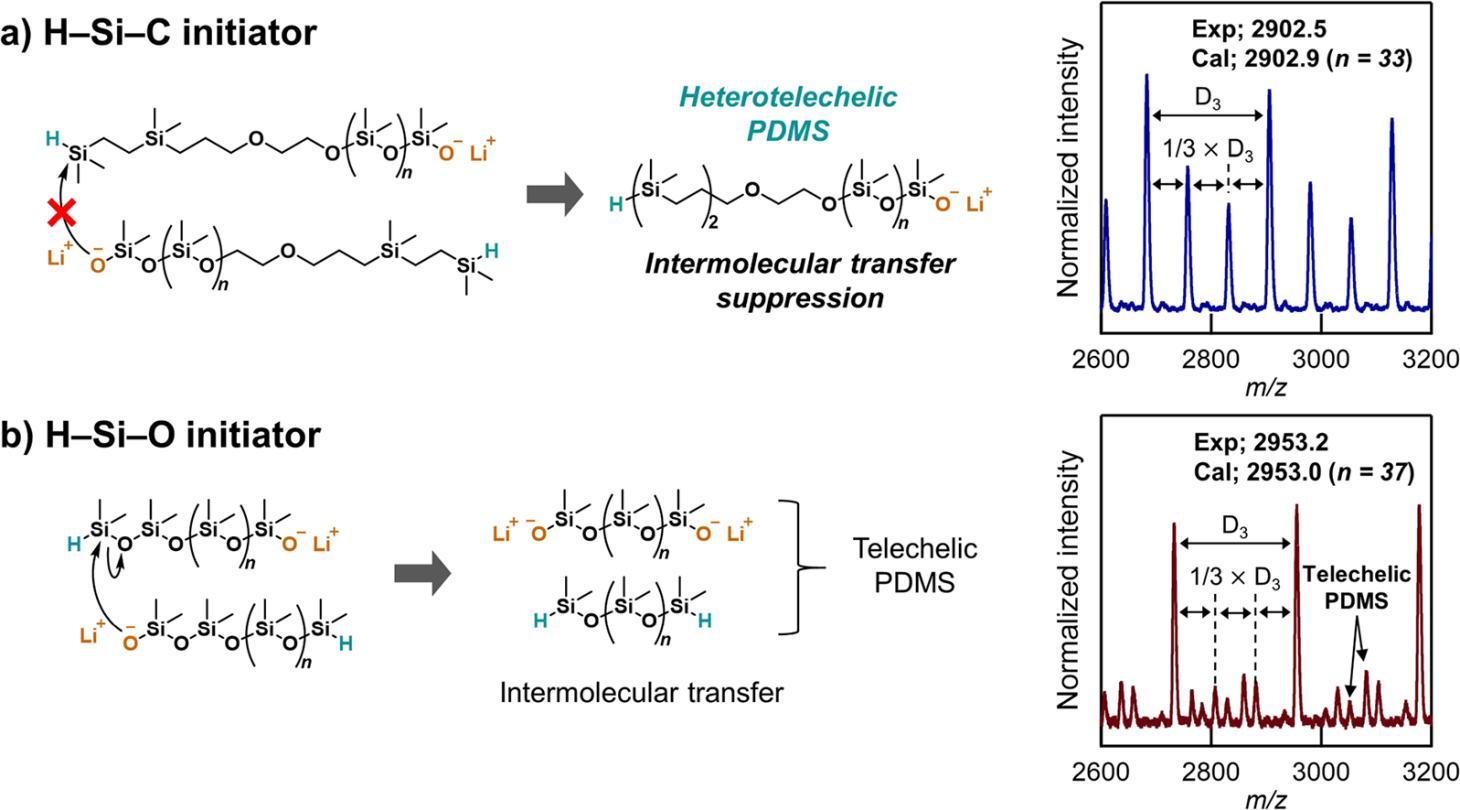
MALDI mass spectrum of H–PDMS–MA synthesized from either the (a) H–Si–C or (b) H–Si–O initiator. Intermolecular transfer of the dimethylsilyl group is suppressed through the use of the H–Si–C initiator, illustrating improved stability.

A significant reduction in intermolecular transfer was verified by matrix-assisted laser desorption/ionization (MALDI) mass spectrometry, where only one set of periodic peaks was observed for the heterotelechelic derivative (Figure 3a). Further analysis in each case reveals a mass difference of 74 Da between peaks that correspond with the dimethylsiloxane repeat unit (74 Da). In addition, the observed *m*/*z* values for each peak correlate with the calculated molar mass of H–PDMS–MA (plus associated sodium cation). In direct contrast, AROP of D_3_ using 1-hydroxy-1,1,3,3,5,5,7,7-tetrasiloxane (H–(SiOMe_2_)_4_–OH) as the initiator (containing an H–Si–O unit) under the same polymerization conditions yields multiple sets of peaks with *m*/*z* values that correspond to a mixture of telechelic H–PDMS–H and MA–PDMS–MA chains as well as the desired heterotelechelic H–PDMS–MA product (Figures 3b and S10–S16). These results clearly illustrate the presence of byproducts caused by intermolecular transfer and the critical role played by the stable H–Si–C unit of the initiator. The stability of this linkage also allows heterotelechelic polymers with high chain-end fidelity to be obtained by suppressing the intermolecular transfer of the Si–H group.

**Figure 4:**
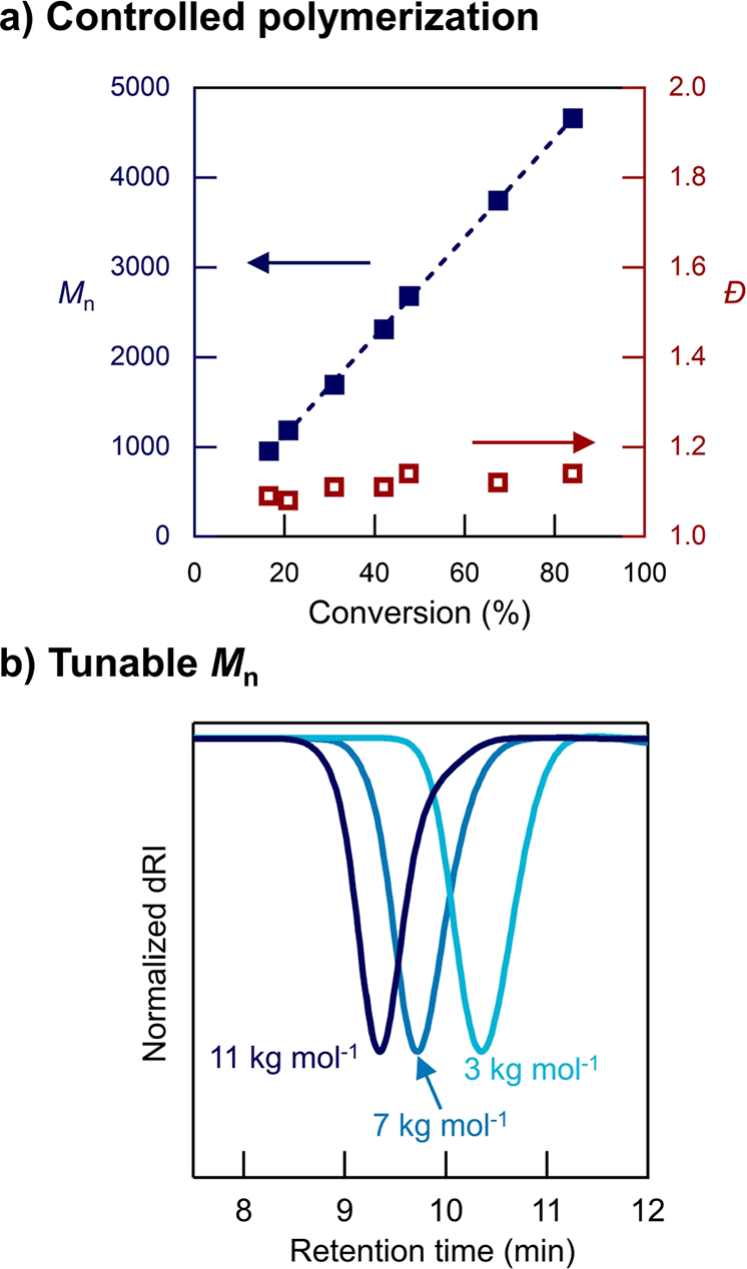
(a) Plot of *M*_n_ and *Đ* with conversion for the AROP of D_3_ using the H–Si–C initiator. (b) SEC trace in chloroform of heterotelechelic H–PDMS–MA derivatives polymerized with different M/I ratios (20, 45, and 90, corresponding with 3, 7, and 11 kg mol^–1^, respectively). Narrow distributions are maintained even for high molecular weight polymers (e.g.*, M*_n_ = 11 kg mol^–1^).

For the AROP of D_3_ using the novel H–Si–C initiator, the living character was then investigated by following the evolution of the molecular weight *M*_n_ with both time and conversion. As shown in Figure 4, quenching the polymerization at different conversions leads to a linear increase in molecular weight while retaining low dispersity (*Đ* < 1.2), even at high conversions (>80%) (Figure 4a). The living nature of this polymerization system also allowed the molar mass of heterotelechelic PDMS derivatives to be readily controlled by varying the monomer/initiator (M/I) ratio. Figure 4b shows size-exclusion chromatography (SEC) chromatograms for different M/I ratios (20, 45, and 90) with all polymerizations quenched at 15 min, targeting 60% conversion. SEC analysis indicates a close correlation between the experimental and theoretical molecular weights for molar masses as high as 11 kg mol^–1^ with *Đ* < 1.2. Notably, this degree of control is difficult to achieve with other polymerization methods such as polycondensation or thermodynamically controlled ring-opening polymerization.^32,34,35^ Higher molar masses (*M*_n_ > 20 kg mol^–1^) can also be obtained, although with a larger degree of uncertainty when characterizing the heterotelechelic functionality due to increased difficulty in reliably quantifying polymer chain ends using MALDI or NMR techniques.

After establishing the controlled AROP of D_3_ monomer from the H–Si–C initiator, a library of heterotelechelic PDMS derivatives was prepared through a two-step process involving functional termination of the propagating silanoate chain end followed by orthogonal postpolymerization transformation of the Si–H chain end using hydrosilylation chemistry. To demonstrate the versatility of this process, a variety of functional groups were installed on the ω-chain-end by quenching the AROP with commercially available vinyl-, chloropropyl (Cl), or norbornene (Nb)-functionalized chlorosilanes. These derivatives—fully characterized by ^1^H-NMR, ^13^C-NMR, ^29^Si-NMR, SEC, MALDI, and FT-IR—illustrate the stability of the Si–H chain end during polymerization and functional termination (Figures S17–S34). From this initial library, the α-Si–H chain end could be selectively functionalized postpolymerization under mild conditions using Karstedt’s catalyst with a range of terminal alkenes. Hydrosilylation of H–PDMS–MA with hydroxy, epoxy, and trimethoxy silyl [(MeO)_3_Si]-containing alkenes was quantitative and resulted in telechelic PDMS derivatives with a single HO–, epoxy–, and (MeO)_3_Si–PDMS–MA chain end (Figures 5 and S35–S52). Of particular note, a common initiator for atom-transfer radical polymerization (ATRP) based on a tertiary α-bromo carbonyl group was readily coupled with H–PDMS–Cl (Figures S53–S58) to give the ATRP-PDMS-Cl macroinitiator (*M*_n_ = 3.1 kg mol^–1^, *Đ* < 1.2) which allows for facile access to PS-*b*-PDMS–Cl block copolymers via ATRP with styrene (*M*_n_ = 5.5 kg mol^–1^, *Đ* < 1.1, Figures 6 and S59–S62). These results illustrate the general nature of this orthogonal strategy for preparing a range of heterotelechelic PDMS systems.

**Figure 5:**
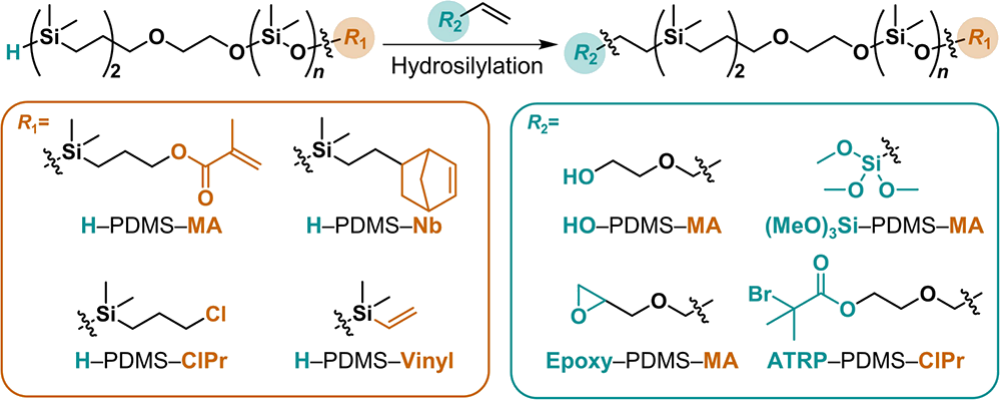
General scheme for the synthesis of a heterotelechelic PDMS library. Methacrylate (MA), chlorine (Cl), norbornene (Nb), and vinyl groups were introduced at the ω-chain-end by terminating the AROP with functional chlorosilanes. Introduction of hydroxy (HO), epoxy, trimethoxysilyl ((MeO)_3_Si), and α-bromo carbonyl (ATRP) at the α-chain-ends by hydrosilylation with terminal alkenes.

**Figure 6:**
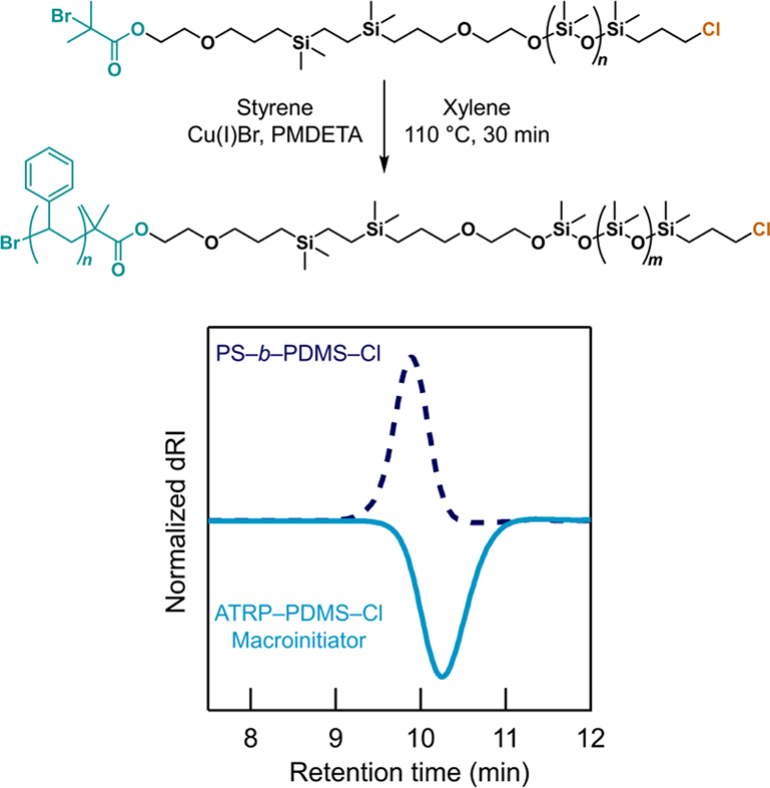
Synthesis of the PS–*b*-PDMS–Cl block copolymer via ATRP. SEC trace in chloroform shows a well-defined PS–*b*-PDMS–Cl block copolymer (*M*_n_ = 5.5 kg mol^–1^, *Đ* < 1.1) obtained by chain extension of ATRP–PDMS–Cl (*M*_n_ = 3.1 kg mol^–1^, *Đ* < 1.2) with styrene.

**Figure 7:**
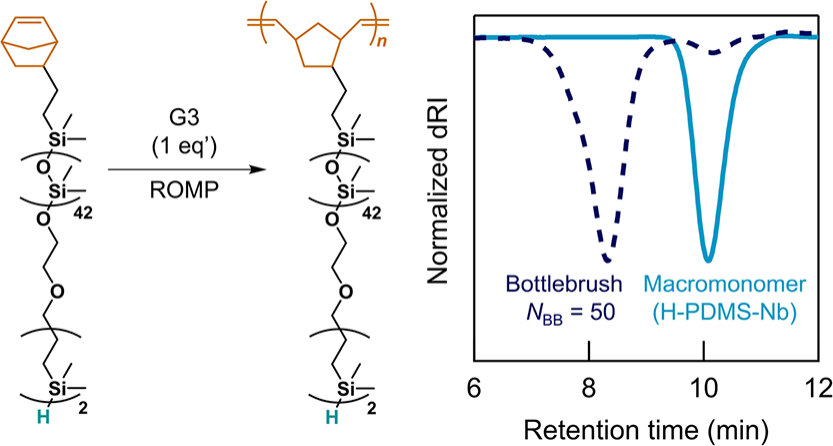
ROMP of the H–PDMS–Nb macromonomer to form a Si–H-functionalized bottlebrush polymer. SEC trace in chloroform of the crude sample illustrates high chain-end fidelity for the starting macromonomer.

To demonstrate the utility of this scalable strategy to heterotelechelic PDMS derivatives, a variety of PDMS bottlebrushes was prepared via grafting-through polymerization starting from heterotelechelic macromonomers. This leads to multifunctional bottlebrush polymers with a single Si–H end-group for each grafted arm—an otherwise challenging proposition with traditional strategies. Ring-opening metathesis polymerization (ROMP) was used to polymerize H–PDMS–Nb with Grubbs’ third-generation catalyst (G3) targeting a backbone degree of polymerization *N*_BB_ = 50 and 100. In both cases, ^1^H NMR spectroscopy confirmed quantitative conversion of the norbornene end-group with successful bottlebrush synthesis being demonstrated by SEC (*N*_BB_ = 50: *M*_n_ = 52 kg mol^–1^, *Đ* < 1.4; *N*_BB_ = 100: *M*_n_ = 128 kg mol^–1^, *Đ* < 1.6). Analysis of the crude samples showed minor amounts of residual macromonomer (∼1–2% by peak area), with no evidence of a high molecular weight shoulder that would indicate the presence of bifunctional Nb–PDMS–Nb impurities in the original macromonomer (Figures 7 and S63–S67). Significantly, full retention of the Si–H group after ROMP and bottlebrush formation was confirmed by ^1^H-NMR, ^29^Si-NMR, and FT-IR spectroscopy (Figures S63–S66).

**Figure 8:**
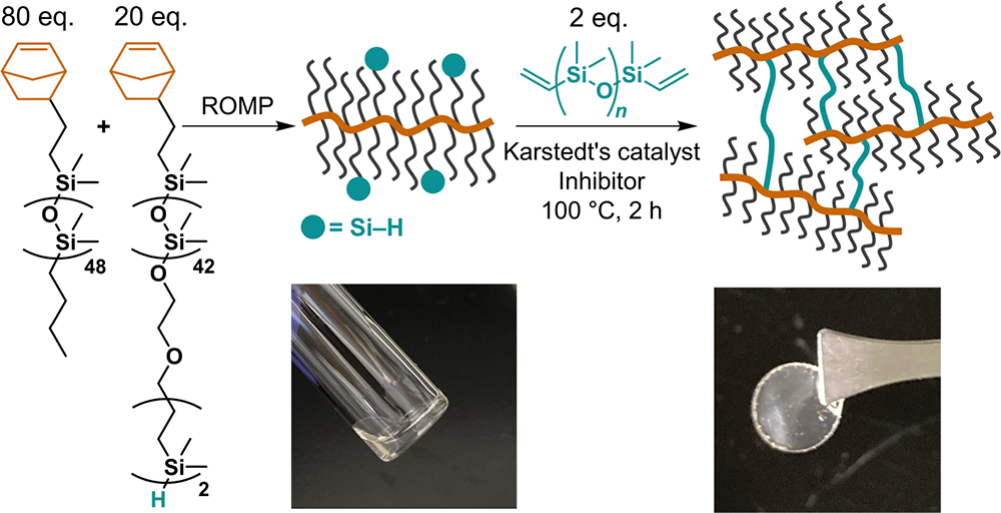
Synthesis of PDMS bottlebrush networks cross-linked through coupling of the divinyl cross-linker with the Si–H bonds of the bottlebrush copolymer. Photographs before and after heating.

The ability to successfully homopolymerize H–Si macromonomers further opens up the potential to modulate the level of chain-end functionality through copolymerization with monofunctional PDMS macromonomers. In turn, this versatility allows well-defined, solvent-free PDMS bottlebrush networks to be prepared via controlled cross-linking, for example, by copolymerizing H–PDMS–Nb with Bu–PDMS–Nb (*N*_BB_ = 100, *N*_SC,Bu-PDMS-Nb_ = 48, *N*_SC,H-PDMS-Nb_ = 42). For demonstration purposes, the ratio of H–PDMS–Nb to Bu–PDMS–Nb was 1:4 (Figures S68–S73) and the resulting bottlebrush copolymers (*N*_BB_ = 100, *M*_n_ = 129 kg mol^–1^, *Đ* < 1.6, Figures S74–S78) could be mixed with commercially available bis-vinyl PDMS cross-linker and dimethyl maleate (inhibitor) followed by the addition of Karstedt’s catalyst. Without dimethyl maleate (inhibitor), network formation occurred almost immediately on mixing at room temperature. In contrast, in the presence of an inhibitor, the mixture maintained a liquid-like state and underwent gelation at ∼4 h which allows the reaction mixture to be easily poured into a mold and cured at 100 °C for 2 h to give a fully cross-linked material (Figure 8). The stability of this formulation also allows in situ cross-linking kinetics to be measured at 100 °C in an oscillatory rheometer by monitoring the curing process. Curing was observed to proceed rapidly as the temperature approaches 100 °C and plateaus after 30 min as evidenced by measurement of the storage modulus (Figure S79). Following cross-linking, samples were cooled to 25 °C, and the rheological properties were examined. Frequency sweeps at room temperature reveal a low rubbery plateau modulus of 30 kPa (Figure S80) that can be attributed to the bottlebrush architecture.^54,55^ Significantly, these well-defined bottlebrush networks are considerably softer than Sylgard 184 (filled system) as well as comparable high molecular weight silicone elastomers (unfilled system) (∼1 and 0.6 MPa, respectively).^1,56,57^ The orthogonal nature of the hydrosilylation and ROMP chemistries also leaves open the possibility of installing other chain end functional groups in the network either before or after curing.

## Conclusions

In summary, a versatile synthetic approach was developed to access a range of heterotelechelic PDMS derivatives via living AROP. Key to the success of this robust synthetic strategy is the development and use of a new H–Si–C-functionalized initiator. The H–Si–C units play a critical role in preventing the intermolecular transfer of the chain end and allow a library of heterotelechelic PDMS materials to be prepared with accurate control over molecular weight while maintaining a low dispersity. Multiple functional groups can be introduced at the ω-chain-end by using a variety of commercially available chlorosilane terminators with the functional group tolerance of hydrosilylation allowing a range of α-chain-ends to be prepared via hydrosilylation. The modular nature of this two-step synthetic strategy illustrates the power of developing new initiators for AROP and the generality of this process for producing well-defined heterotelechelic PDMS derivatives. Furthermore, to highlight the fidelity and orthogonal nature of this approach, a range of “super-soft” bottlebrush networks were prepared using end-functionalized H–Si PDMS bottlebrushes and commercially available bis-vinyl PDMS cross-linker. These functional “super-soft” materials^58−61^ are of interest in a variety of applications ranging from high-sensitivity capacitive sensors^59^ to biological tissue mimics^62,63^ and efficient dielectric accuators.^64,65^

## Data Availability

Raw data are available from a permanent online repository at https://doi.org/10.5061/dryad.cjsxksncm.

## Supporting Information Available

Detailed experimental procedures, characterization (^1^H, ^13^C, ^29^Si-NMR, FT-IR, SEC, and MALDI), rheological measurements (PDF)
