# Supplementary material for: Heterotelechelic Silicones: Facile Synthesis and Functionalization Using Silane-Based Initiators
Source: Macromolecules. 2023 Oct 29;56(21):8806–12. doi: 10.1021/acs.macromol.3c01802 (PMC10653272; doi:10.1021/acs.macromol.3c01802)
Supplement: Supplementary file 1 — ma3c01802_si_001.pdf [file ma3c01802_si_001.pdf]

Supporting Information for:

Heterotelechelic silicones: Facile synthesis and functionalization using silane-based initiators

Yoichi Okayama,[†] Taejun Eom,[†] Michael Czuczola,[‡] Allison Abdilla,[‡] Jacob R. Blankenship,[‡] Kaitlin R. Albanese,[‡] Javier Read de Alaniz,[‡] Christopher M. Bates,[†‡§‖]* Craig J. Hawker[†‡§]*

[†]*Materials Research Laboratory,* [‡]*Department of Chemistry & Biochemistry,* [§]*Materials Department,* [‖]*Department of Chemical Engineering, University of California, Santa Barbara, CA 93106, USA,*

* To whom correspondence should be addressed: hawker@ucsb.edu

TABLE OF CONTENTS

Materials S2

Characterization methods S2

Synthesis procedures S3

Characterization data S11

References S51

Materials

Hexamethylcyclotrisiloxane (D_3, TCI – H0725, purity > 98%), ethylene glycol monoallyl ether (TCI – E0326, purity > 98%) were purchased from TCI America. D_3 was treated with calcium hydride (CaH_2, Aldrich, ≥ 97%) overnight at 80 °C followed by sublimation, then stored in dried hexanes under argon atmosphere. Methylene chloride (DCM, Sigma – 2700997, purity > 99.5%), lithium bis(trimethylsilyl)amide in hexanes solution (Sigma – 224367-100ML, 1 M), allyl glycidyl ether (Sigma – A32608, purity > 98%), vinyltrimethoxysilane (Sigma – 235768, purity > 98%), *N,N,N',N'',N''*-pentamethyldiethylenetriamine (PMDETA) (Sigma – 369497, purity > 99%), styrene (Sigma – S4972, purity > 99%), copper (I) bromide (Sigma – 254185, 99.999% trace metals basis) and xylenes (Sigma – 214736) divinyl malate (Sigma – 2912266, purity > 93%) were purchased from Sigma Aldrich. Platinum-divinyltetramethyldisiloxane complex (Karstedt's catalyst, Gelest – SIP6831.2, 2% Pt in xylene), 1,1,4,4-tetramethyl-1,4-disilabutane (Gelest – SIT7537.0, purity > 97%), 3-methacryloxypropyldimethylchlorosilane (Gelest – SIM6486.2, purity > 90%), 3-chloropropyldimethylchlorosilane (Gelest – SIC2336.0, purity > 97%), [(5-bicyclo[2.2.1]hept-2-enyl)ethyl]dimethylchlorosilane (Gelest – SIB0982.0, purity > 92%), vinyldimethylchlorosilane (Gelest – SIV9070.0, purity > 97%), and vinyl terminated polydimethylsiloxane (Gelest – DMS-V22) were purchased from Gelest. 3-methacryloxypropyldimethylchlorosilane, 3-chloropropyldimethylchlorosilane, [(5-bicyclo[2.2.1]hept-2-enyl)ethyl]dimethylchlorosilane, and vinyldimethylchlorosilane were treated by CaH_2 overnight at room temperature followed by distillation, then stored under argon atmosphere. Toluene (Fisher scientific – T3244, purity > 99.5%), hexanes (Fisher scientific – H292, purity > 98.5%), dimethyl formamide (Fisher scientific – D1030), acetonitrile (Fisher scientific – A998, purity > 99.9%), and ethyl vinyl ether (Fisher scientific – AC119082500, purity > 99%) were purchased from Fisher Scientific. Toluene, hexanes and DMF were further purified and dried by solvent purification system (PureSolv, Innovative Technology Inc.)

Characterization methods

^{1}H-, ^{13}C- and ^{29}Si-NMR spectra were collected on a Bruker Avance NEO 500 MHz using chloroform as a deuterated solvent. IR spectra was measured with a Thermo Nicolet iS10 FTIR spectrometer equipped with a Smart Diamond attenuated total reflectance (ATR) accessory. Size exclusion chromatography (SEC) was measured using Waters e2695 separation module with a Waters 2414 differential refractive index detector equipped with two columns (PLgel, 5 μm MiniMIX-D, 250×4.6 mm columns + guard) with chloroform containing 0.25% TEA at 35 °C as the mobile phase. MALDI-TOF MS spectrometry was measured using a Bruker Microflex LRF MALDI TOF mass spectrometer in positive reflection mode; the analyte, matrix (DCTB) were dissolved in chloroform at the concentration of 2.5 and 10 mg/mL respectively, and cationization agent (NaTFA) was dissolved in chloroform at concentrations of 1 mg/mL, then mixed in a volume ratio of 20 : 1 : 1 (DCTB : NaTFA : sample). 0.5 μL of this mixed solution was spotted onto a ground steel target plate and the solvent was allowed to evaporate prior to analysis. Frequency sweeps and isochronal temperature sweeps were obtained on a TA Instruments ARES G2 in oscillatory shear mode with an 8 mm parallel-plate geometry and a nitrogen-purged forced–convection oven.

Synthesis of hydride functionalized initiator (H–Si–C initiator) and preparation of 50 wt% hexanes solution.

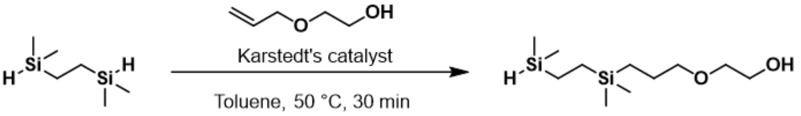

To a 250 mL round bottom flask, 1,1,4,4-tetramethyl-1,4-disilabutane (20.0 g, 136.6 mmol), and 2-allyloxyethanol (14.0 g, 136.6 mmol) were added and dissolved into 34 mL of toluene. Reaction mixture was degassed with argon for 10 minutes while stirring. 50 µL Karstedt's catalyst was added and stirred at 50 °C for 30 min. After this time, the organic solvent was removed under reduced pressure. The crude product was purified by silica gel column chromatography (eluent = EtOAc /Hex 10:90) and then distilled twice (120-130 °C, 200 mTorr) which gave about 17.7 g of the product as a colorless liquid (yield = 52 %)

^{1}H-NMR (500 MHz, $CDCl_3$) δ 3.88 – 3.79 (m, 1H), 3.79 – 3.71 (m, 2H), 3.59 – 3.53 (m, 2H), 3.45 (t, J = 7.0 Hz, 2H), 2.12 (s, 1H), 1.64 – 1.55 (m, 2H), 0.55 – 0.41 (m, 6H), 0.07 (d, J = 3.6 Hz, 6H), −0.02 (s, 6H). ^{13}C-NMR (126 MHz, $CDCl_3$) δ 74.31, 71.65, 61.91, 24.09, 10.68, 7.83, 6.36, −3.95, −4.83. ^{29}Si-NMR (99 MHz, $CDCl_3$) δ 4.49, −9.94. HRMS (ESI+) m/z calcd for $C_{11}H_{28}O_2Si_2Na$ [M+Na]+: 271.1526, observed: 271.1539.

General procedure for Si–H functionalized heterotelechelic PDMS from H–Si–C initiator. Synthesis of H–PDMS–MA targeting molar mass of 3.5 kg mol^{-1}.

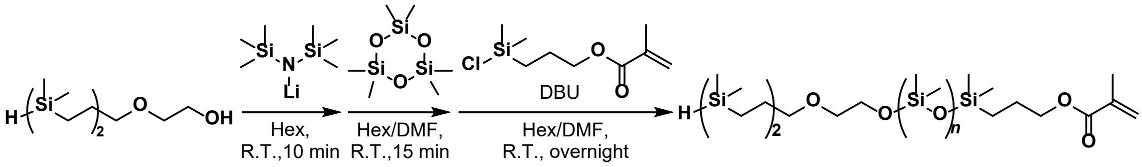

A 50 mL two neck round bottom flask equipped with stir bar was flame dried and purged with argon. To the flask, 50 wt% H–Si–C initiator in hexanes (0.27 mL, 0.40 mmol) solution and 1 M LiHMDS in hexanes (0.40 mL, 0.40 mmol) were added. After stirring for 10 minutes at room temperature, D_3 in hexanes (5.2 mL, 9.2 mmol) was added, then the polymerization was initiated by addition of dry DMF (0.41 mL, 15wt% to hexanes). After stirring for 15 minutes at room temperature, the polymerization was terminated by addition 3-methacryloxypropyldimethylchlorosilane (0.17 mL, 0.80 mmol) followed by addition of DBU (0.030 mL, 0.20 mmol) to catalyze termination reaction and the solution was kept stirring overnight. The polymerization solution was diluted with hexanes and washed with saturated $NaHCO_3$ aq. and water. The organic layer was dried with $MgSO_4$ then concentrated. The oily residue was washed with acetonitrile then dried under vacuum to obtain purified heterotelechelic PDMS. (H–PDMS–MA).

H–PMDS–Cl, –Nb, –Vinyl were synthesized in the same manner, except for the polymerizations were terminated by 3-chloropropyldimethylchlorosilane (0.13 mL, 0.80 mmol), [(5-Bicyclo[2.2.1]hept-2-enyl)ethyl]dimethylchlorosilane (0.17 mL, 0.80 mmol), or vinyldimethylchlorosilane (0.11 mL, 0.80 mmol) respectively.

H–PDMS–MA: ^{1}H-NMR (500 MHz, $CDCl_3$) δ 6.10 (dt, J = 2.1, 1.0 Hz, C(**CH_2**)COO), 5.54 (p, J = 1.6 Hz, C(**CH_2**)COO), 4.11 (t, J = 6.9 Hz, COO**CH_2**CH_2), 3.91 – 3.74 (m, **HSi**,

$OCH_2\mathbf{CH_2}OSi$), 3.52 (t, J = 5.5 Hz, $O\mathbf{CH_2}CH_2OSi$), 3.42 (t, J = 7.0 Hz, $SiCH_2CH_2\mathbf{CH_2}OCH_2$), 1.95 (t, J = 1.3 Hz, $\mathbf{CH_3}C(CH_2)COO$), 1.79 – 1.64 (m, $COOCH_2\mathbf{CH_2}CH_2Si$), 1.57 (ddt, J = 11.2, 9.1, 6.1 Hz, $SiCH_2\mathbf{CH_2}CH_2OCH_2$), 0.63 – 0.53 (m, $COOCH_2CH_2\mathbf{CH_2}Si$), 0.53 – 0.37 (m, $HSi(CH_3)_2\mathbf{C_2H_4}Si(CH_3)_2\mathbf{CH_2}$), 0.32 – −0.22 (br m, $Si\mathbf{(CH_3)_2}$). ^{13}C-NMR (126 MHz, $CDCl_3$) δ 167.62, 136.75, 125.22, 74.59, 72.05, 67.29, 61.71, 24.27, 22.80, 18.49, 14.27, 10.83, 8.00, 6.52, 1.20, 0.24, −0.81, −3.80, −4.70. ^{29}Si-NMR (99 MHz, $CDCl_3$) δ 7.33, 4.44, −10.03, −11.73, −21.96. SEC: M_n = 3.3 kDa, M_w = 3.9 kDa, $Đ$ = 1.16.

H–PDMS–Cl: ^{1}H-NMR (500 MHz, $CDCl_3$) δ 3.94 – 3.73 (m, **HSi**, $OCH_2\mathbf{CH_2}OSi$), 3.62 – 3.49 (m, $O\mathbf{CH_2}CH_2OSi$, $Cl\mathbf{CH_2}$), 3.44 (t, J = 7.1 Hz, $SiCH_2CH_2\mathbf{CH_2}OCH_2$), 1.93 – 1.80 (m, $ClCH_2\mathbf{CH_2}$), 1.60 (ddt, J = 11.3, 9.1, 7.1 Hz, $SiCH_2\mathbf{CH_2}CH_2OCH_2$), 0.72 – 0.61 (m, $ClCH_2CH_2\mathbf{CH_2}Si$), 0.56 – 0.41 (m, $HSi(CH_3)_2\mathbf{C_2H_4}Si(CH_3)_2\mathbf{CH_2}$), 0.28 – −0.14 (br m, $Si\mathbf{(CH_3)_2}$). ^{13}C-NMR (126 MHz, $CDCl_3$) δ 74.59, 72.05, 61.71, 47.94, 27.23, 24.27, 16.02, 10.83, 8.00, 6.52, 1.20, 0.27, −0.81, −3.80, −4.70. ^{29}Si-NMR (99 MHz, $CDCl_3$) δ 7.04, 4.44, −10.02, −11.73, −21.96. SEC: M_n = 2.7 kDa, M_w = 3.2 kDa, $Đ$ = 1.17.

H–PDMS–Nb: ^{1}H-NMR (500 MHz, $CDCl_3$) δ 6.22 – 5.75 (m, $CH\mathbf{CH{=}CH}CH(CH_2)$), 3.89 – 3.75 (m, **HSi**, $OCH_2\mathbf{CH_2}OSi$), 3.53 (t, J = 5.4 Hz, $O\mathbf{CH_2}CH_2OSi$), 3.42 (t, J = 7.0 Hz, $SiCH_2CH_2\mathbf{CH_2}OCH_2$), 2.91 – 2.45 (m, $\mathbf{CH}CH{=}CH\mathbf{CH}(CH_2)$), 2.01 – 1.83 (m, $CHCH{=}CHCH(\mathbf{CH_2})$), 1.62 – 1.53 (m, $SiCH_2\mathbf{CH_2}CH_2OCH_2$), 1.43 – 1.18 (m, $\mathbf{CH_2CH}CH_2CH_2Si$), 1.16 – 1.01 (m, $CH\mathbf{CH_2}CH_2Si$), 0.64 – 0.40 (m, $HSi(CH_3)_2\mathbf{C_2H_4}Si(CH_3)_2\mathbf{CH_2}$, $Si\mathbf{CH_2}CH_2CH$), 0.26 – −0.14 (br m, $Si\mathbf{(CH_3)_2}$). ^{13}C-NMR (126 MHz, $CDCl_3$) δ 136.94, 136.90, 136.22, 132.25, 74.41, 71.88, 61.54, 49.51, 45.94, 45.11, 45.00, 42.56, 42.25, 42.18, 41.81, 33.05, 32.36, 29.83, 28.02, 24.10, 17.48, 17.16, 10.66, 7.82, 6.35, 1.02, 0.11, 0.06, −0.99, −3.98, −4.88. ^{29}Si-NMR (99 MHz, $CDCl_3$) δ 7.49, 4.43, −10.04, −11.73, −21.96. SEC: M_n = 3.8 kDa, M_w = 4.4 kDa, $Đ$ = 1.16.

H–PDMS–vinyl: ^{1}H-NMR (500 MHz, $CDCl_3$) δ 6.20 – 6.03 (m, $CH_2\mathbf{CH}Si$), 5.93 (dd, J = 14.8, 3.9 Hz, $\mathbf{CH_2}CHSi$), 5.78 – 5.70 (m, $\mathbf{CH_2}CHSi$), 3.85 – 3.78 (m, **HSi**, $OCH_2\mathbf{CH_2}OSi$), 3.53 (t, J = 5.5 Hz, $O\mathbf{CH_2}CH_2OSi$), 3.42 (t, J = 7.0 Hz, $SiCH_2CH_2\mathbf{CH_2}OCH_2$), 1.64 – 1.51 (m, $SiCH_2\mathbf{CH_2}CH_2OCH_2$), 0.59 – 0.37 (m, $HSi(CH_3)_2\mathbf{C_2H_4}Si(CH_3)_2\mathbf{CH_2}$), 0.38 – −0.38 (br m, $Si\mathbf{(CH_3)_2}$). ^{13}C-NMR (126 MHz, $CDCl_3$) δ 139.33, 131.60, 74.42, 71.88, 61.54, 24.10, 10.65, 7.83, 6.35, 1.03, 0.24, −0.99, −3.97, −4.87. ^{29}Si-NMR (99 MHz, $CDCl_3$) δ 4.45, −4.13, −10.02, −11.73, −20.15 – −23.46. SEC: M_n = 2.9 kDa, M_w = 3.4 kDa, $Đ$ = 1.16.

General procedure for Si–H functionalized heterotelechelic PDMS from H–Si–O initiator. Synthesis of H–PDMS–MA targeting molar mass of 3.5 kg mol^{-1}.

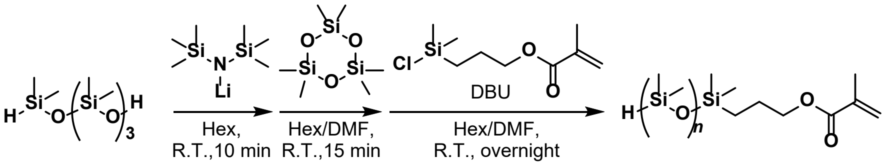

H–Si–O initiator was synthesized with modified previously reported method and stored in dried hexanes under argon atmosphere.[1] A 50 mL two neck round bottom flask equipped with stir bar was flame dried and purged with argon. To the flask, 50 wt% H–Si–O initiator in hexanes (0.30 mL, 0.40 mmol) solution and 1 M LiHMDS in hexanes (0.40 mL, 0.40 mmol) were added. After stirring for 10 minutes at room temperature, D_3 in hexanes (5.2 mL, 9.2 mmol) was added,

then the polymerization was initiated by addition of dry DMF (0.41 mL, 15 wt% in hexanes). After stirring for 15 minutes at room temperature, the polymerization was terminated by addition of 3-methacryloxypropyldimethylchlorosilane (0.17 mL, 0.80 mmol) and the solution was kept stirring overnight. The polymerization solution was diluted with hexanes and washed with saturated $NaHCO_3$ aq., then water. The organic layer was dried with $MgSO_4$ then concentrated. The oily residue was washed with acetonitrile then dried under vacuum to obtain purified heterotelechelic PDMS (H–PDMS–MA).

^{1}H-NMR (500 MHz, $CDCl_3$) δ 6.10 (dd, J = 1.8, 0.9 Hz, C(**CH_2**)COO), 5.54 (q, J = 1.6 Hz, C(**CH_2**)COO), 4.71 (hept, J = 2.8 Hz, HSi), 4.11 (t, J = 6.9 Hz, COO**CH_2**CH_2), 1.95 (t, J = 1.3 Hz, **CH_3**C(CH_2)COO), 1.77 – 1.67 (m, COOCH_2**CH_2**CH_2Si), 0.67 – 0.50 (m, COOCH_2CH_2**CH_2**Si), 0.32 – −0.16 (br m, Si**(CH_3)$_2$**). ^{13}C-NMR (126 MHz, $CDCl_3$) δ 167.62, 136.75, 125.22, 74.59, 72.05, 67.29, 61.71, 24.27, 22.80, 18.49, 14.27, 10.83, 7.99, 6.52, 1.20, 0.24, −0.81, −3.80, −4.70. ^{29}Si-NMR (99 MHz, $CDCl_3$) δ 7.34, −6.93, −21.95. SEC: M_n = 3.3 kDa, M_w = 3.9 kDa, $Đ_M$ = 1.19.

General procedure for hydrosilylation of heterotelechelic PDMS. Synthesis of 3.3 kDa HO–PDMS–MA.

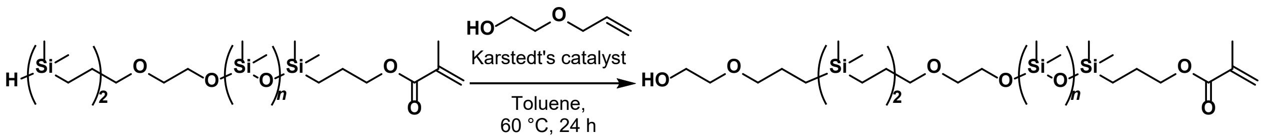

To a 4 mL glass vial equipped with stir bar, H–PDMS–MA ($M_{n,\,NMR}$ = 3.3 kg mol^{-1}, 211 mg, 0.064 mmol), 2-allyloxyethanol (10 mg, 0.096 mmol), 0.5 mL dry toluene were added. Reaction mixture was degassed with argon for 10 minutes while stirring. 10 µL Karstedt's catalyst was added and vial heated at 60 °C using an aluminum heat block for 24 h. Reaction solution was passed through a silica gel plug then solvent removed under vacuum. The oily residue was washed with acetonitrile then dried under vacuum to obtain purified heterotelechelic PDMS. (HO–PDMS–MA).

Epoxy–, $(MeO)_3$Si–PDMS–MA were synthesized in a same manner, excepted for using allyl glycidyl ether (11 mg, 0.096 mmol), or vinyltrimethoxysilane (14 mg, 0.096 mmol) as terminal alkenes. ATRP–PDMS–Cl was also synthesized via hydrosilylation of H–PDMS–Cl ($M_{n,\,NMR}$ = 2.5 kg mol^{-1}, 300 mg, 0.12 mmol) with 2-(allyloxy)ethyl 2-bromo-2-methylpropanoate (45 mg, 0.18 mmol), which was synthesized by previously reported procedure, in the same manner with HO–PDMS–MA.[2]

HO–PDMS–MA: ^{1}H-NMR (500 MHz, $CDCl_3$) δ 6.12 (dt, J = 2.1, 1.0 Hz, C(**CH_2**)COO), 5.56 (p, J = 1.6 Hz, C(**CH_2**)COO), 4.13 (t, J = 6.9 Hz, COO**CH_2**CH_2), 3.83 (t, J = 5.5 Hz, OCH_2**CH_2**OSi), 3.80 – 3.71 (m, OH**CH_2**CH_2OCH_2), 3.60 – 3.52 (m, OHCH_2**CH_2**OCH_2, O**CH_2**CH_2OSi), 3.45 (dt, J = 8.3, 7.0 Hz, OH(CH_2)$_2$O**CH_2**, SiCH_2CH_2**CH_2**OCH_2), 2.08 (s, **OH**(CH_2)$_2$OCH_2), 1.97 (t, J = 1.3 Hz, **CH_3**C(CH_2)COO), 1.77 – 1.67 (m, COOCH_2**CH_2**CH_2Si), 1.60 (dddd, J = 14.4, 9.3, 7.0, 3.6 Hz, **CH_2**CH_2Si(CH_3)$_2C_2H_4$Si(CH_3)$_2CH_2$**CH_2**), 0.64 – 0.56 (m, COOCH_2CH_2**CH_2**Si), 0.55 – 0.46 (m, **CH_2**Si(CH_3)$_2C_2H_4$Si(CH_3)$_2$**CH_2**), 0.40 (d, J = 5.4 Hz, Si**C_2H_4**Si), 0.28 – −0.09 (br m, Si**(CH_3)$_2$**). ^{13}C-NMR (126 MHz, $CDCl_3$) δ 167.47, 136.57, 125.06, 74.45, 74.30, 71.88, 71.67, 67.12, 61.86, 61.52, 24.10, 22.62, 18.32, 14.10, 10.60, 7.09, 1.02, 0.06, −0.99, −4.03. ^{29}Si-NMR (99 MHz, $CDCl_3$) δ 7.34, 4.47, −11.70, −21.95. SEC: M_n = 3.4 kDa, M_w = 3.4 kDa, $Đ$ = 1.28.

Epoxy–PDMS–MA: ^{1}H-NMR (500 MHz, $CDCl_3$) δ 6.10 (dd, J = 1.8, 1.0 Hz, $C(\mathbf{CH_2})COO$), 5.53 (p, J = 1.6 Hz, $C(\mathbf{CH_2})COO$), 4.10 (t, J = 6.9 Hz, $COO\mathbf{CH_2}CH_2$), 3.80 (t, J = 5.5 Hz, $OCH_2\mathbf{CH_2}OSi$), 3.70 (dd, J = 11.5, 3.1 Hz, $OCH_2CH\mathbf{CH_2}$), 3.52 (t, J = 5.5 Hz, $O\mathbf{CH_2}CH_2OSi$), 3.50 – 3.31 (m, $OCH_2CH\mathbf{CH_2}$, $OCH_2CHCH_2O\mathbf{CH_2}$, $SiCH_2CH_2\mathbf{CH_2}OCH_2$), 3.14 (ddt, J = 5.8, 4.1, 2.9 Hz, $OCH_2\mathbf{CH}CH_2$), 2.79 (dd, J = 5.1, 4.1 Hz, $O\mathbf{CH_2}CHCH_2$), 2.60 (dd, J = 5.0, 2.7 Hz, $O\mathbf{CH_2}CHCH_2$), 1.94 (t, J = 1.3 Hz, $\mathbf{CH_3}C(CH_2)COO$), 1.77 – 1.64 (m, 3 $COOCH_2\mathbf{CH_2}CH_2Si$), 1.64 – 1.52 (m, $\mathbf{CH_2}CH_2Si(CH_3)_2C_2H_4Si(CH_3)_2CH_2\mathbf{CH_2}$), 0.61 – 0.55 (m, $COOCH_2CH_2\mathbf{CH_2}Si$), 0.55 – 0.44 (m, $\mathbf{CH_2}Si(CH_3)_2C_2H_4Si(CH_3)_2\mathbf{CH_2}$), 0.37 (s, $Si\mathbf{C_2H_4}Si$), 0.31 – −0.16 (br m, $Si\mathbf{(CH_3)_2}$). ^{13}C-NMR (126 MHz, $CDCl_3$) δ 167.60, 136.74, 125.20, 74.77, 74.61, 72.06, 71.64, 67.28, 61.70, 51.03, 44.47, 24.30, 22.79, 18.48, 14.27, 10.77, 7.26, 1.19, 0.23, −0.82, −3.87. ^{29}Si-NMR (99 MHz, $CDCl_3$) δ 7.32, 4.46, −11.74, −21.97. SEC: M_n = 3.6 kDa, M_w = 4.4 kDa, $Đ$ = 1.21.

$(MeO)_3Si$–PDMS–MA: ^{1}H-NMR (500 MHz, $CDCl_3$) δ 6.10 (q, J = 1.2 Hz, $C(\mathbf{CH_2})COO$), 5.53 (p, J = 1.6 Hz, $C(\mathbf{CH_2})COO$), 4.10 (t, J = 6.9 Hz, $COO\mathbf{CH_2}CH_2$), 3.80 (t, J = 5.5 Hz, $OCH_2\mathbf{CH_2}OSi$), 3.57 (s, $\mathbf{(CH_3O)_3}Si$), 3.52 (t, J = 5.5 Hz, $O\mathbf{CH_2}CH_2OSi$), 3.41 (t, J = 7.0 Hz, $SiCH_2CH_2\mathbf{CH_2}OCH_2$), 1.94 (t, J = 1.3 Hz, $\mathbf{CH_3}C(CH_2)COO$), 1.76 – 1.64 (m, $COOCH_2\mathbf{CH_2}CH_2Si$), 1.62 – 1.51 (m, $SiCH_2\mathbf{CH_2}CH_2OCH_2$), 0.62 – 0.55 (m, $COOCH_2CH_2\mathbf{CH_2}Si$), 0.53 (s, $(CH_3O)_3Si\mathbf{CH_2CH_2}Si$), 0.50 – 0.43 (m, $Si\mathbf{CH_2}CH_2CH_2OCH_2$), 0.37 (s, $Si\mathbf{C_2H_4}Si$), 0.24 – −0.20 (br m, $Si\mathbf{(CH_3)_2}$). ^{13}C-NMR (126 MHz, $CDCl_3$) δ 167.61, 136.75, 125.21, 74.64, 72.06, 67.28, 61.71, 50.74, 24.29, 22.80, 18.49, 14.27, 10.79, 7.27, 6.70, 5.68, 1.19, 0.23, −0.82, −3.86, −4.44. ^{29}Si-NMR (99 MHz, $CDCl_3$) δ 7.33, 5.96, 4.45, −11.74, −21.97, −41.57. SEC: M_n = 4.0 kDa, M_w = 4.6 kDa, $Đ$ = 1.15.

ATRP–PDMS–Cl: ^{1}H-NMR (500 MHz, $CDCl_3$) δ 4.40 – 4.27 (m, $BrC(CH_3)_2COO\mathbf{CH_2}$), 3.80 (t, J = 5.5 Hz, $OCH_2\mathbf{CH_2}OSi$), 3.72 – 3.62 (m, $BrC(CH_3)_2COOCH_2\mathbf{CH_2}$), 3.57 – 3.47 (m, $O\mathbf{CH_2}CH_2OSi$, $Cl\mathbf{CH_2}$), 3.43 (dt, J = 12.5, 7.0 Hz, $COOCH_2CH_2O\mathbf{CH_2}$, $SiCH_2CH_2\mathbf{CH_2}OCH_2$), 1.94 (s, $BrC\mathbf{(CH_3)_2}COO$), 1.86 – 1.76 (m, $ClCH_2\mathbf{CH_2}$), 1.64 – 1.49 (m, $\mathbf{CH_2}CH_2Si(CH_3)_2C_2H_4Si(CH_3)_2CH_2\mathbf{CH_2}$), 0.72 – 0.61 (m, $ClCH_2CH_2\mathbf{CH_2}Si$), 0.54 – 0.42 (m, $\mathbf{CH_2}Si(CH_3)_2C_2H_4Si(CH_3)_2\mathbf{CH_2}$), 0.41 (s, $Si\mathbf{C_2H_4}Si$), 0.29 – −0.19 (br m, $Si\mathbf{(CH_3)_2}$). ^{13}C-NMR (126 MHz, $CDCl_3$) δ 171.80, 74.61, 74.54, 72.05, 68.29, 65.34, 61.69, 55.76, 47.92, 30.94, 27.21, 24.27, 16.00, 10.76, 7.26, 1.19, 0.26, −0.82. ^{29}Si-NMR (99 MHz, $CDCl_3$) δ 7.03, 4.46, 4.45, −11.74, −21.58. SEC: M_n = 3.1 kDa, M_w = 3.6 kDa, $Đ$ = 1.16.

Synthesis of PS–*b*–PDMS–Cl targeting molar mass of 4.2 kg mol^{-1}.

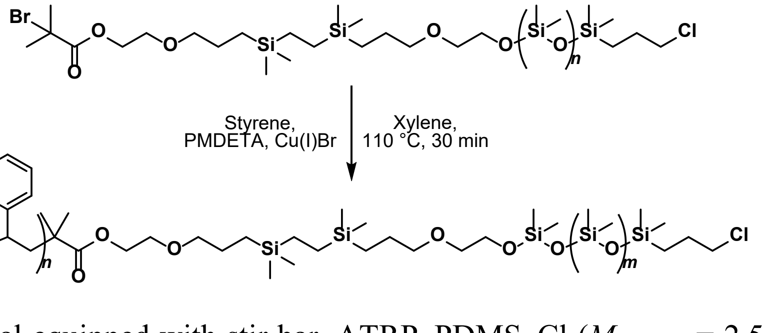

To a 4 mL glass vial equipped with stir bar, ATRP–PDMS–Cl ($M_{n,\,NMR}$ = 2.5 kg mol^{-1}, 100 mg, 0.04 mmol), styrene (500 mg, 4.8 mmol), PMDETA (13 mg, 0.08 mmol), 0.6 mL dry xylene were added. Reaction mixture was degassed with argon for 15 minutes while stirring. Cu(I)Br (6 mg, 0.04 mmol) was added and vial heated at 110 °C using an aluminum heat block

for 30 min. Reaction solution was passed through a silica gel plug then solvent removed under vacuum. The crude sample was precipitated with methanol 3 times then dried under high vacuum conditions to obtain purified solid.

^{1}H-NMR (500 MHz, $CDCl_3$) δ 7.36 – 6.30 (br m, PS), 3.82 (t, J = 1.7 Hz, $OCH_2\mathbf{CH_2}OSi$), 3.56 – 3.50 (m, $O\mathbf{CH_2}CH_2OSi$, $Cl\mathbf{CH_2}$), 3.46 – 3.26 (m, $BrC(CH_3)_2COOCH_2\mathbf{CH_2}$, $COOCH_2CH_2O\mathbf{CH_2}$, $SiCH_2CH_2\mathbf{CH_2}OCH_2$), 2.62 – 0.82 (br m, PS, $BrC\mathbf{(CH_3)_2}COO$, $ClCH_2\mathbf{CH_2}$, $\mathbf{CH_2}CH_2Si(CH_3)_2C_2H_4Si(CH_3)_2CH_2\mathbf{CH_2}$), 0.78 – 0.59 (m, $ClCH_2CH_2\mathbf{CH_2}Si$), 0.59 – 0.43 (m, $\mathbf{CH_2}Si(CH_3)_2C_2H_4Si(CH_3)_2\mathbf{CH_2}$), 0.39 (s, $Si\mathbf{C_2H_4}Si$), 0.30 – -0.13 (br m, $Si\mathbf{(CH_3)_2}$). ^{13}C-NMR (126 MHz, $CDCl_3$) δ 177.27, 145.52, 131.24 – 121.36 (m), 74.48, 74.23 (d, J = 4.2 Hz), 71.91, 68.33, 63.19, 61.55, 47.86, 46.51 – 39.34 (m), 27.08, 24.13, 15.87, 10.62, 7.13, 1.08, 0.14, -0.93, -4.00. ^{29}Si-NMR (99 MHz, $CDCl_3$) δ 7.03, 4.46, 4.45, −11.74, −21.58. SEC: M_n = 5.5 kDa, M_w = 6.0 kDa, $Đ$ = 1.09.

General procedure for monofunctionalized PDMS. Synthesis of Bu–PDMS–Nb targeting molar mass of 3.5 kg mol^{-1}.

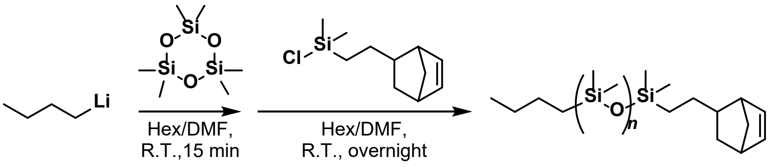

A 50 mL two neck round bottom flask equipped with stirrer bar was flame dried and purged with argon. To the flask, *n*-butyl lithium in hexanes (1.6 M, 0.63 mL, 1.0 mmol) and D_3 in hexanes (1.7 M, 13.3 mL, 23 mmol) were added, then the polymerization was initiated by addition of 1.06 mL dry DMF. After stirring for 15 minutes at room temperature, the polymerization was terminated by addition of [(5-Bicyclo[2.2.1]hept-2-enyl)ethyl]dimethylchlorosilane (0.43 mL, 2.0 mmol). The reaction solution was kept stirring overnight. The polymerization solution was diluted with hexanes and washed with saturated $NaHCO_3$ aq., then water. The organic layer was dried with $MgSO_4$ then concentrated. The oily residue was washed with acetonitrile then dried under vacuum to obtain purified monofunctionalized PDMS. (Bu–PDMS–Nb).

^{1}H-NMR (500 MHz, $CDCl_3$) δ 6.18 – 5.76 (m, $CH\mathbf{CH{=}CH}CH(CH_2)$), 2.89 – 2.45 (m, $\mathbf{CH}CH{=}CH\mathbf{CH}(CH_2)$), 2.01 – 1.83 (m, $CHCH{=}CHCH(\mathbf{CH_2})$), 1.44 – 1.19 (m, $CH_3\mathbf{(CH_2)_2}CH_2Si$, $\mathbf{CH_2CH}CH_2CH_2Si$), 1.17 – 1.01 (m, $SiCH_2\mathbf{CH_2}CH$), 0.89 (t, $\mathbf{CH_3}(CH_2)_2CH_2Si$), 0.64 – 0.44 (m, $CH_3(CH_2)_2\mathbf{CH_2}Si$, $Si\mathbf{CH_2}CH_2CH$), 0.24 – −0.10 (br m, $Si\mathbf{(CH_3)_2}$). ^{13}C-NMR (126 MHz, $CDCl_3$) δ 136.94, 136.90, 136.23, 132.26, 49.52, 45.95, 45.12, 45.00, 42.57, 42.25, 42.19, 41.81, 33.05, 32.37, 29.83, 28.03, 26.36, 25.45, 17.95, 17.49, 17.17, 13.79, 1.02, 0.16, 0.11, 0.06. ^{29}Si-NMR (99 MHz, $CDCl_3$) δ 7.59, 7.50, −21.95. SEC: M_n = 3.9 kDa, M_w = 4.8 kDa, $Đ$ = 1.21.

General procedure for ring-opening metathesis polymerization (ROMP) on norbornene functionalized PDMS. Homo polymerization of H–PDMS–Nb targeting DP of 50.

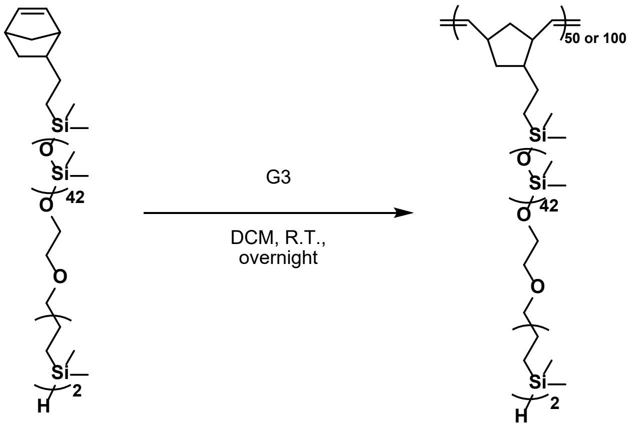

Grubbs' third-generation metathesis catalyst [(H_2IMes)(pyr)$_2$(Cl)2Ru=CHPh] (G3) was prepared according to literature.[3] ROMPs were conducted in glove box under nitrogen atmosphere. To a 20 mL glass vial equipped with stir bar, H–PDMS–Nb ($M_{n,\ NMR}$ = 3.5 kg mol^{-1}, 109 mg, 31 μmol) was added and dissolved into 2.2 mL of DCM. To the polymer solution, G3 in DCM (G3, 2.3 mg mL^{-1}, 0.20 mL, 0.62 μmol) was quickly added to initiate polymerization. After overnight stirring, ethyl vinyl ether was added to quench the reaction. The polymerization solution was dried under vacuum to remove excess amount of ethyl vinyl ether and solvent. Small amount of sample was taken to measure SEC of crude sample to confirm bottlebrush polymer formation as well as to calculate the conversion. The crude sample was further purified by washing with acetonitrile 3 times then dried under high vacuum conditions to obtain purified bottlebrush polymer.

Homo polymerization of H–PDMS–Nb targeting DP = 100 was conducted in the same manner except for changing the amount of G3 in DCM (2.3 mg mL^{-1}, 0.10 mL, 0.31 μmol).

^{1}H-NMR (500 MHz, $CDCl_3$) δ 5.53 – 4.92 (br m, norbornene backbone), 3.88 – 3.74 (m, **HSi**, OCH_2**CH_2**OSi), 3.52 (t, *J* = 5.5 Hz, O**CH_2**CH_2OSi), 3.42 (t, *J* = 7.0 Hz, O**CH_2**CH_2OSi), 3.08 – 1.71 (br m, norbornene backbone), 1.62 – 1.53 (m, $SiCH_2$**CH_2**CH_2OCH_2), 1.50 – 1.30 ($HSi(CH_3)$**C_2H_4**$Si(CH_3)_2$, **CH_2**$COOCH_2CH_2$**CH_2**Si, $CHCH_2$**CH_2**Si), 1.23 – 0.87 (br m, norbornene backbone), 0.67 – 0.31 (m, $HSi(CH_3)_2$**C_2H_4**$Si(CH_3)_2$**CH_2**, Si**CH_2**CH_2CH), 0.24 – −0.10 (br m, Si**$(CH_3)_2$**). ^{13}C-NMR (126 MHz, $CDCl_3$) δ 74.57, 72.02, 61.68, 24.24, 10.80, 7.98, 6.50, 1.17 (d, *J* = 2.0 Hz), −0.83, −3.81, −4.71. ^{29}Si-NMR (99 MHz, $CDCl_3$) δ 4.45, −10.00, −11.69, −21.95.

General procedure for ROMP on norbornene functionalized PDMS. Copolymerization of Bu–PDMS–Nb and H–PDMS–Nb targeting DP of 100.

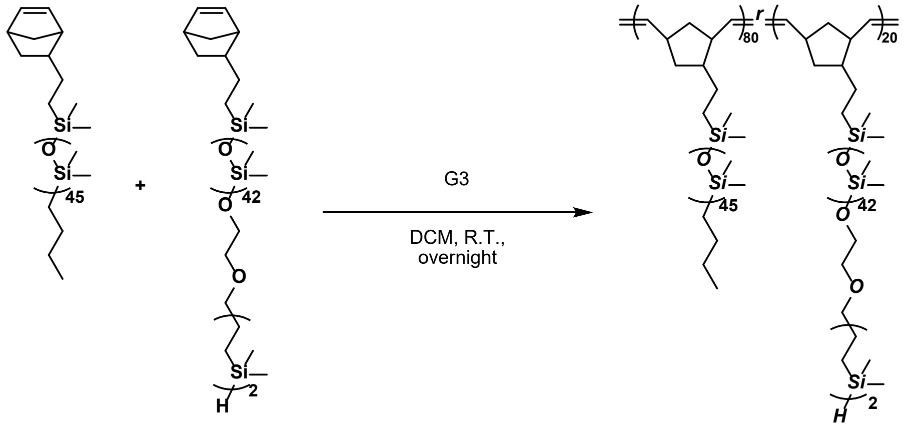

ROMPs were conducted in grove box under nitrogen atmosphere. To a 20 mL glass vial equipped with stirrer bar, Bu–PDMS–Nb ($M_{n,\,NMR}$ = 3.8 kg mol^{-1}, 1000 mg, 263 µmol) H–PDMS–Nb ($M_{n,\,NMR}$ = 3.5 kg mol^{-1}, 230 mg, 66 µmol) was added and dissolved into 5.0 mL of DCM. To the polymer solution, G3 in DCM (12 mg mL^{-1}, 0.20 mL, 3.3 µmol) was quickly added to initiate polymerization. After overnight stirring, ethyl vinyl ether was added to quench the reaction. The polymerization solution was dried under vacuum to remove excess amount of ethyl vinyl ether and solvent. Slight amount of sample was taken to measure SEC of crude sample in order to confirm bottlebrush polymer formation as well as to calculate the conversion. The crude sample was further purified by washing with acetonitrile 3 times then dried under high vacuum conditions to obtain purified bottlebrush polymer.

^{1}H-NMR (500 MHz, CDCl3) δ 5.29 (br m, norbornene backbone), 3.88 – 3.74 (m, **HSi**, OCH_2**CH_2**OSi), 3.52 (t, *J* = 5.5 Hz, O**CH_2**CH_2OSi), 3.42 (t, *J* = 7.0 Hz, O**CH_2**CH_2OSi), 3.14 – 1.70 (br m, norbornene backbone), 1.62 – 1.53 (m, SiCH_2**CH_2**CH_2OCH_2), 1.42 – 1.24 (m, CH_3**$(CH_2)_2$**CH_2Si), 1.20 – 0.97 (br m, norbornene backbone), 0.89 (t, J = 6.8 Hz, **CH_3**$(CH_2)_2CH_2$Si), 0.69 – 0.38 (m, $CH_3(CH_2)_2$**CH_2**Si, HSi$(CH_3)_2$**C_2H_4**Si$(CH_3)_2$**CH_2**, Si**CH_2**CH_2CH), 0.24 – −0.10 (br m, Si**$(CH_3)_2$**). ^{13}C-NMR (126 MHz, $CDCl_3$) δ 74.42, 71.88, 61.54, 26.36, 25.45, 24.10, 17.95, 13.79, 10.65, 7.83, 6.35, 1.03, 0.16, −0.98, −3.97, −4.87. ^{29}Si-NMR (99 MHz, $CDCl_3$) δ 7.59, 4.45, −10.01, −11.69, −21.95.

Procedure for post polymerization crosslinking for bottlebrush PDMS network.

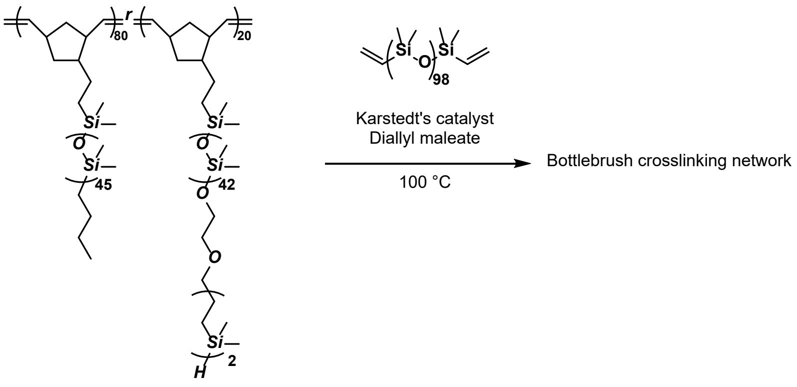

To a 4 mL glass vial, bottlebrush copolymer of Bu–PDMS–Nb and H–PDMS–Nb (150 mg, 0.39 µmol), divinyl terminated PDMS (DMS-V22, $M_{n,\,NMR}$ = 7.4 kg mol^{-1}, 5.9 mg, 0.79 µmol) and diallyl maleate in DCM (10wt%, 1.2 mg, 0.61 µmol) were added. After mixing homogeneously, Karstedt's catalyst (2wt% Pt in xylene, 0.45 µL, 0.02 µmol) was added. For the mold curing, the mixed polymer was poured into an 8 mm diameter mold and cured at 100 °C for 2 hours. For curing profile measurement, the mixed polymer was loaded on to rheometer and viscoelasticity was monitored while temperature was kept at 100 °C after increasing from R.T.

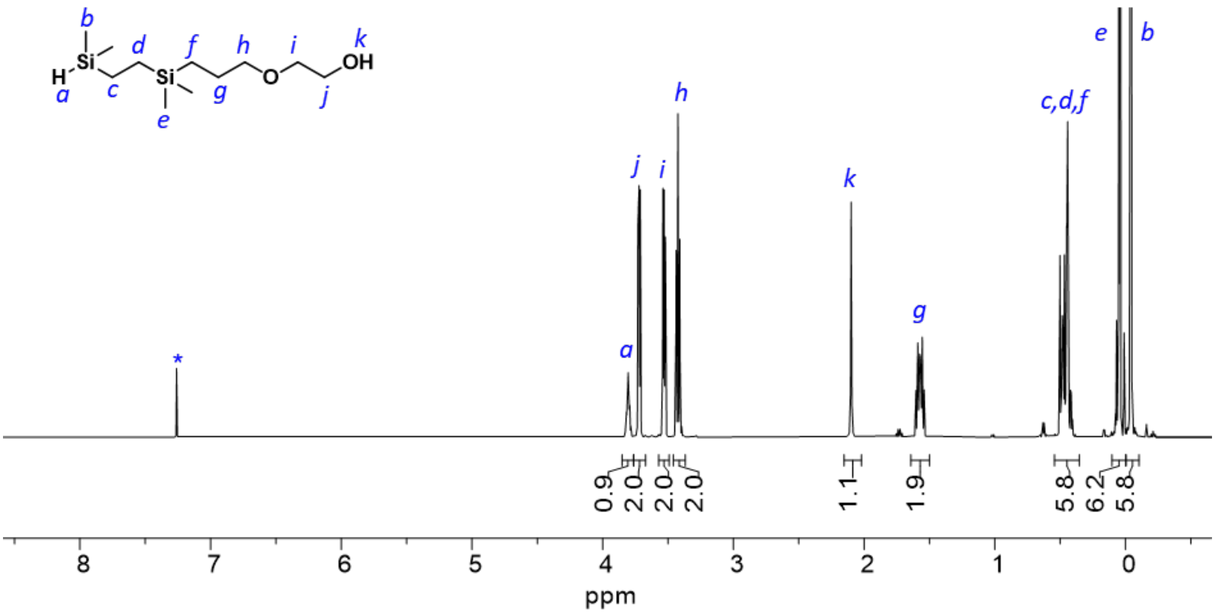

Figure S1. ^{1}H-NMR of H–Si–C initiator in deuterated chloroform.

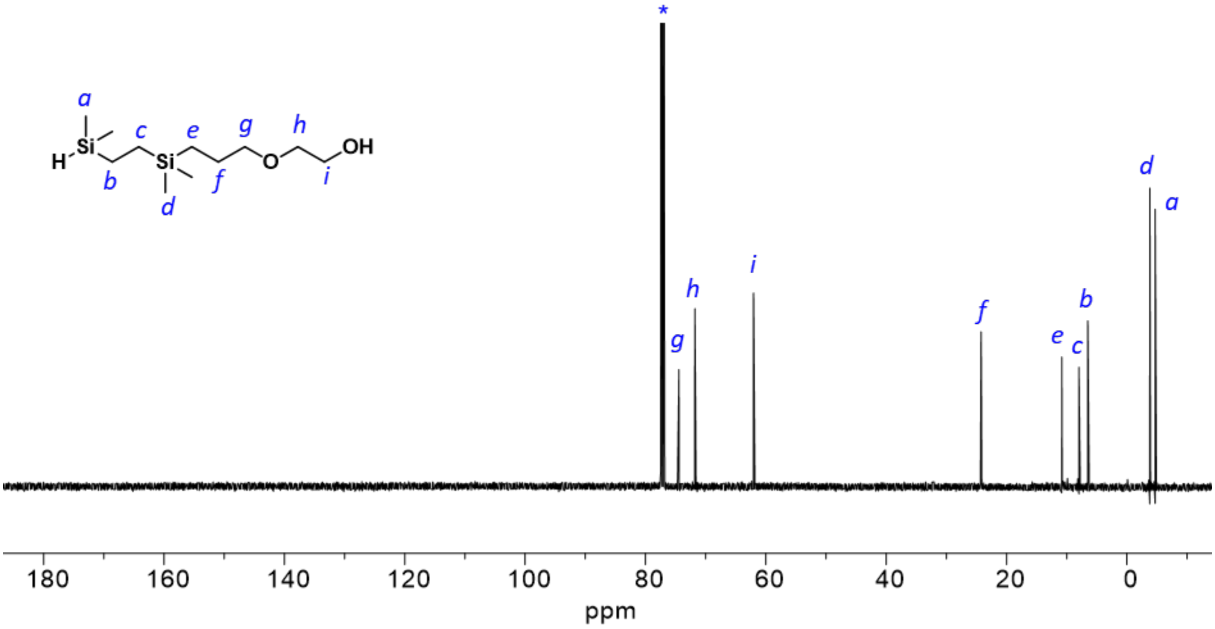

Figure S2. ^{13}C-NMR of H–Si–C initiator in deuterated chloroform.

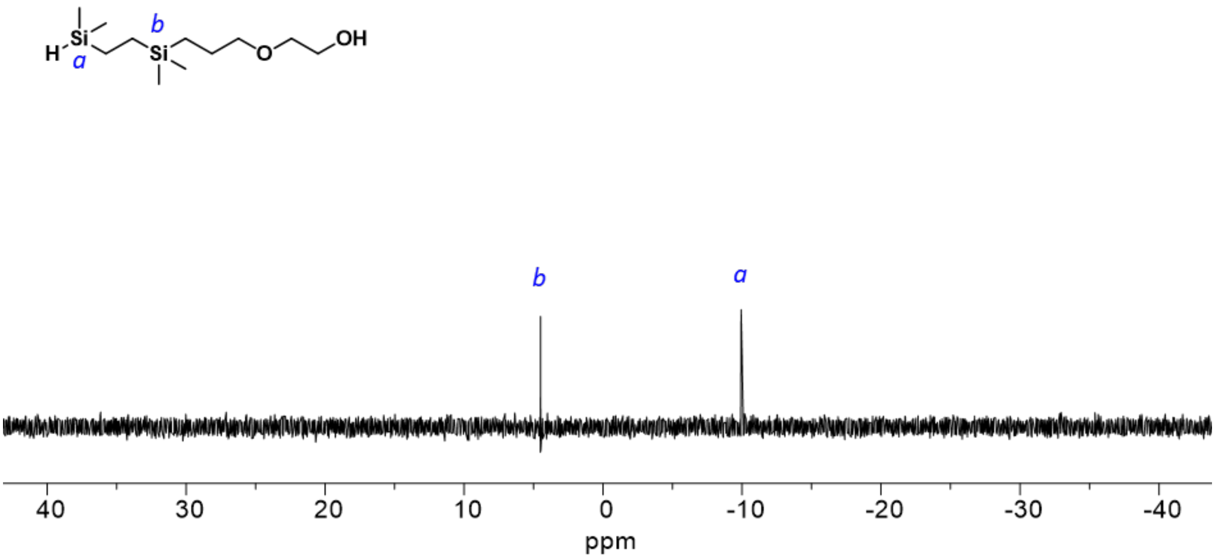

Figure S3. ^{29}Si-NMR of H–Si–C initiator in deuterated chloroform.

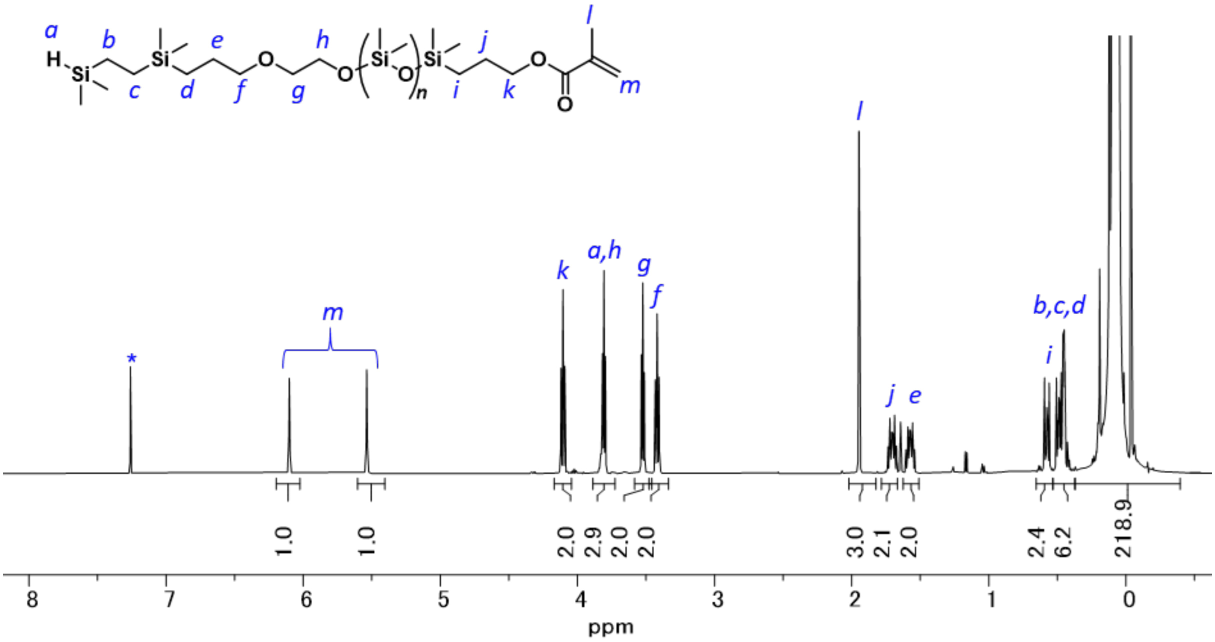

Figure S4. ^{1}H-NMR of H–PDMS–MA in deuterated chloroform.

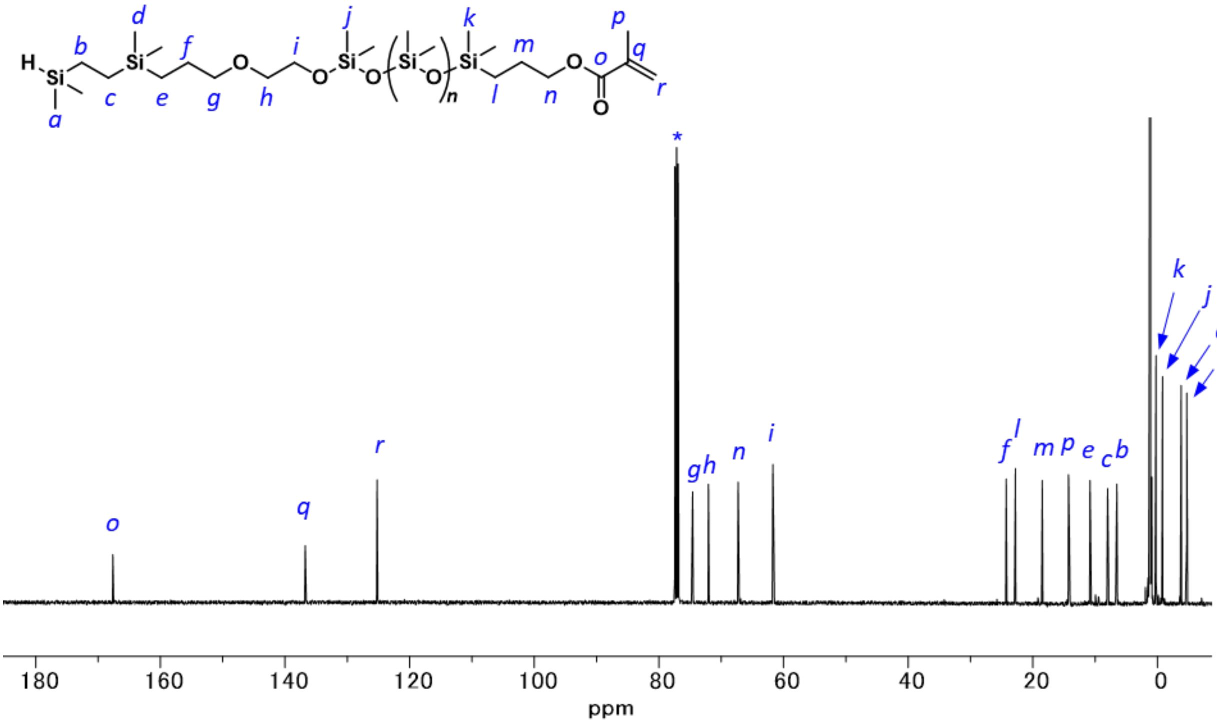

Figure S5. ^{13}C-NMR of H–PDMS–MA in deuterated chloroform.

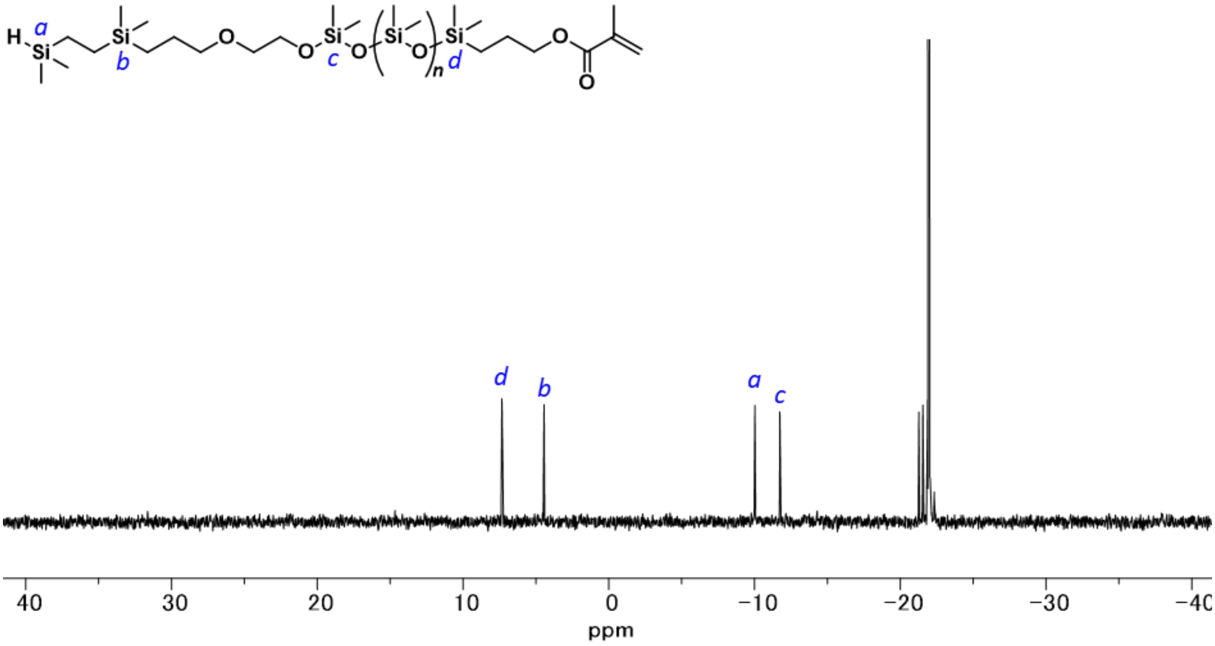

Figure S6. ^{29}Si-NMR of H–PDMS–MA in deuterated chloroform.

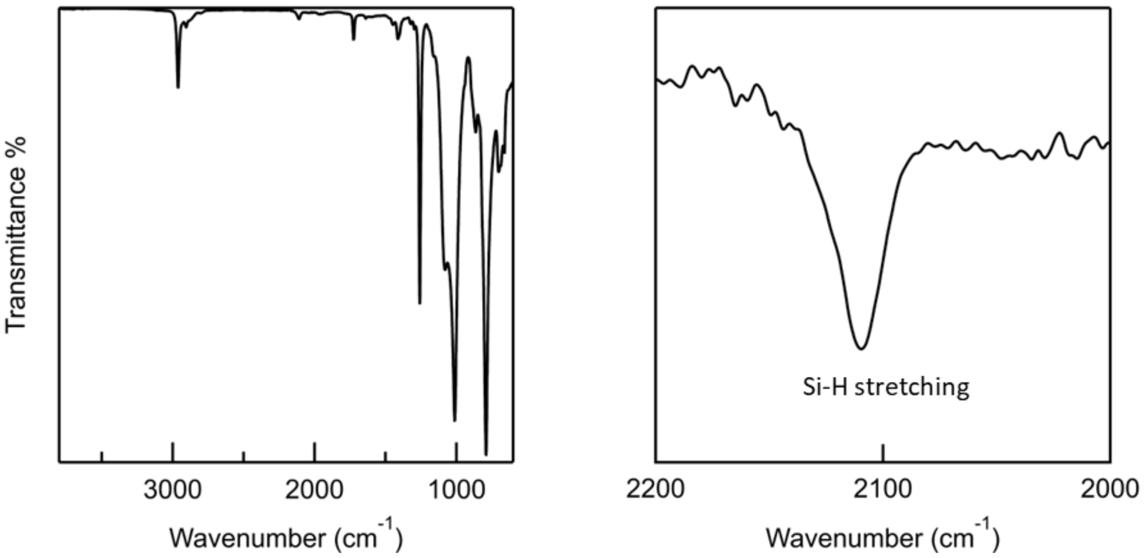

Figure S7. FT-IR of H–PDMS–MA. Si–H stretching peak was observed around 2100 cm^{-1}.

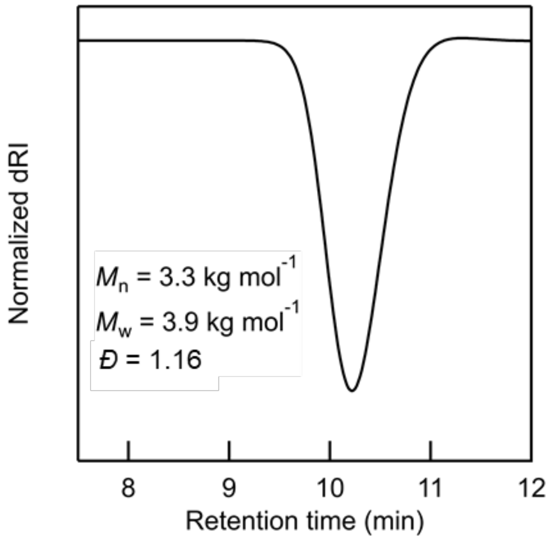

Figure S8. SEC of H–PDMS–MA.

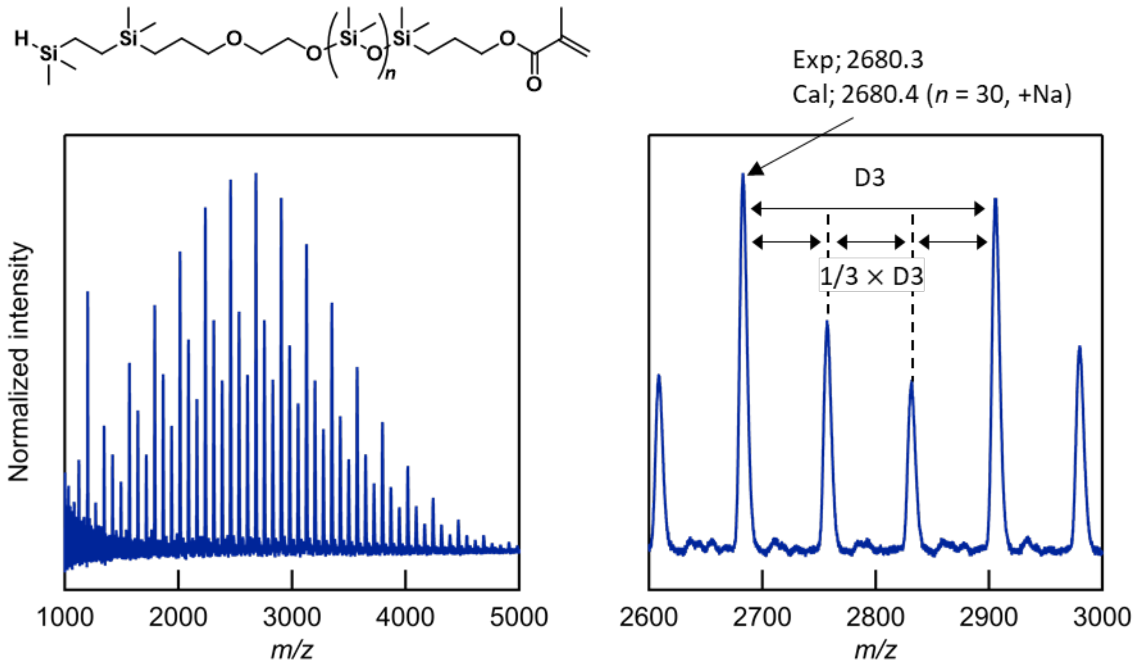

Figure S9. MALDI of H–PDMS–MA

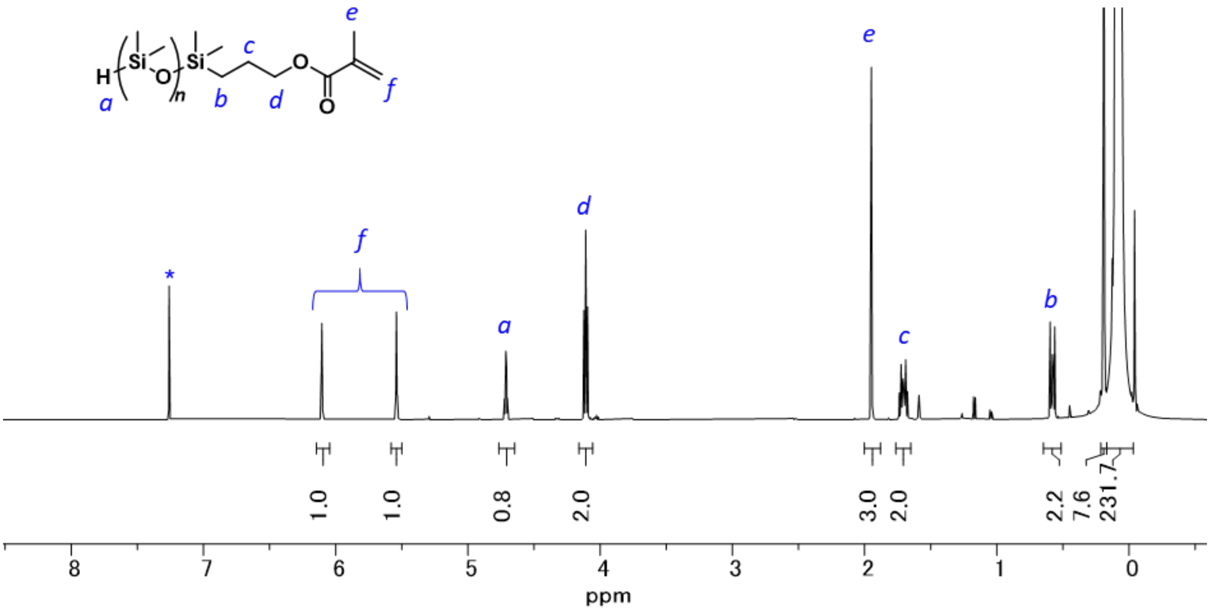

Figure S10. ^{1}H-NMR of H–PDMS–MA polymerized from H–Si–O initiator in deuterated chloroform.

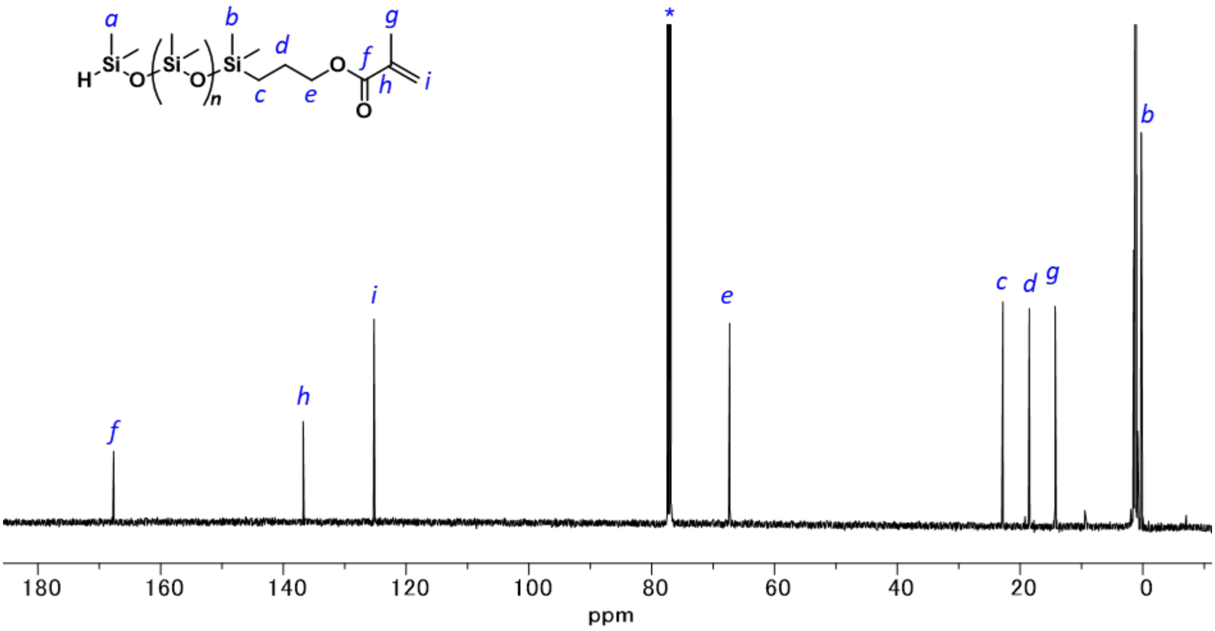

Figure S11. ^{13}C-NMR of H–PDMS–MA polymerized from H–Si–O initiator in deuterated chloroform.

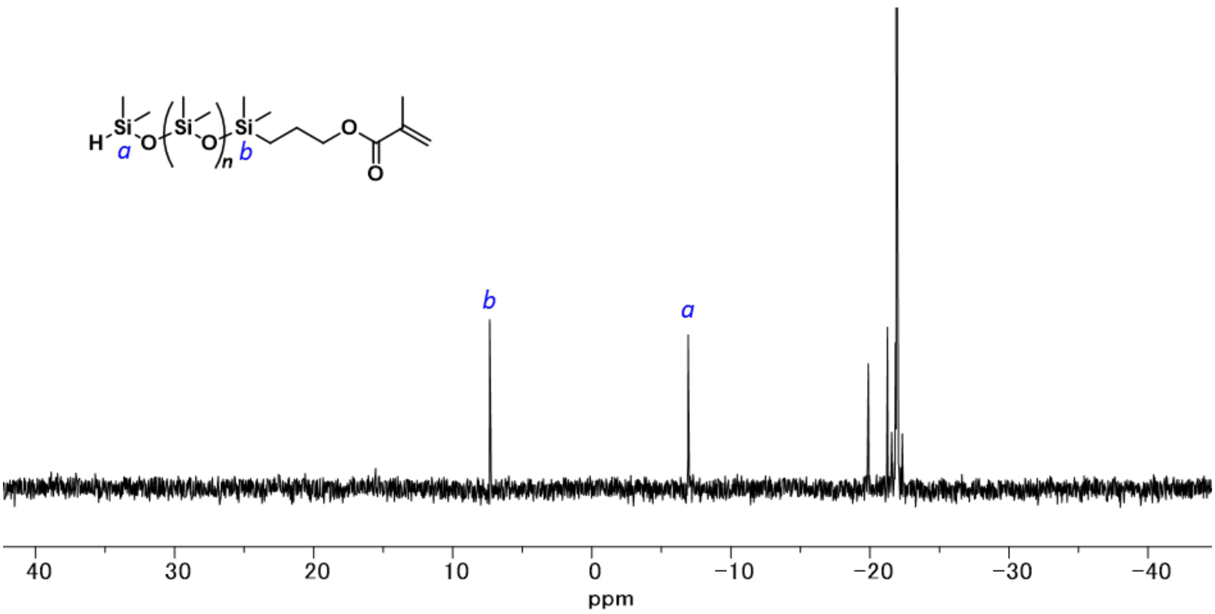

Figure S12. ^{29}Si-NMR of H–PDMS–MA polymerized from H–Si–O initiator in deuterated chloroform.

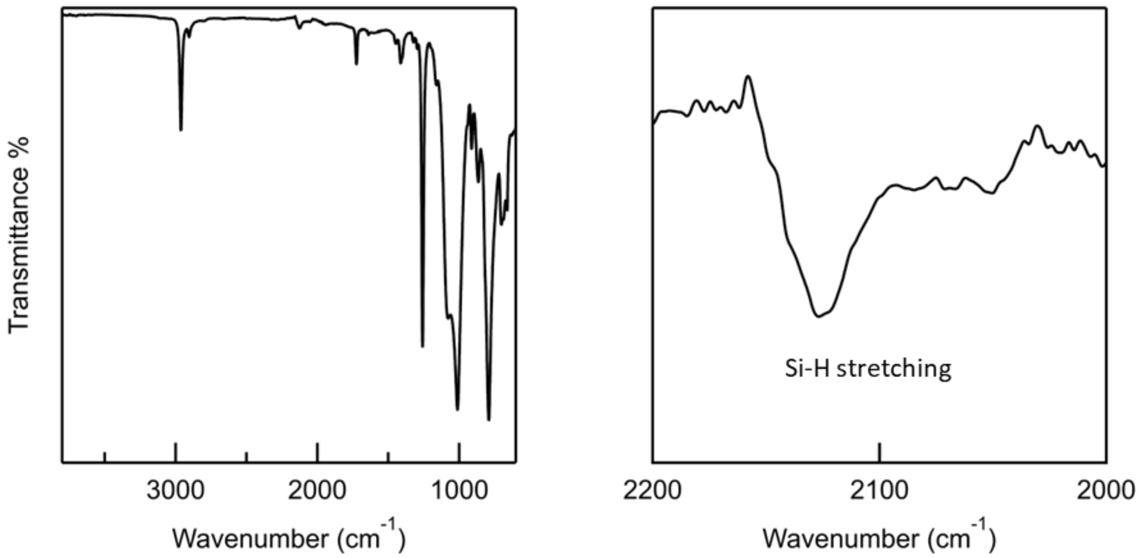

Figure S13. FT-IR of H–PDMS–MA polymerized from H–Si–O initiator. Si–H stretching peak was observed around 2100 cm^{-1}.

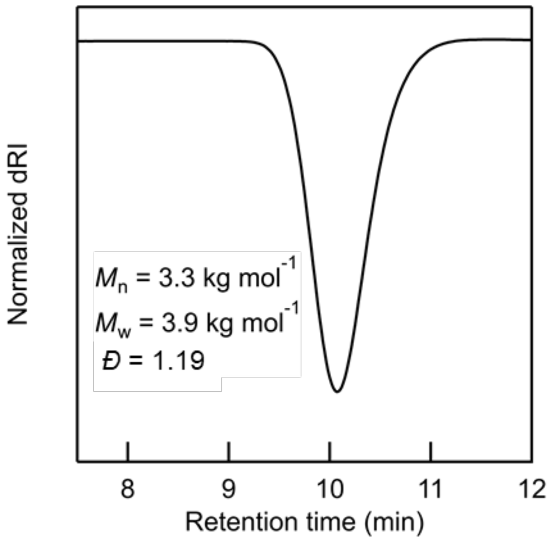

Figure S14. SEC of H–PDMS–MA polymerized from H–Si–O initiator.

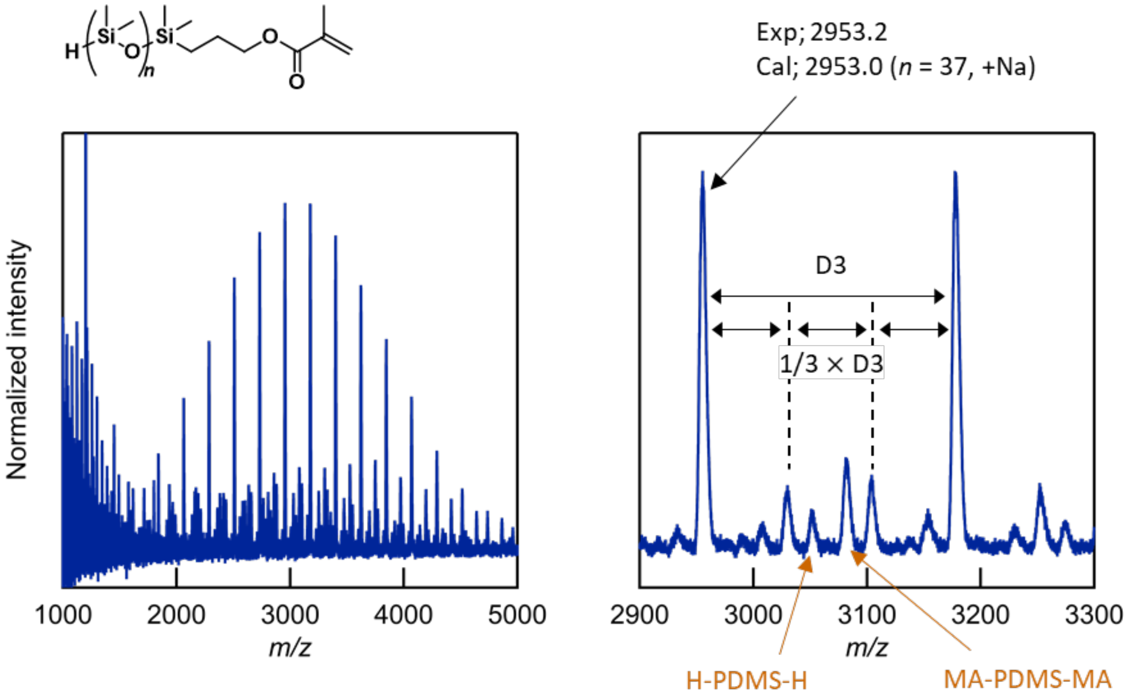

Figure S15. MALDI of H–PDMS–MA polymerized from H–Si–O initiator.

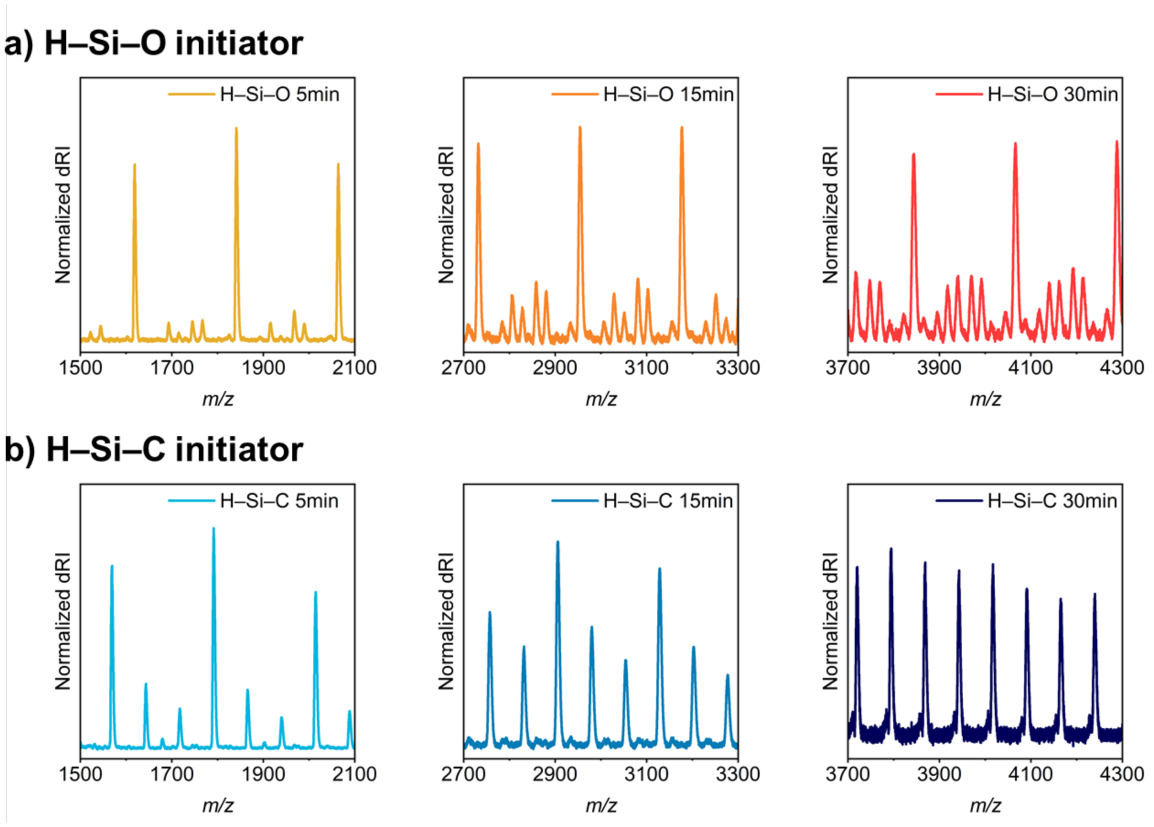

Figure S16. (a) MALDI of H–PDMS–MA synthesized with H–Si–O initiator and (b) H–Si–O initiator for 5, 15, and 30 min, respectively.

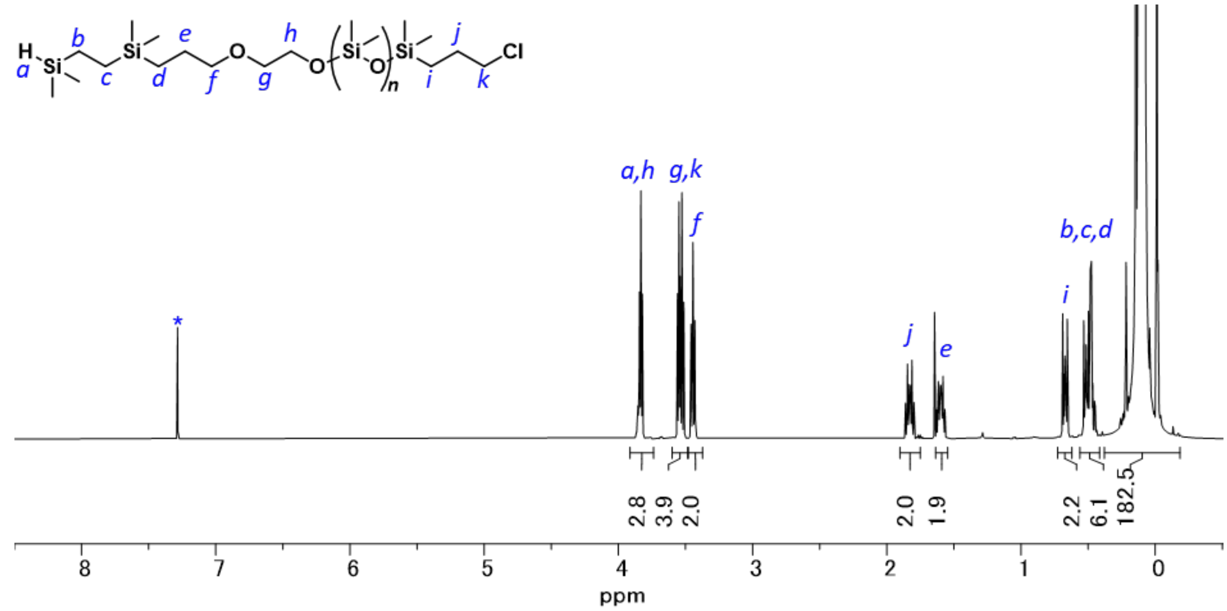

Figure S17. ^{1}H-NMR of H–PDMS–Cl in deuterated chloroform.

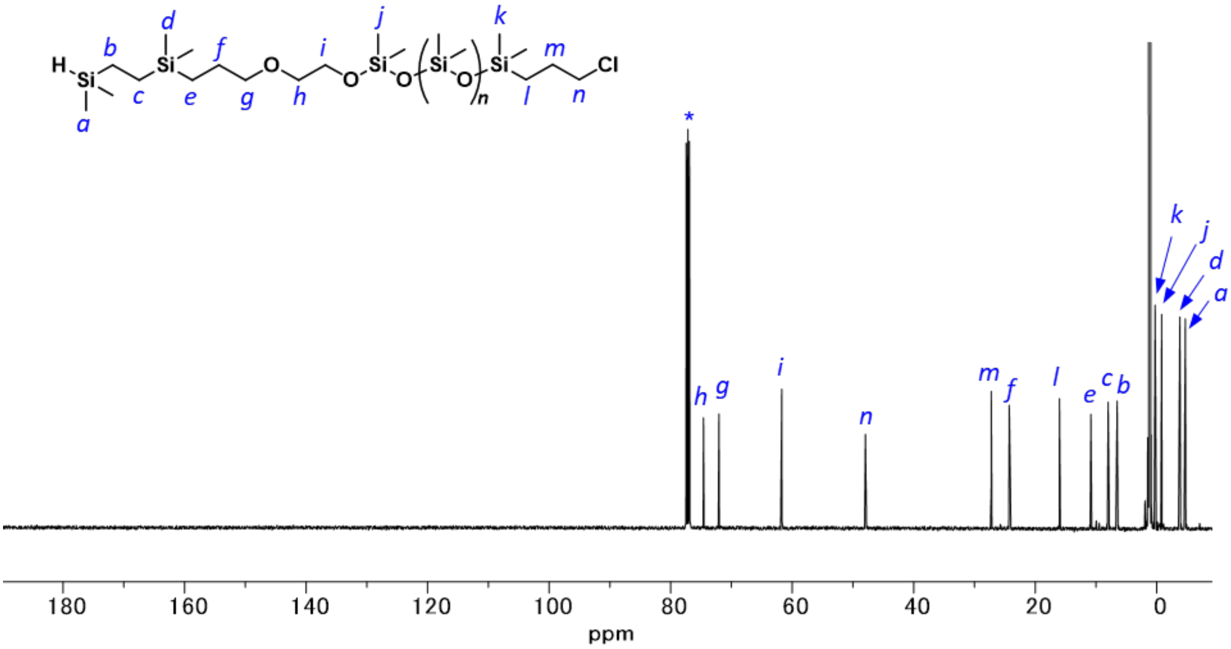

Figure S18. ^{13}C-NMR of H–PDMS–Cl in deuterated chloroform.

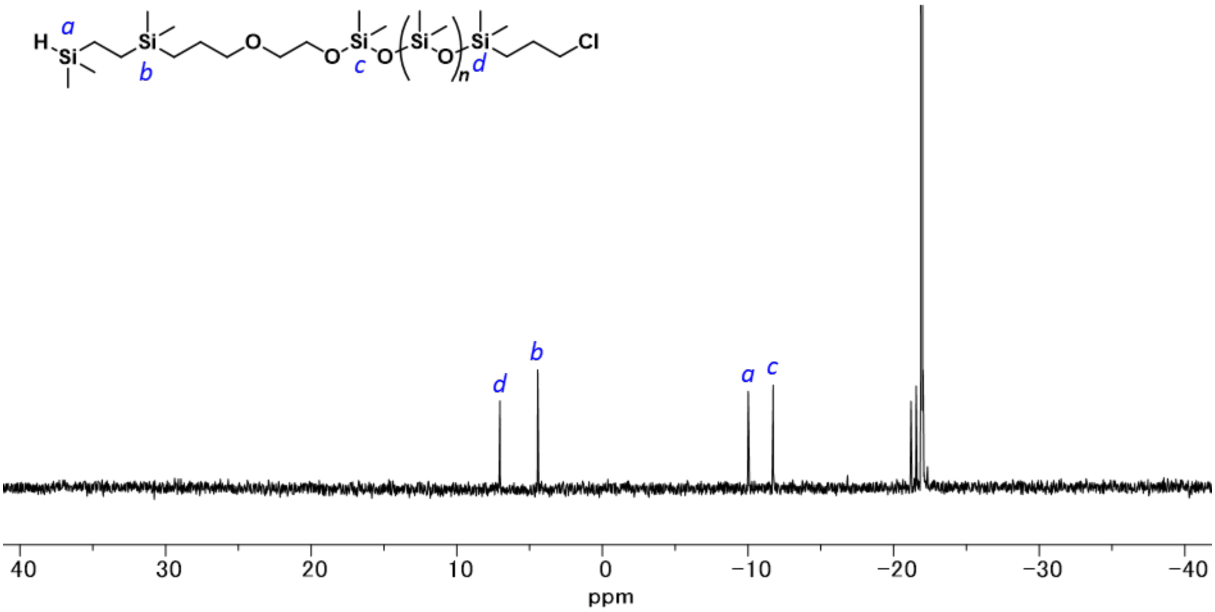

Figure S19. ^{29}Si-NMR of H–PDMS–Cl in deuterated chloroform.

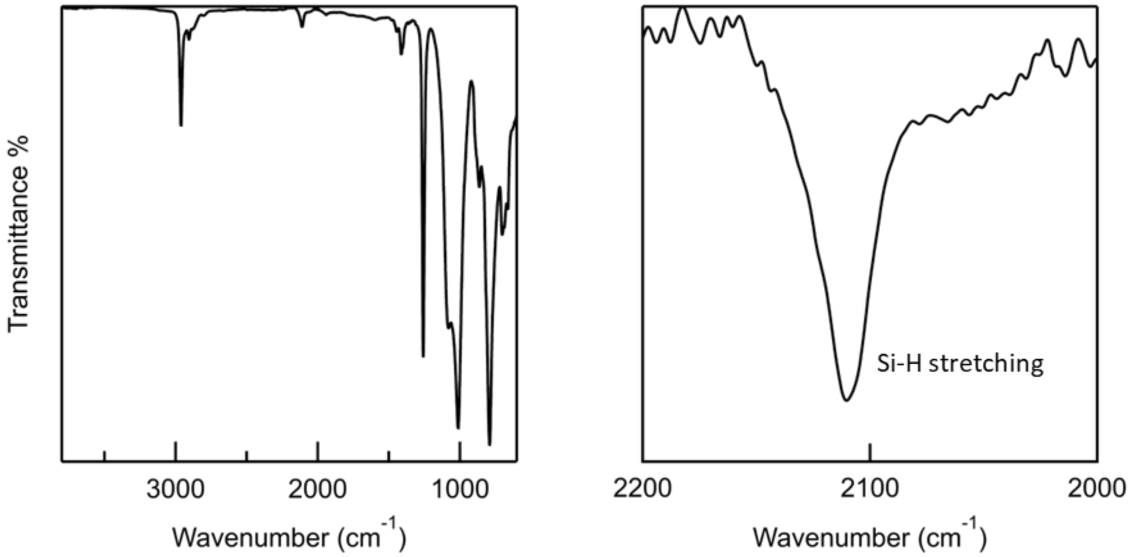

Figure S20. FT-IR of H–PDMS–Cl. Si–H stretching peak was observed around 2100 cm^{-1}.

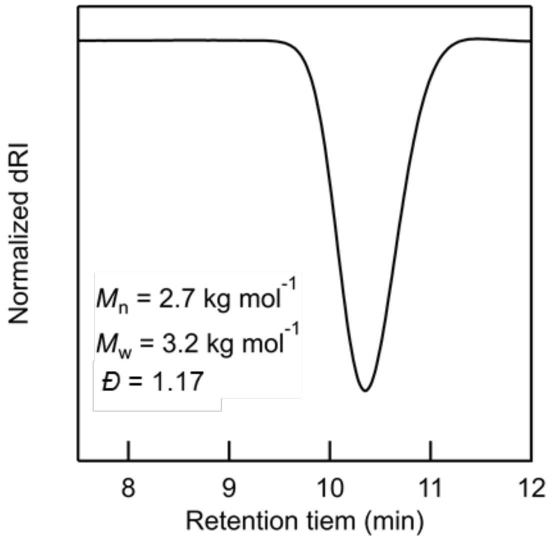

Figure S21. SEC of H–PDMS–Cl.

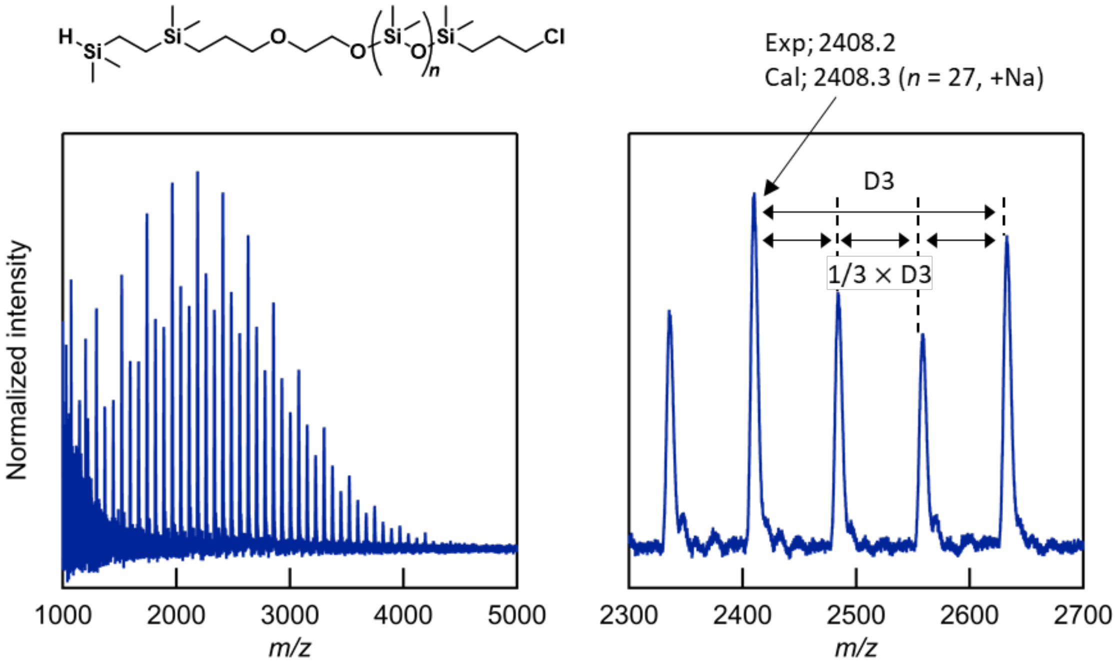

Figure S22. MALDI of H–PDMS–Cl.

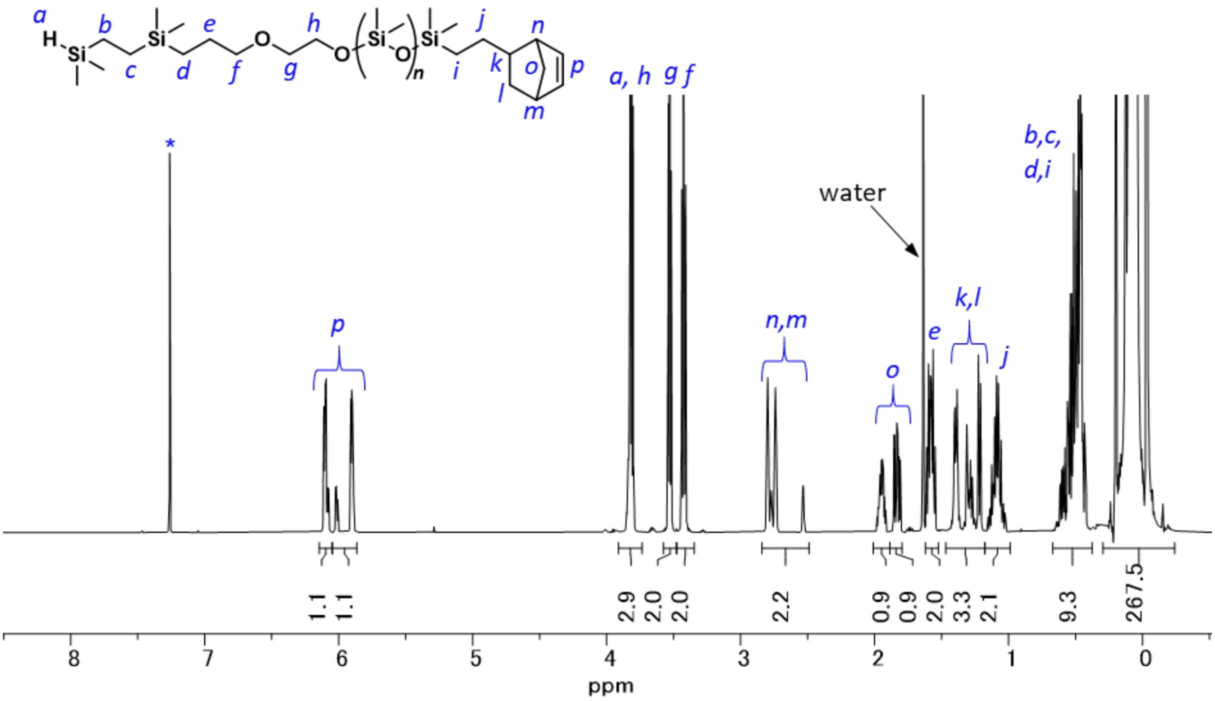

Figure S23. ^{1}H-NMR of H–PDMS–Nb in deuterated chloroform.

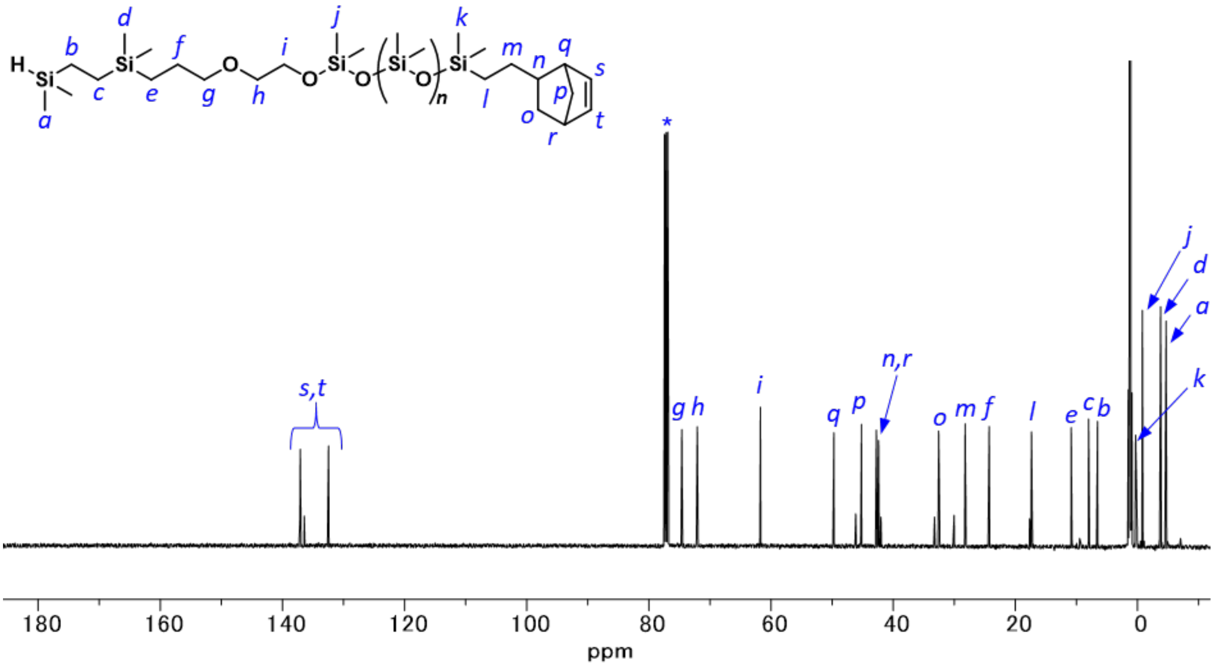

Figure S24. ^{13}C-NMR of H–PDMS–Nb in deuterated chloroform.

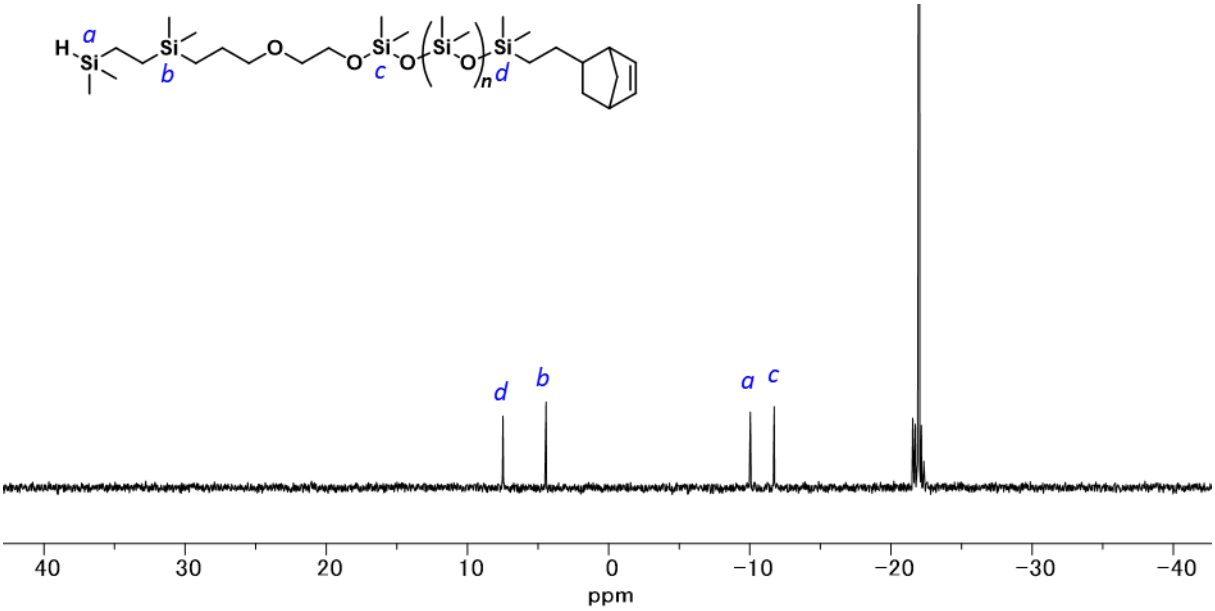

Figure S25. ^{29}Si-NMR of H–PDMS–Nb in deuterated chloroform.

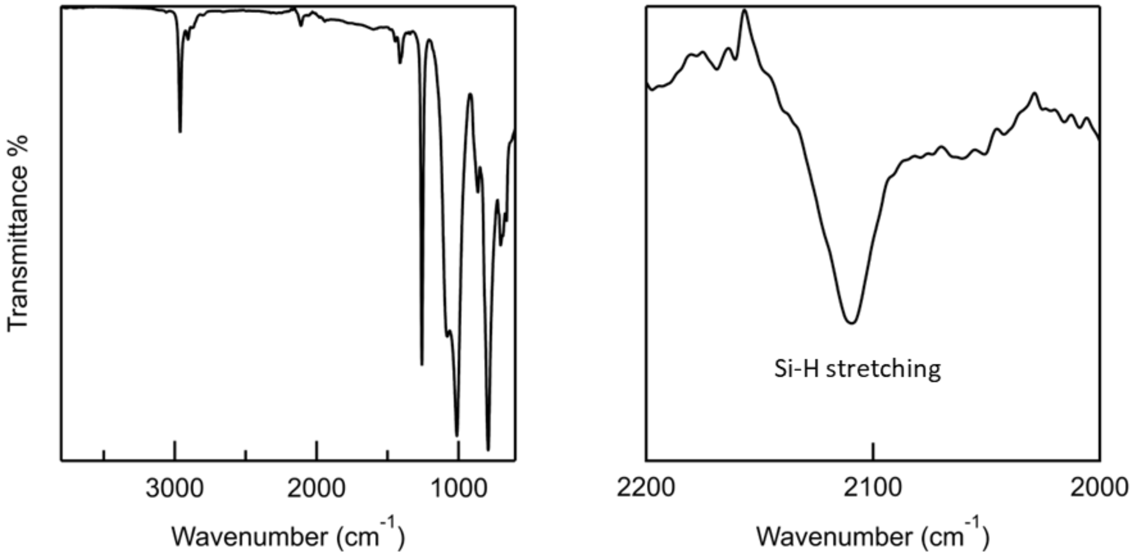

Figure S26. FT-IR of H–PDMS–Nb. Si–H stretching peak was observed around 2100 cm^{-1}.

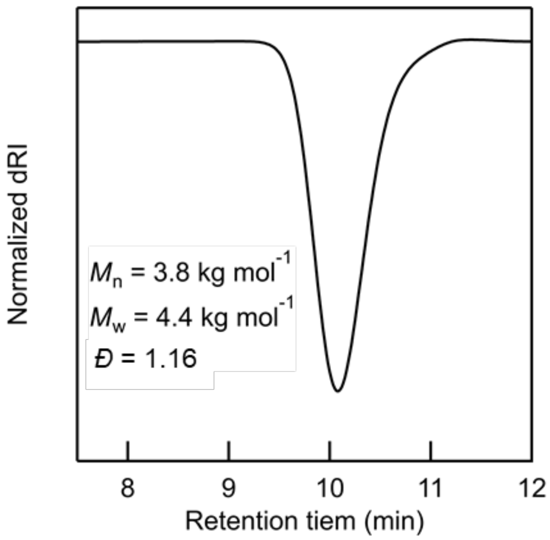

Figure S27. SEC of H–PDMS–Nb.

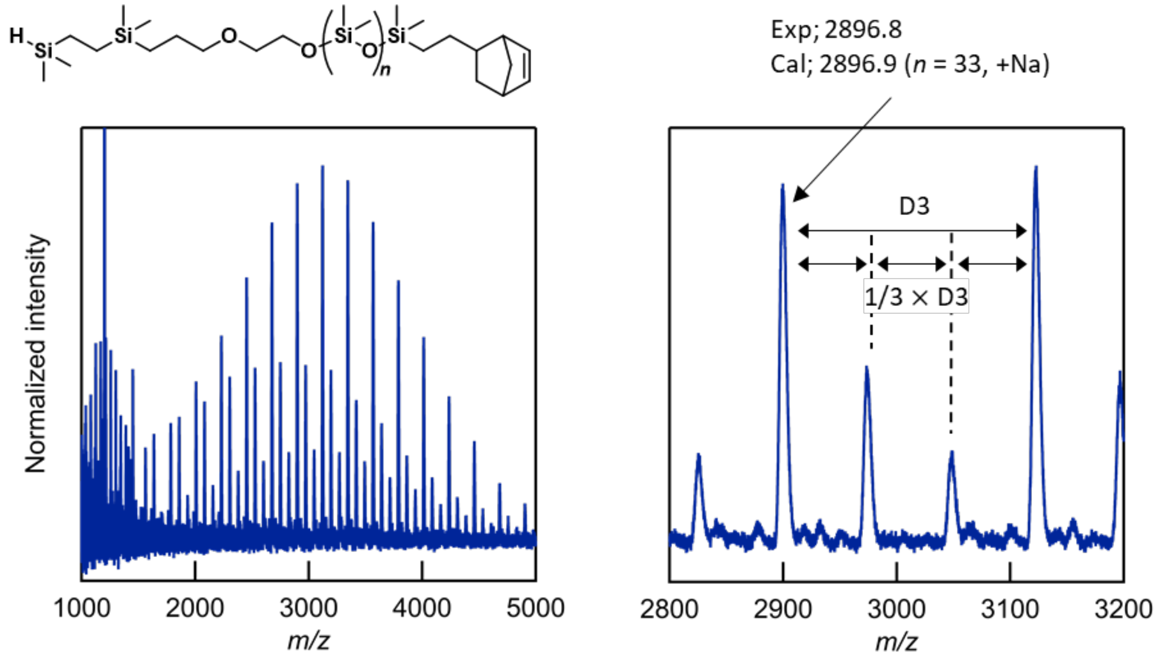

Figure S28. MALDI of H–PDMS–Nb.

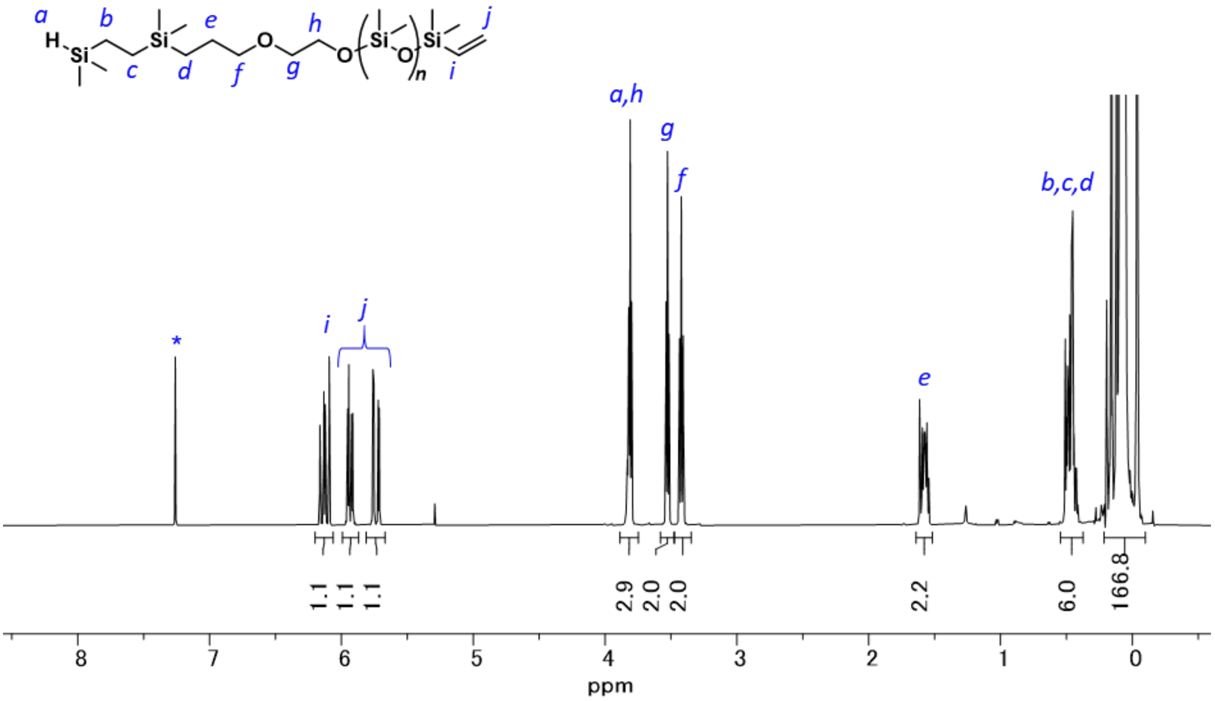

Figure S29. ^{1}H-NMR of H–PDMS–Vinyl in deuterated chloroform.

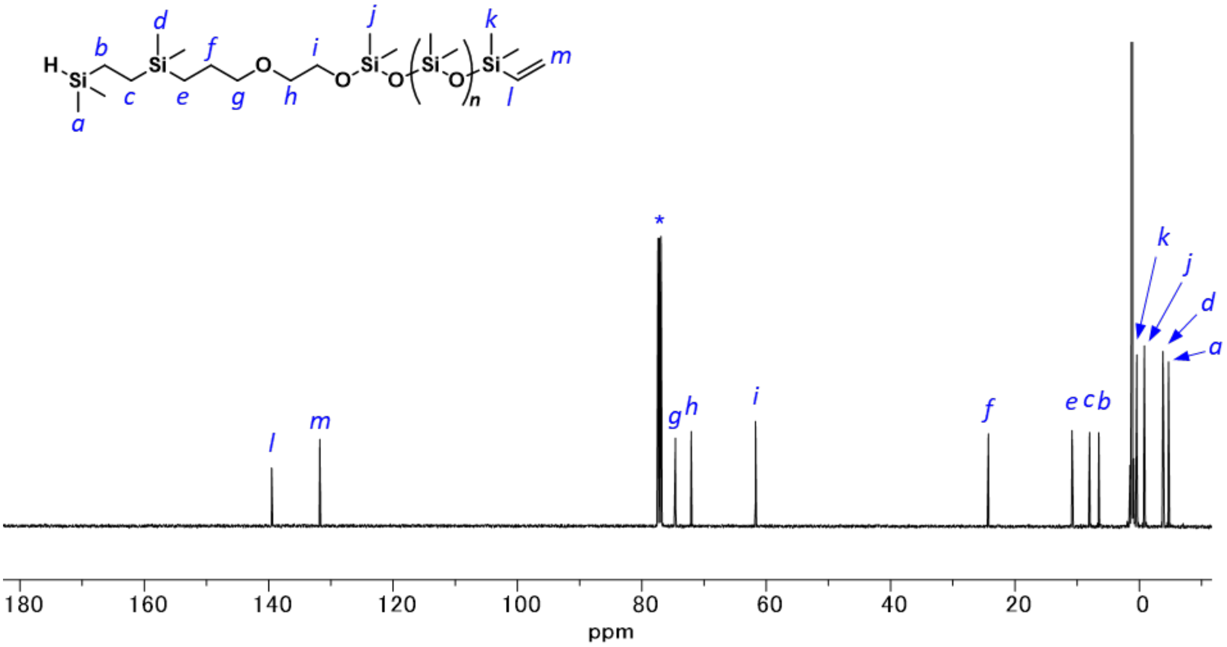

Figure S30. ^{13}C-NMR of H–PDMS–Vinyl in deuterated chloroform.

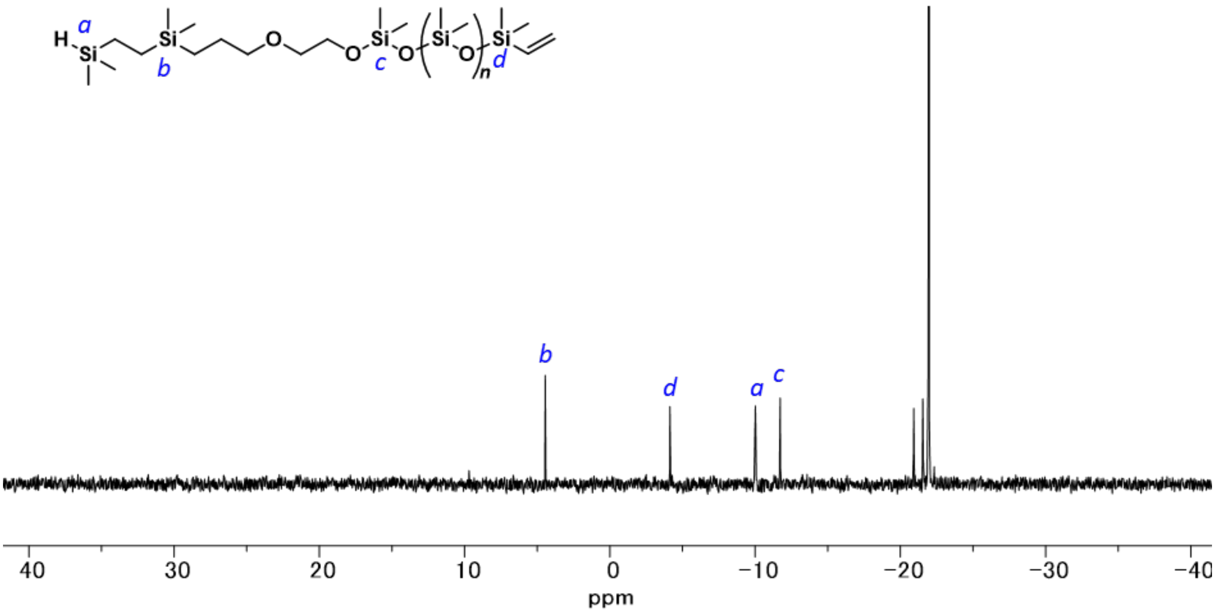

Figure S31. ^{29}Si-NMR of H–PDMS–Vinyl in deuterated chloroform.

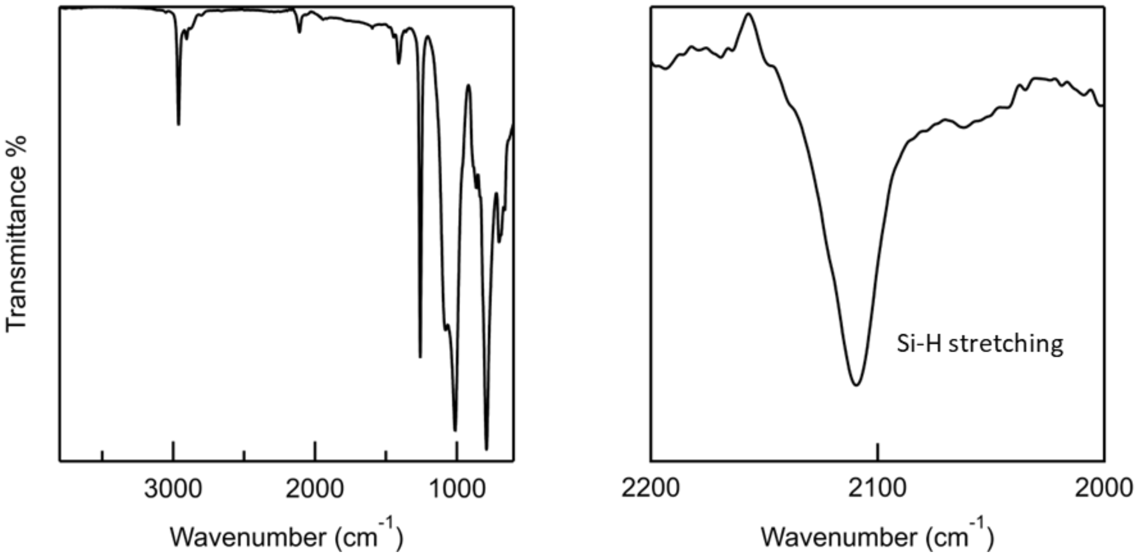

Figure S32. FT-IR of H–PDMS–Vinyl. Si–H stretching peak was observed around 2100 cm^{-1}.

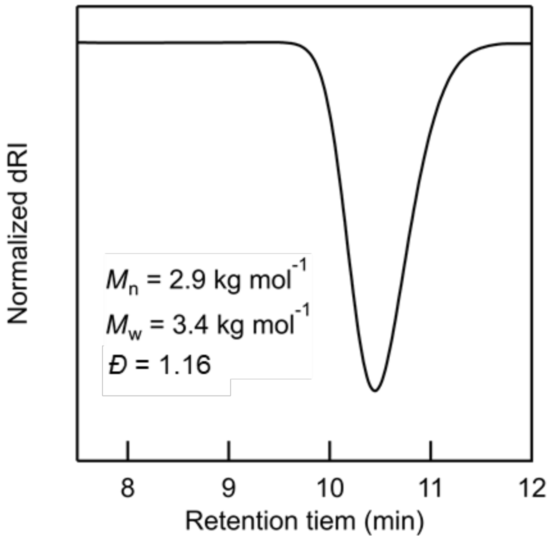

Figure S33. SEC of H–PDMS–Vinyl.

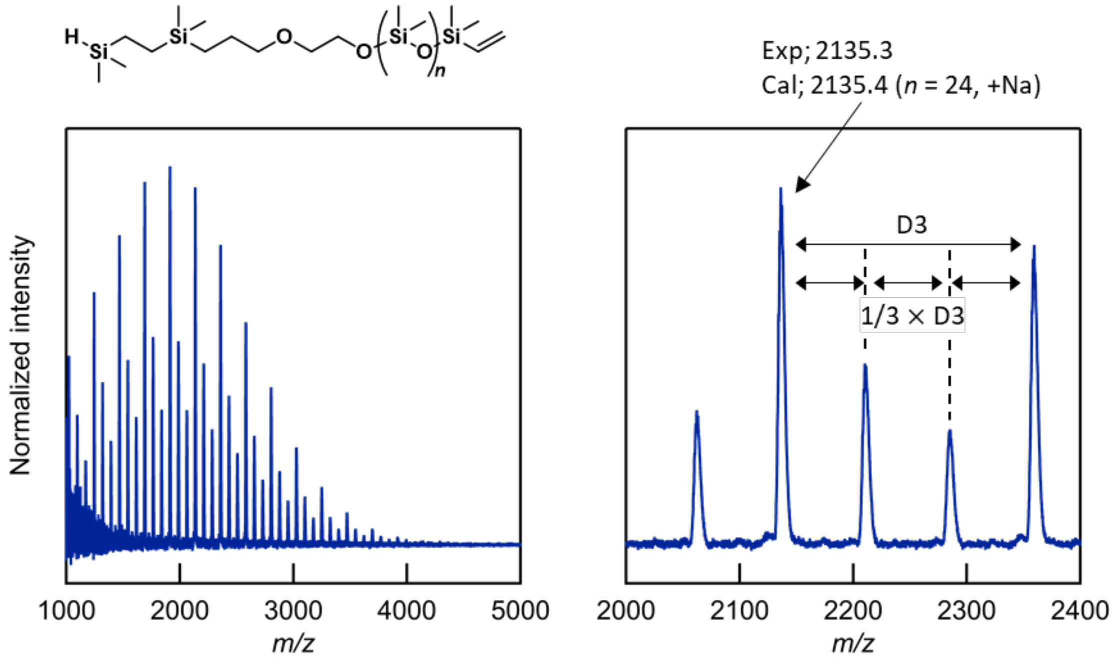

Figure S34. MALDI of H–PDMS–Vinyl.

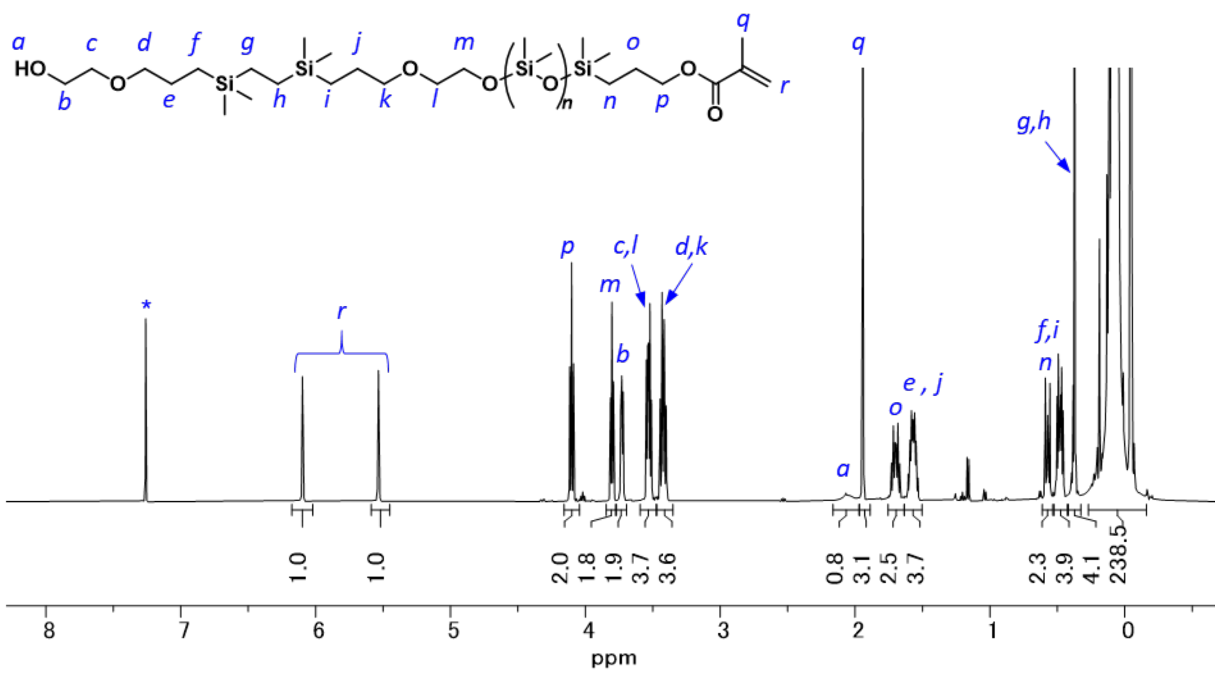

Figure S35. ^{1}H-NMR of HO–PDMS–MA in deuterated chloroform.

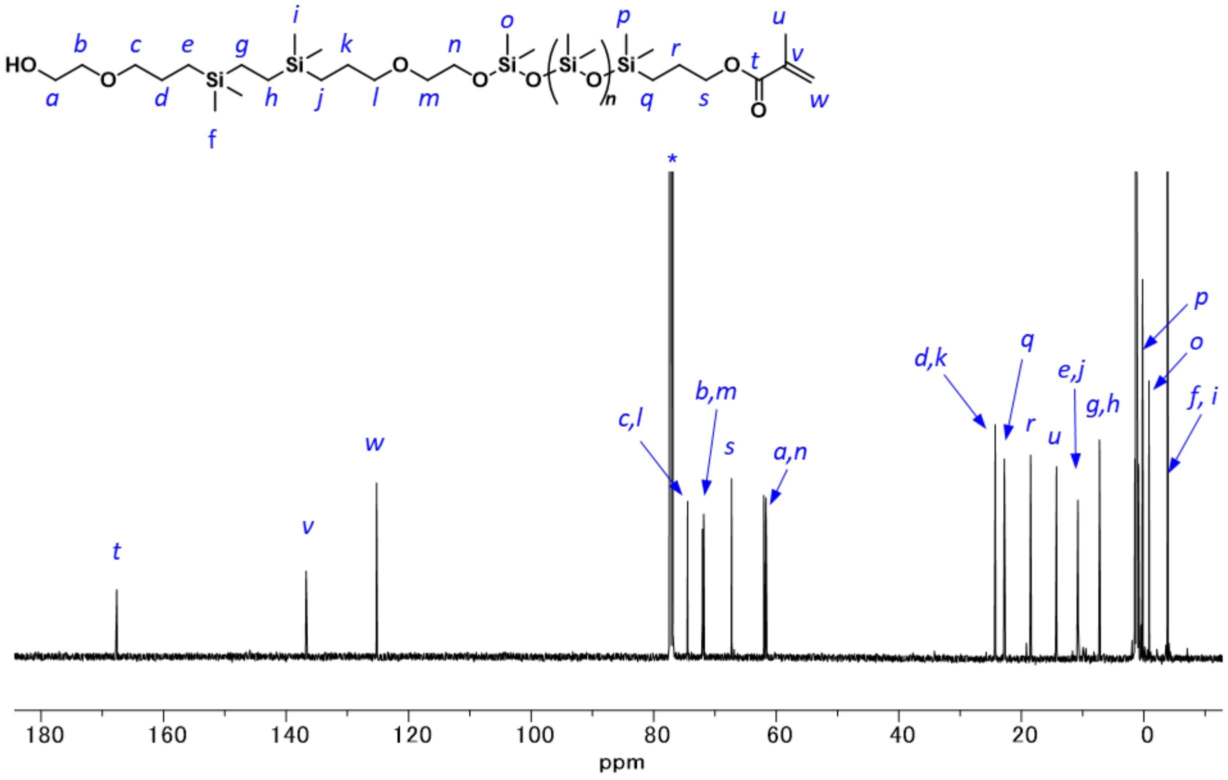

Figure S36. ^{13}C-NMR of HO–PDMS–MA in deuterated chloroform.

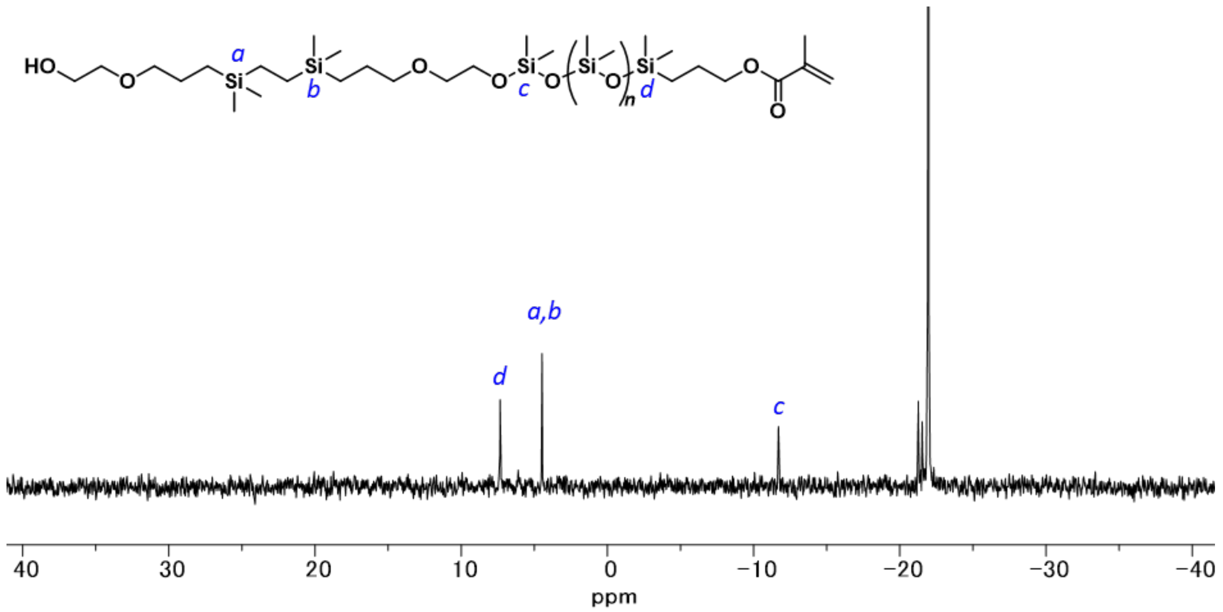

Figure S37. ^{29}Si-NMR of HO–PDMS–MA in deuterated chloroform.

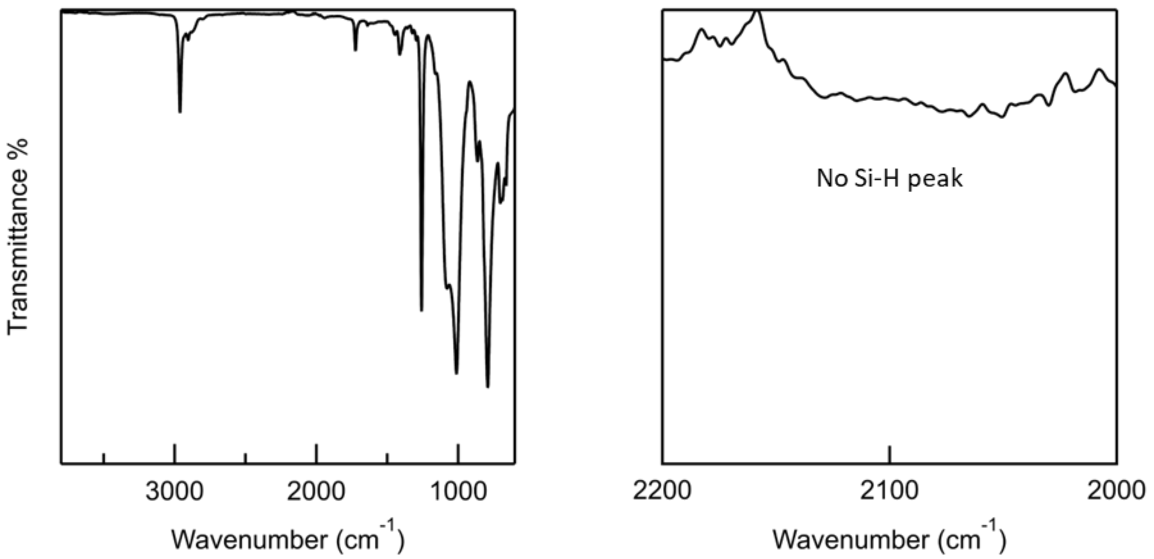

Figure S38. FT-IR of HO–PDMS–MA. Si–H stretching peak was not observed after hydrosilylation.

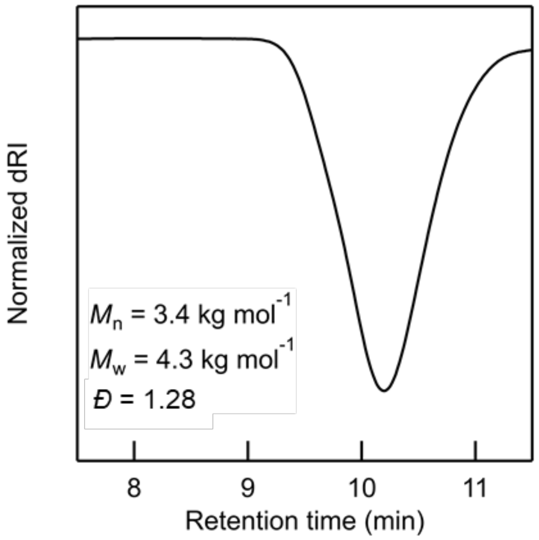

Figure S39. SEC of HO–PDMS–MA.

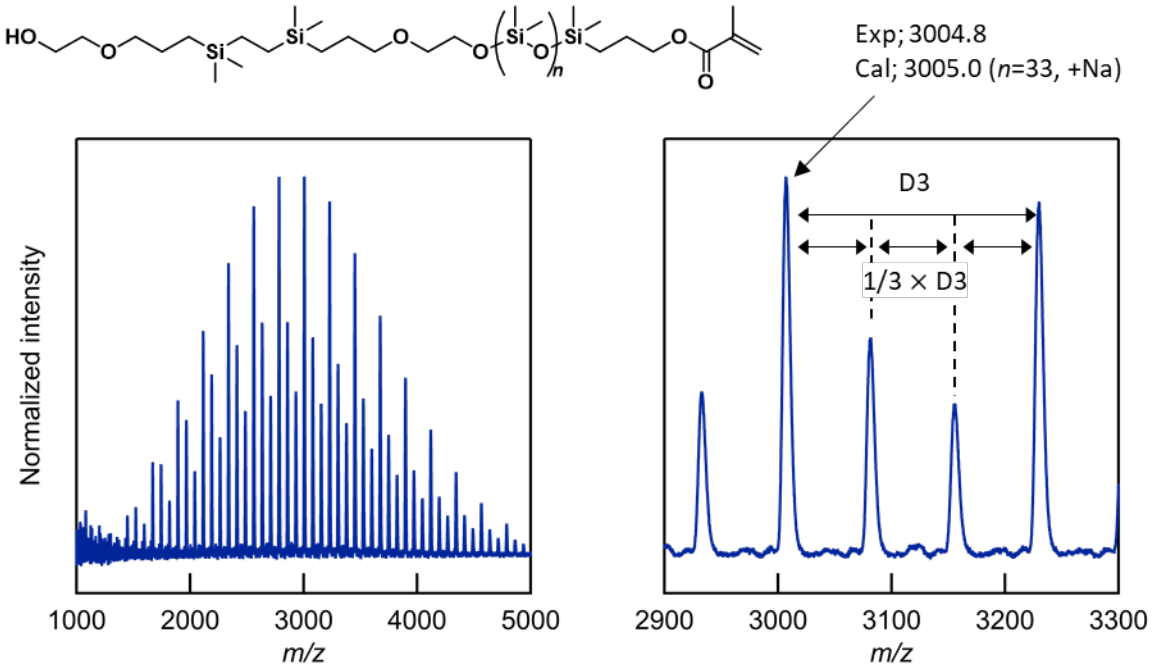

Figure S40. MALDI of HO–PDMS–MA.

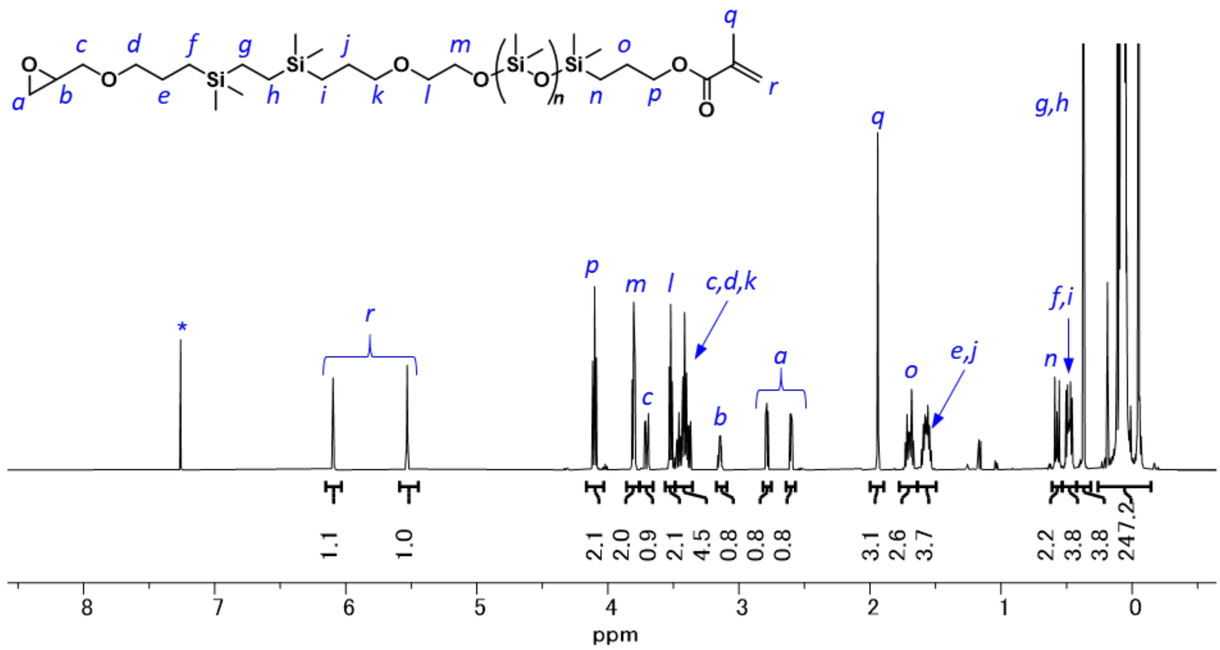

Figure S41. ^{1}H-NMR of Epoxy–PDMS–MA in deuterated chloroform.

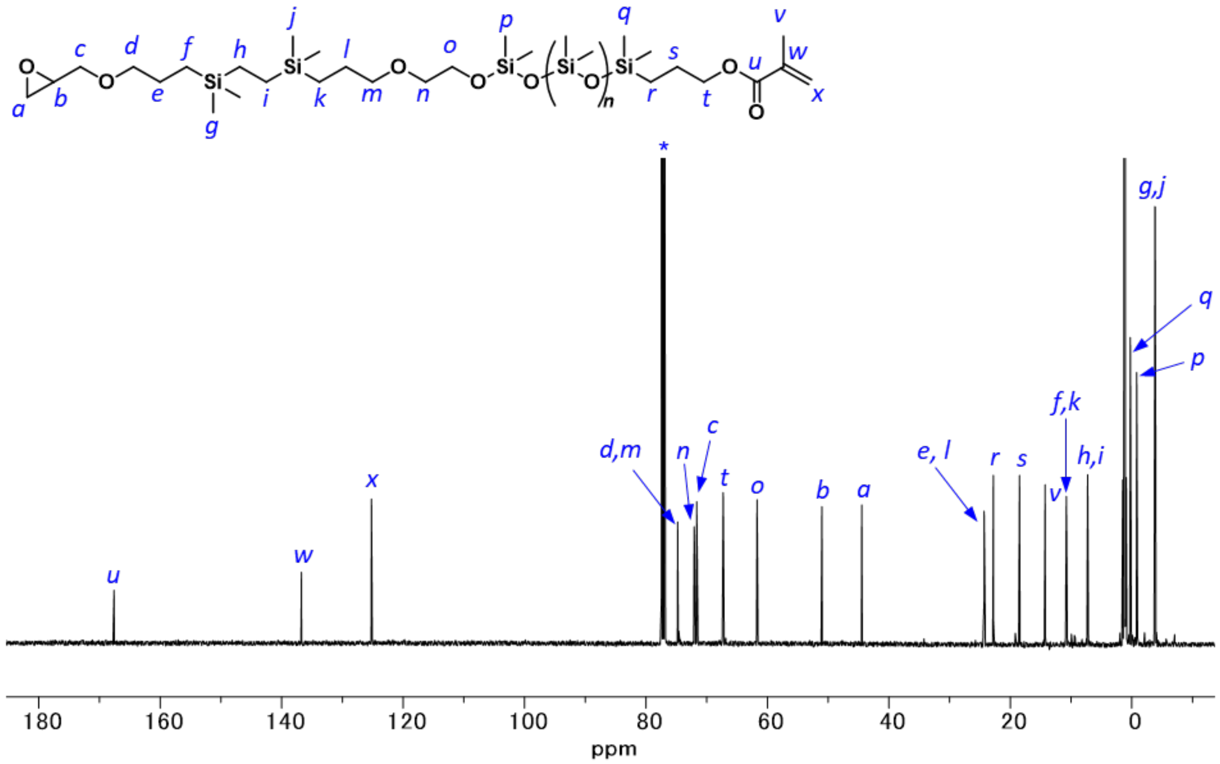

Figure S42. ^{13}C-NMR of Epoxy–PDMS–MA in deuterated chloroform.

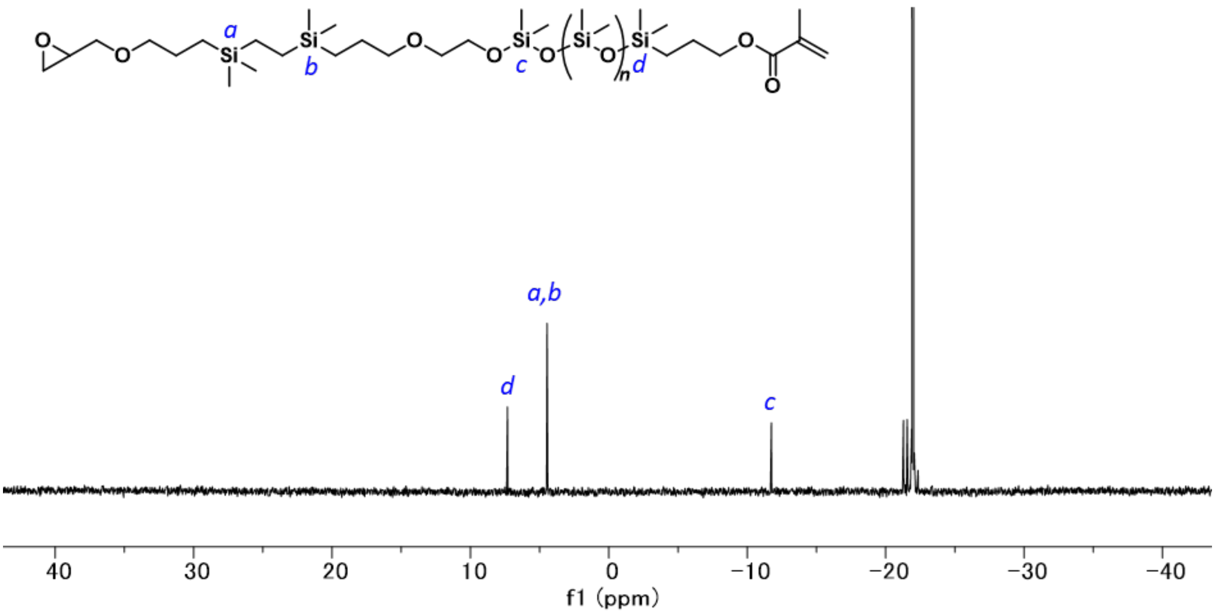

Figure S43. ^{29}Si-NMR of Epoxy–PDMS–MA in deuterated chloroform.

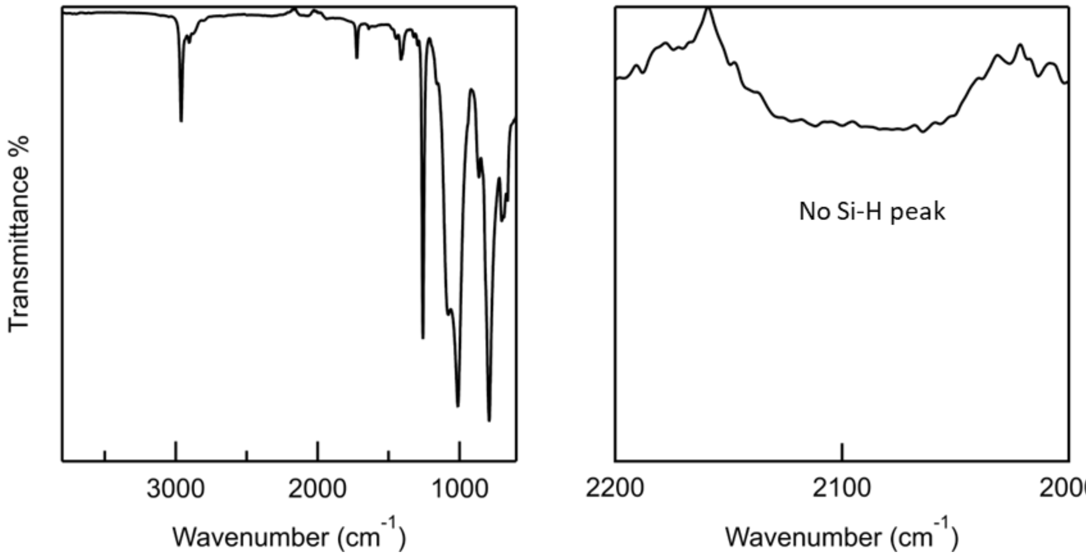

Figure S44. FT-IR of Epoxy–PDMS–MA. Si–H stretching peak was not observed after hydrosilylation.

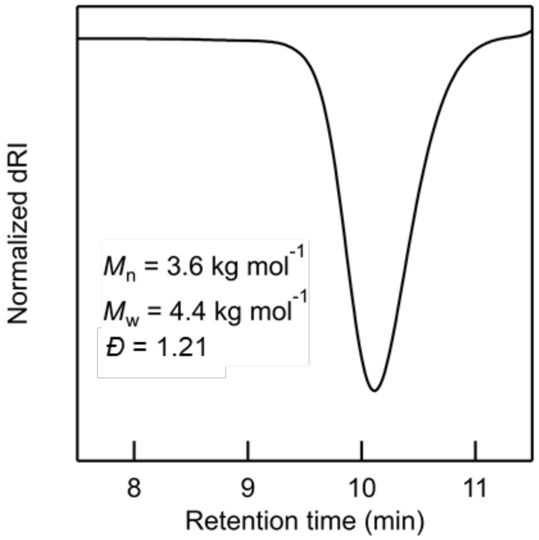

Figure S45. SEC of Epoxy–PDMS–MA.

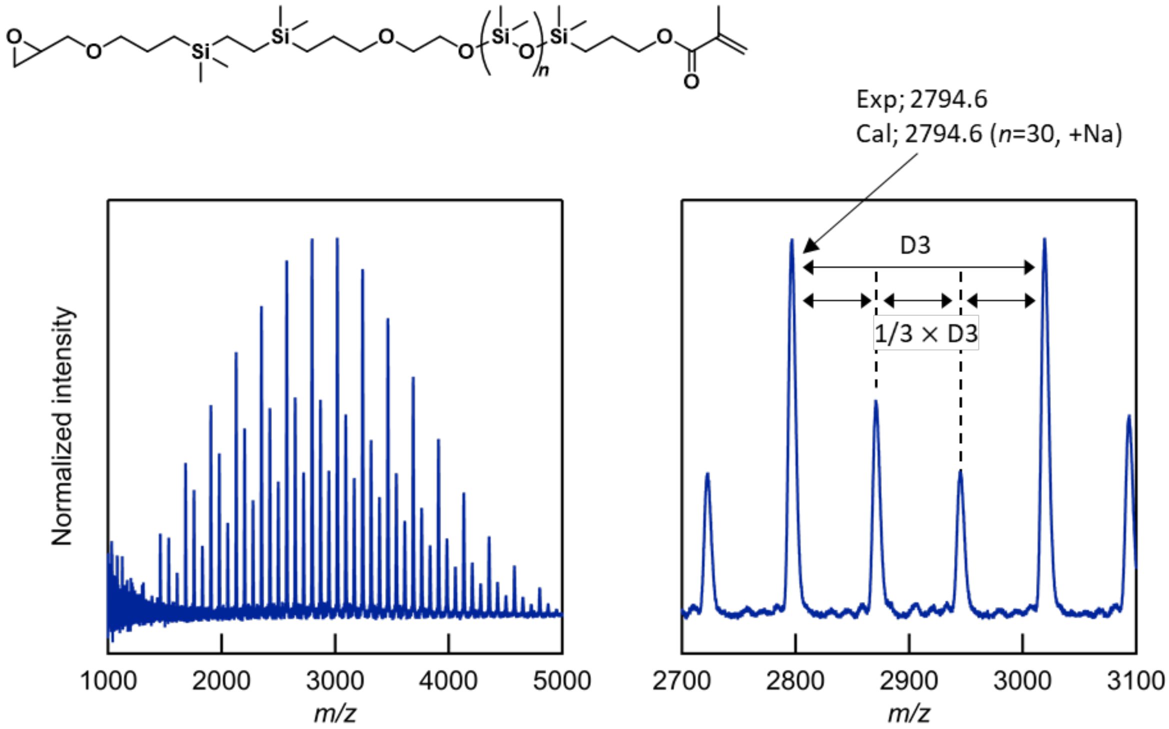

Figure S46. MALDI of Epoxy–PDMS–MA.

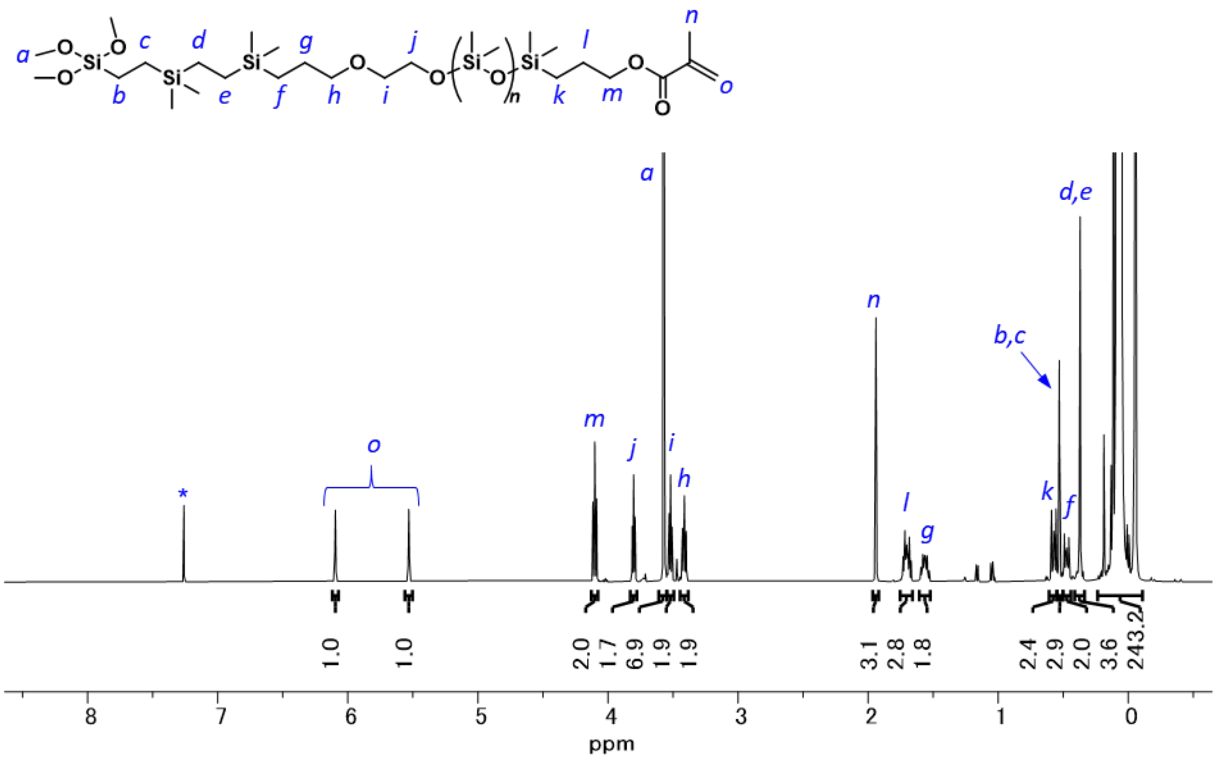

Figure S47. ^{1}H-NMR of $(MeO)_3$Si–PDMS–MA in deuterated chloroform.

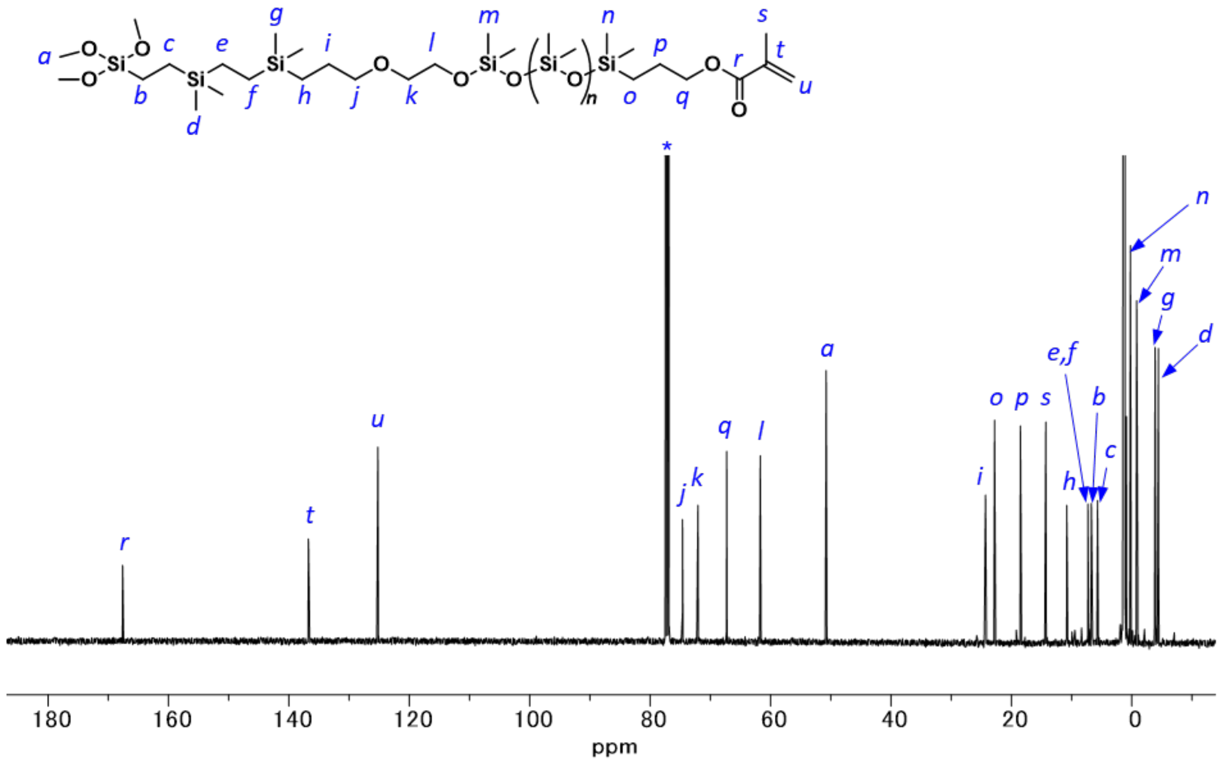

Figure S48 ^{13}C-NMR of $(MeO)_3$Si–PDMS–MA in deuterated chloroform.

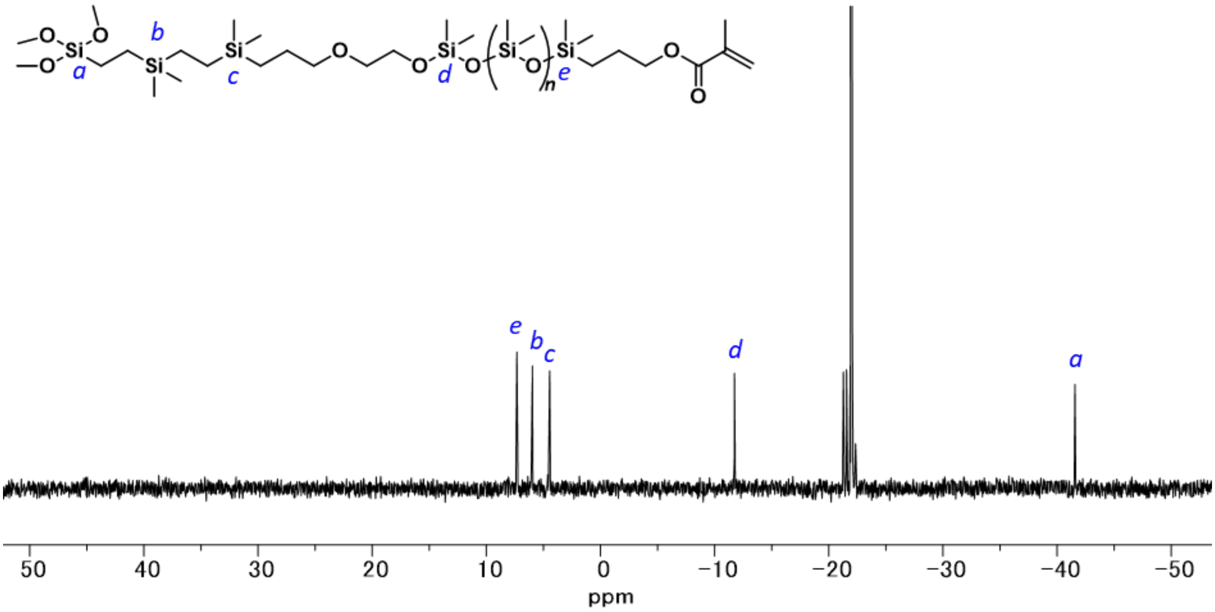

Figure S49. ^{29}Si-NMR of $(MeO)_3Si$–PDMS–MA in deuterated chloroform.

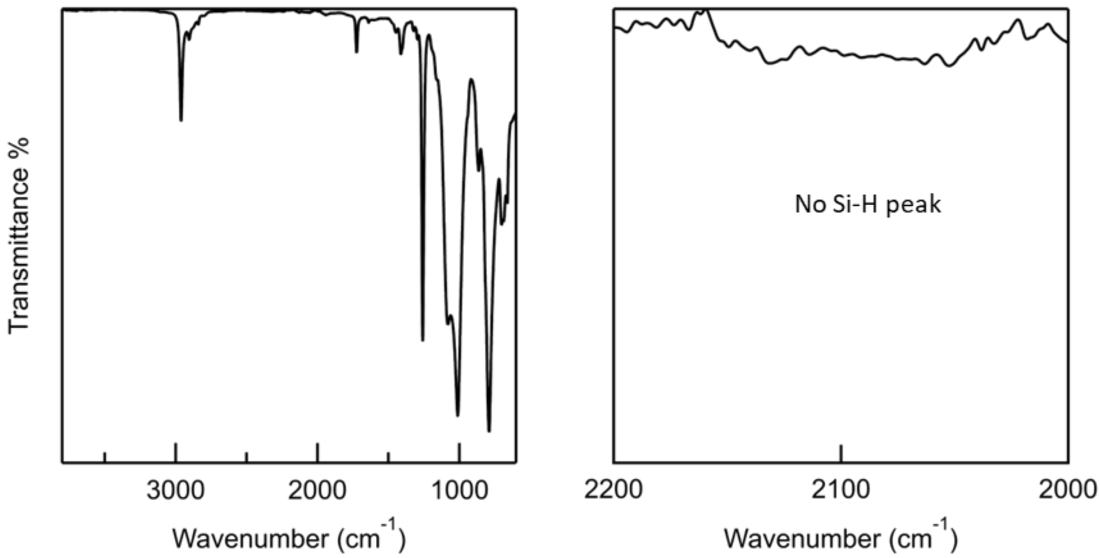

Figure S50. FT-IR of $(MeO)_3Si$–PDMS–MA. Si–H stretching peak was not observed after hydrosilylation.

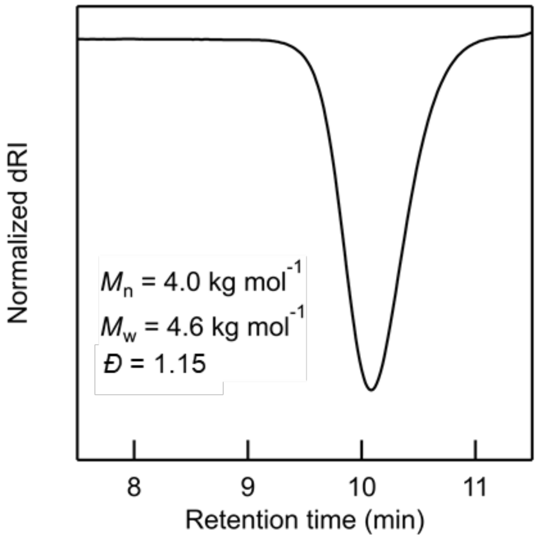

Figure S51. SEC of (MeO)$_3$Si–PDMS–MA.

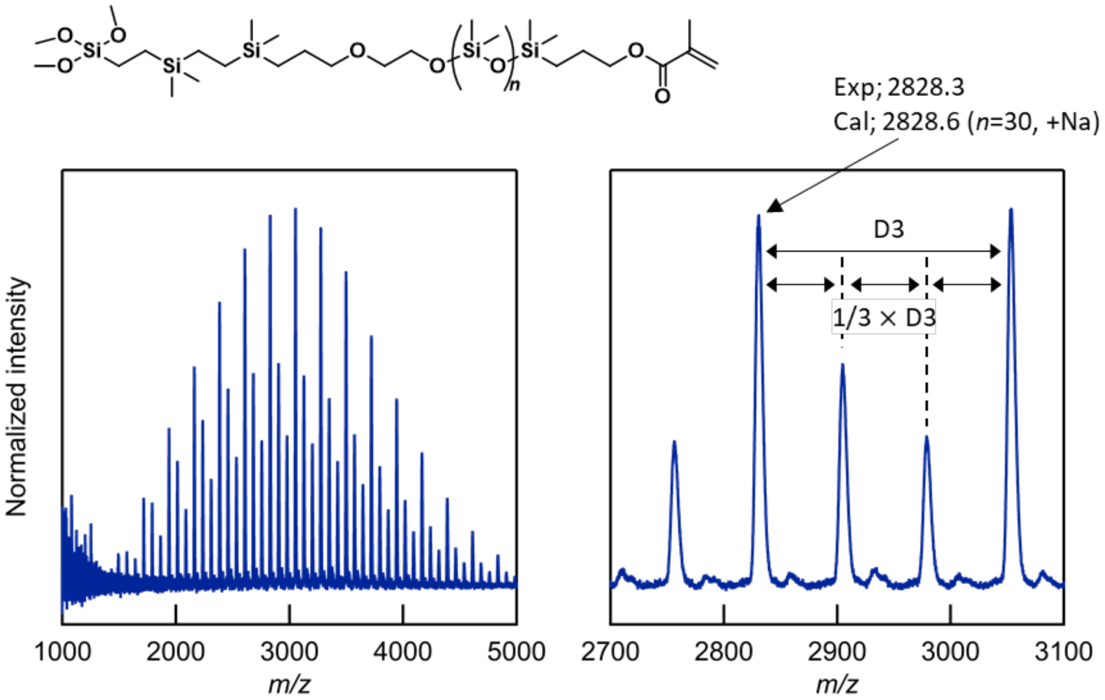

Figure S52. MALDI of (MeO)$_3$Si–PDMS–MA.

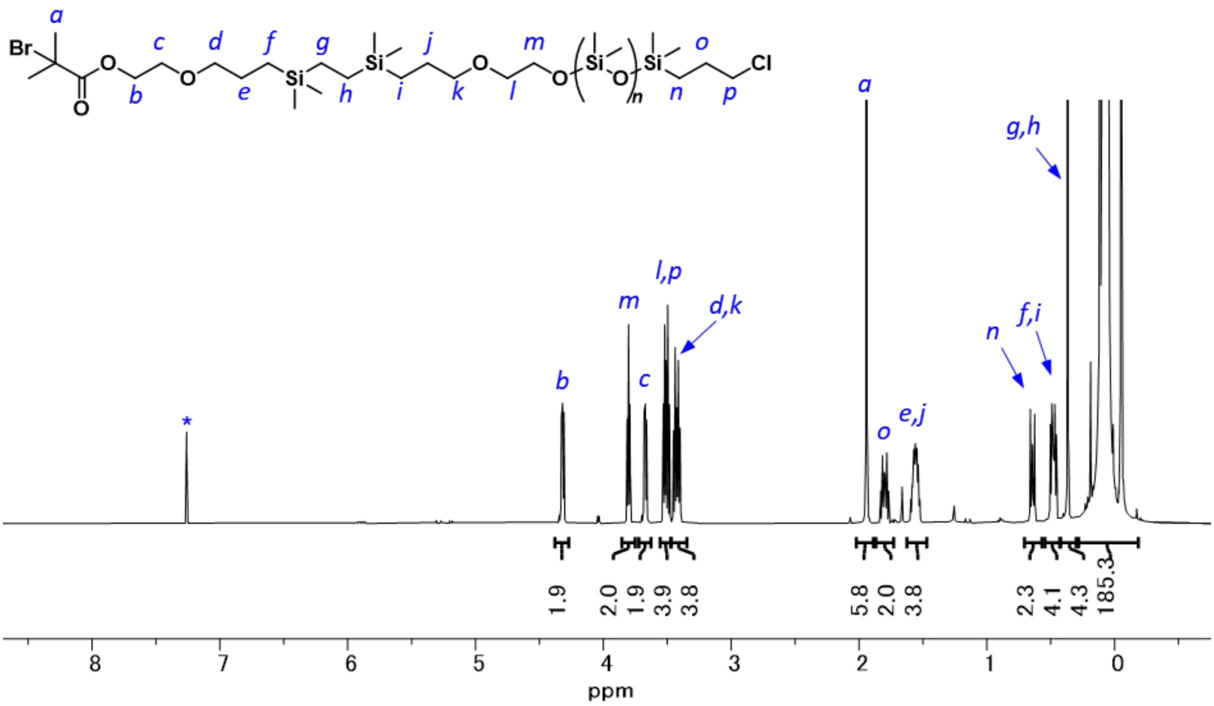

Figure S53. ^{1}H-NMR of ATRP–PDMS–Cl in deuterated chloroform.

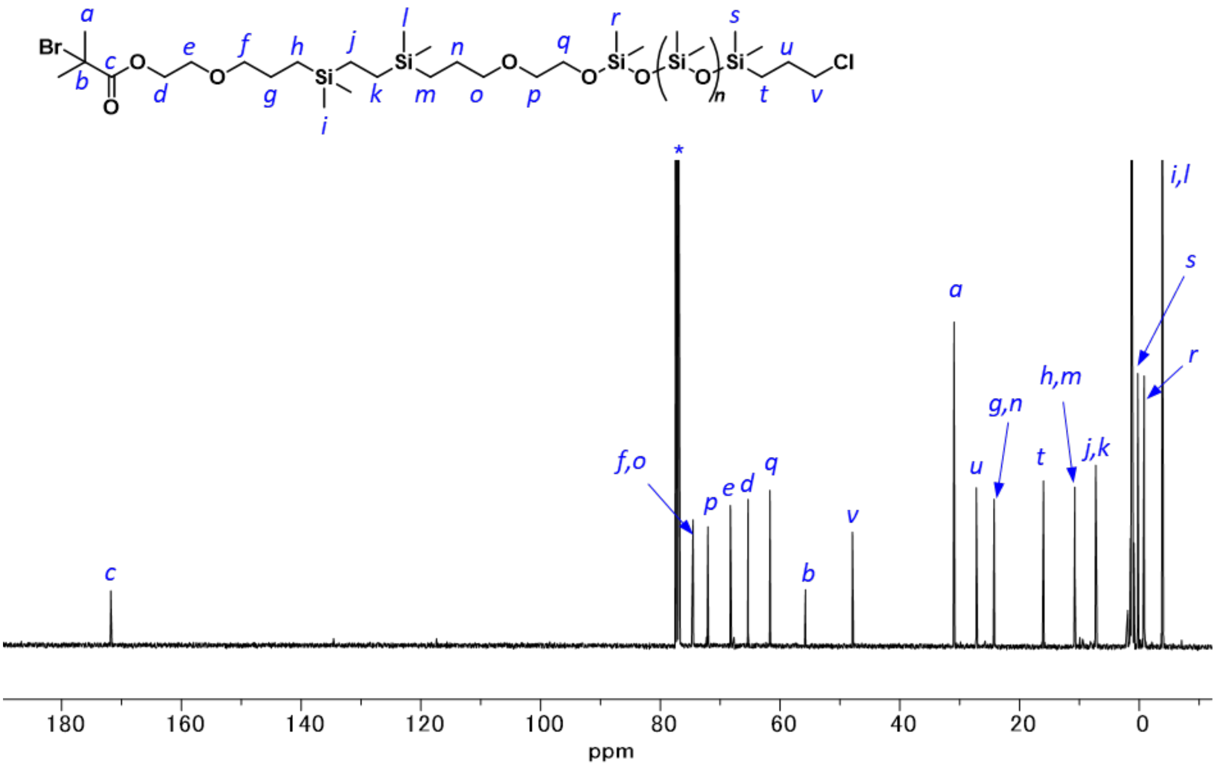

Figure S54. ^{13}C-NMR of ATRP–PDMS–Cl in deuterated chloroform.

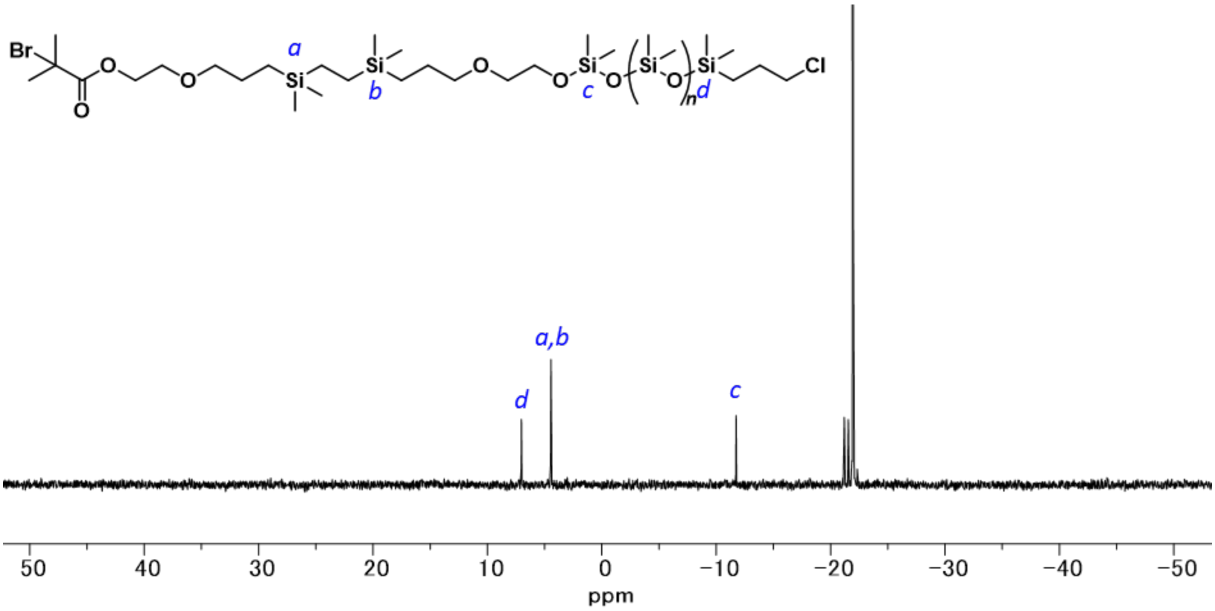

Figure S55. ^{29}Si-NMR of ATRP–PDMS–Cl in deuterated chloroform.

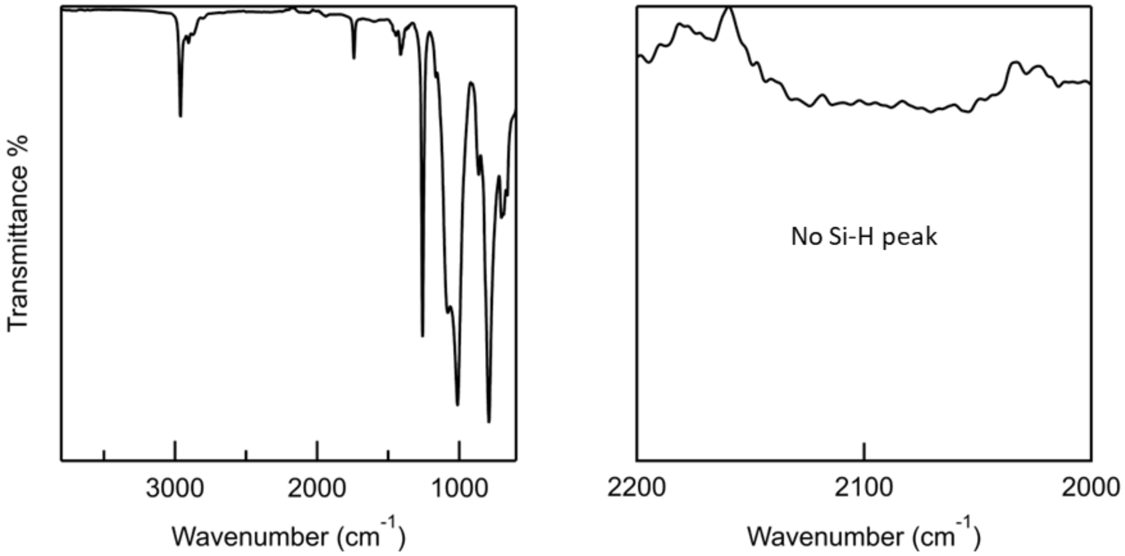

Figure S56. FT-IR of ATRP–PDMS–Cl. Si–H stretching peak was not observed after hydrosilylation.

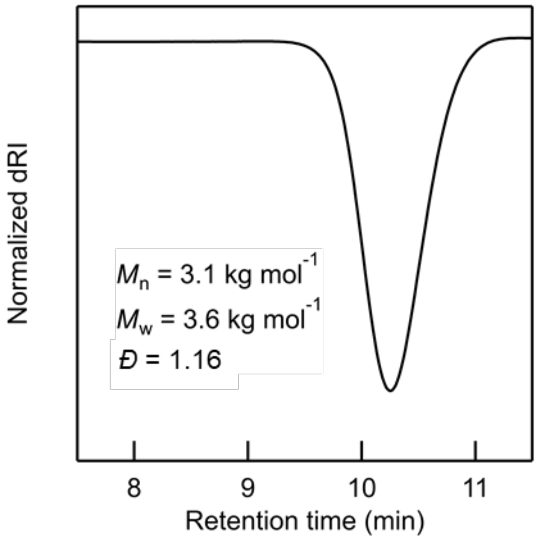

Figure S57. SEC of ATRP–PDMS–Cl.

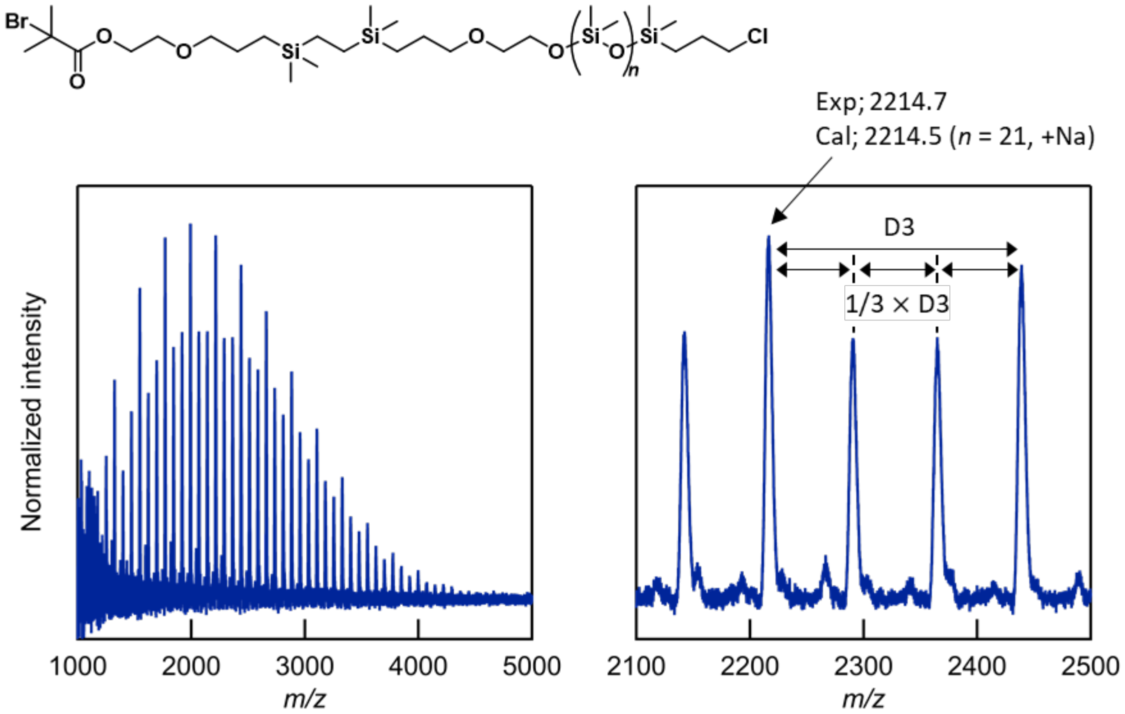

Figure S58. MALDI of ATRP–PDMS–Cl.

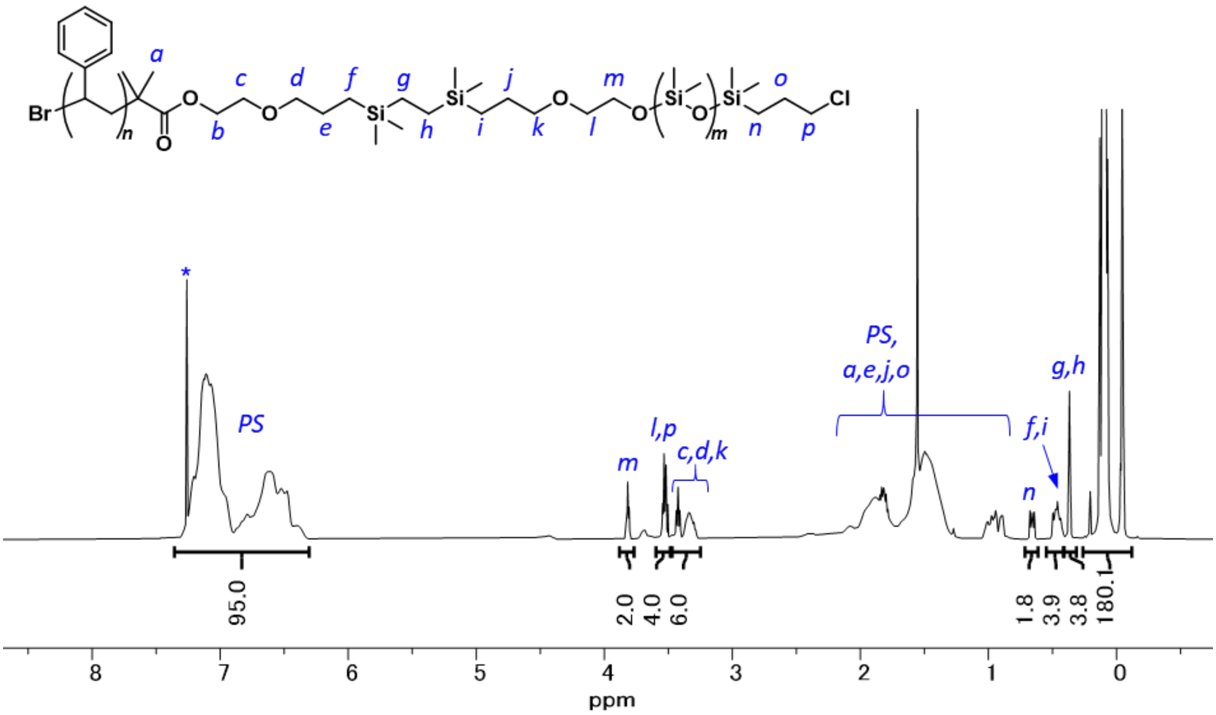

Figure S59. ^{1}H-NMR of PS–*b*–PDMS–Cl in deuterated chloroform.

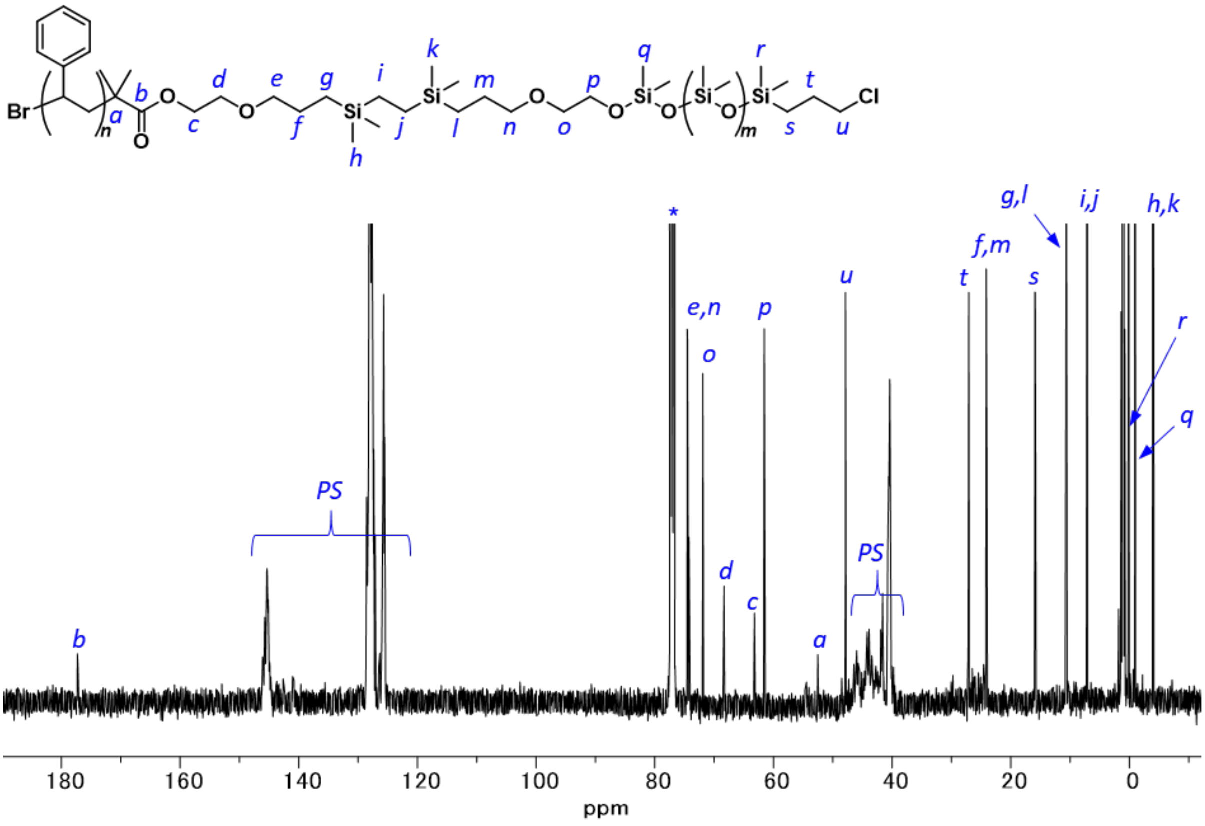

Figure S60. ^{13}C-NMR of PS–*b*–PDMS–Cl in deuterated chloroform.

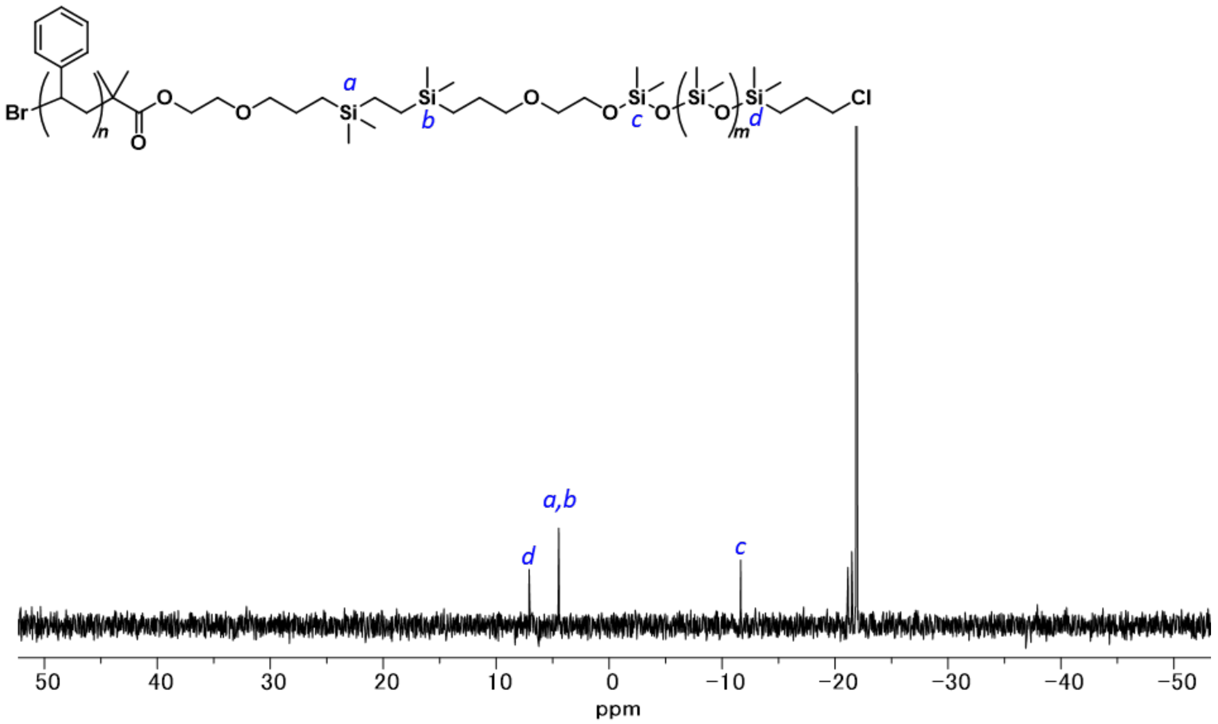

Figure S61. ^{29}Si-NMR of PS–*b*–PDMS–Cl in deuterated chloroform.

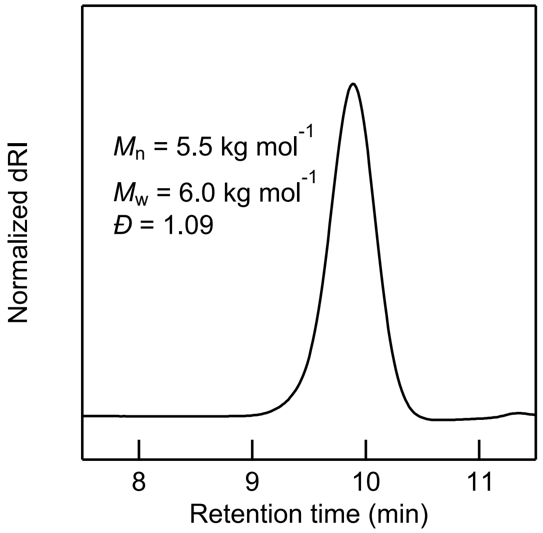

Figure S62. SEC of PS–*b*–PDMS–Cl.

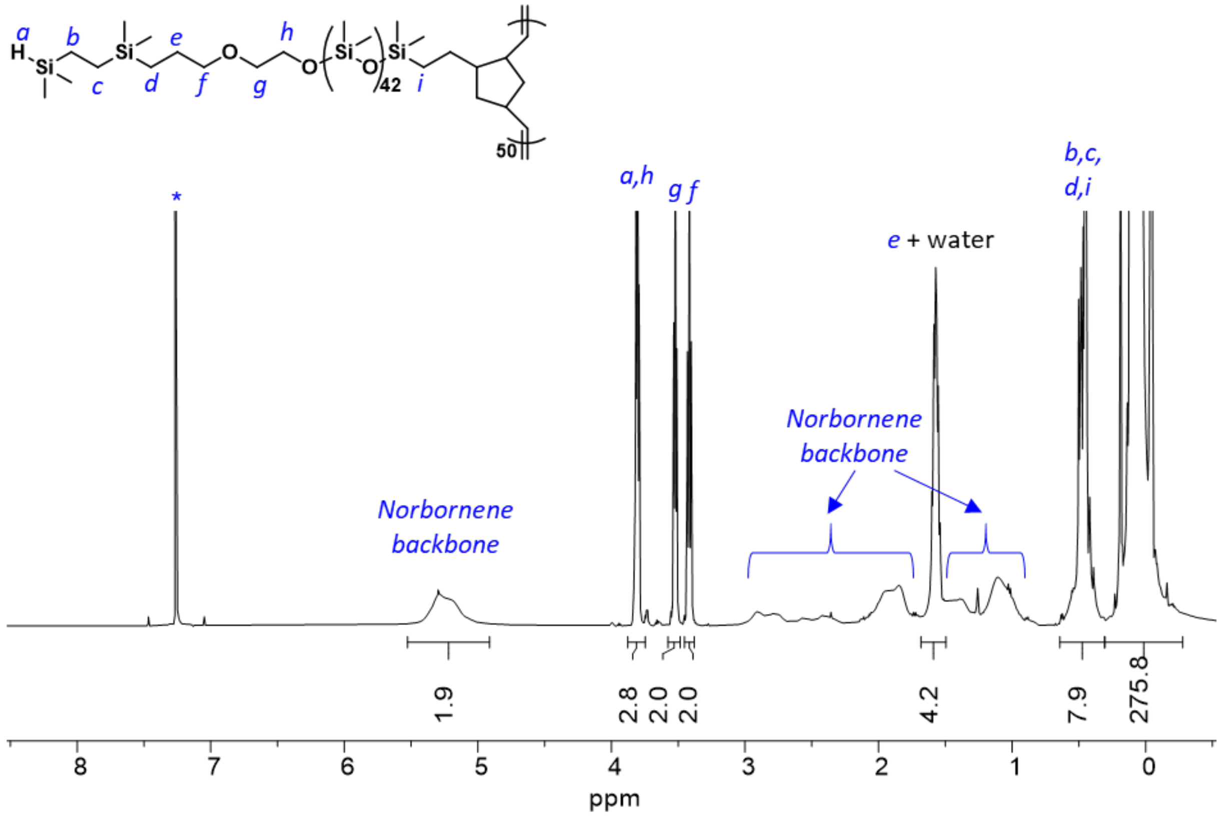

Figure S63. ^{1}H-NMR of PDMS bottlebrush polymer synthesized by ROMP of H–PDMS–Nb in deuterated chloroform.

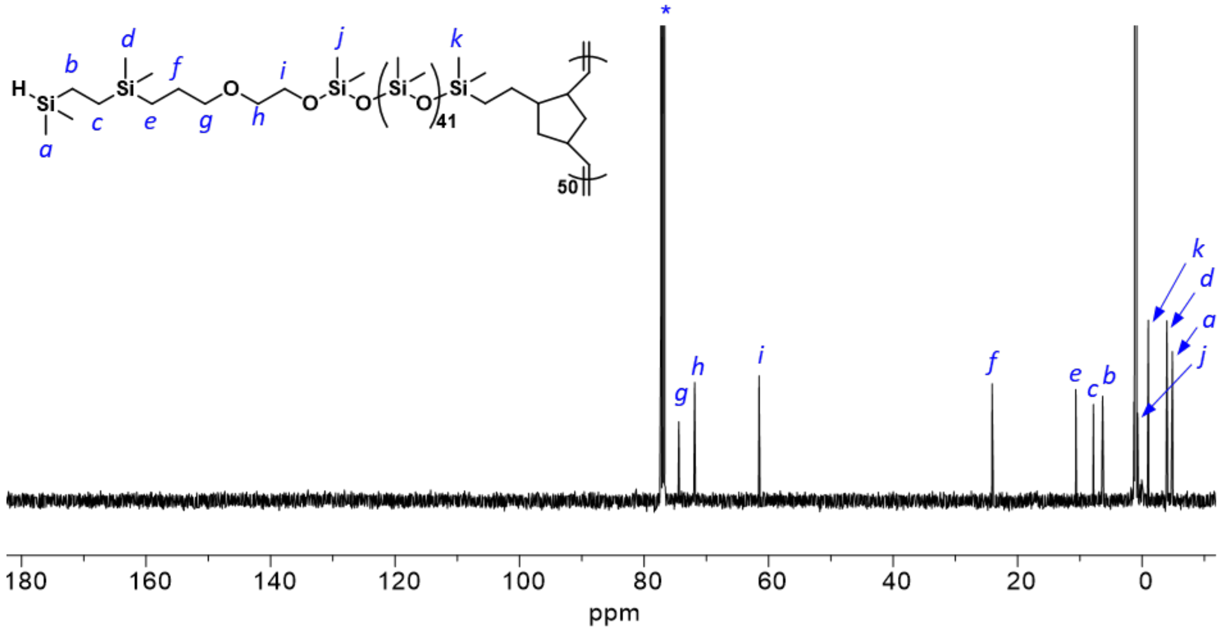

Figure S64. ^{13}C-NMR of PDMS bottlebrush polymer synthesized by ROMP of H–PDMS–Nb in deuterated chloroform.

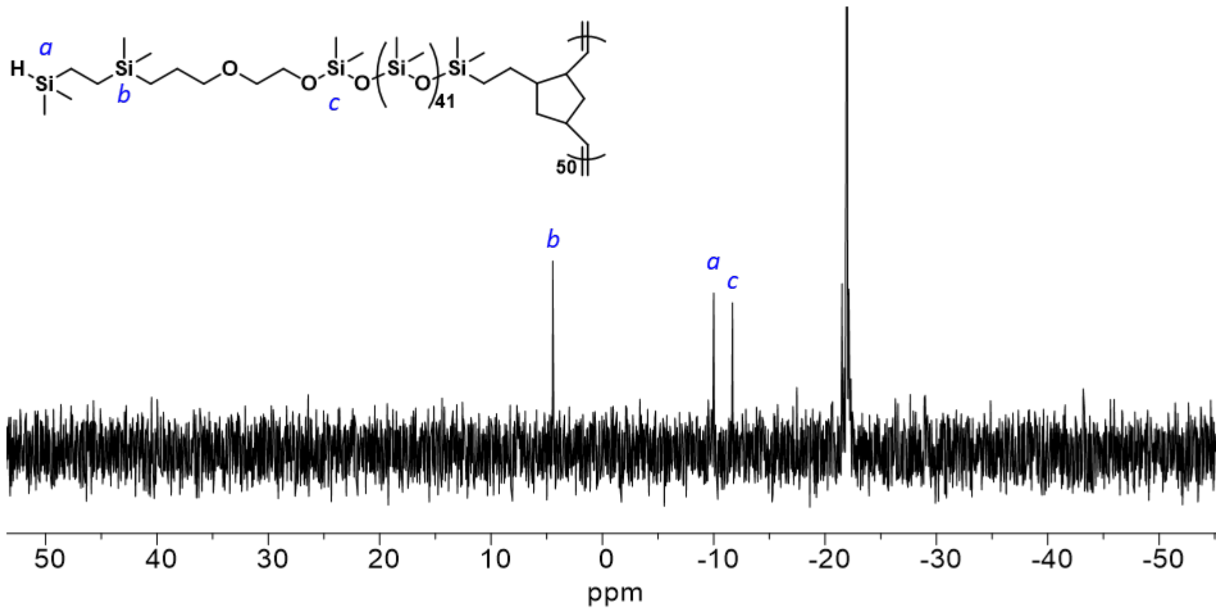

Figure S65. ^{29}Si-NMR of PDMS bottlebrush polymer synthesized by ROMP of H–PDMS–Nb in deuterated chloroform.

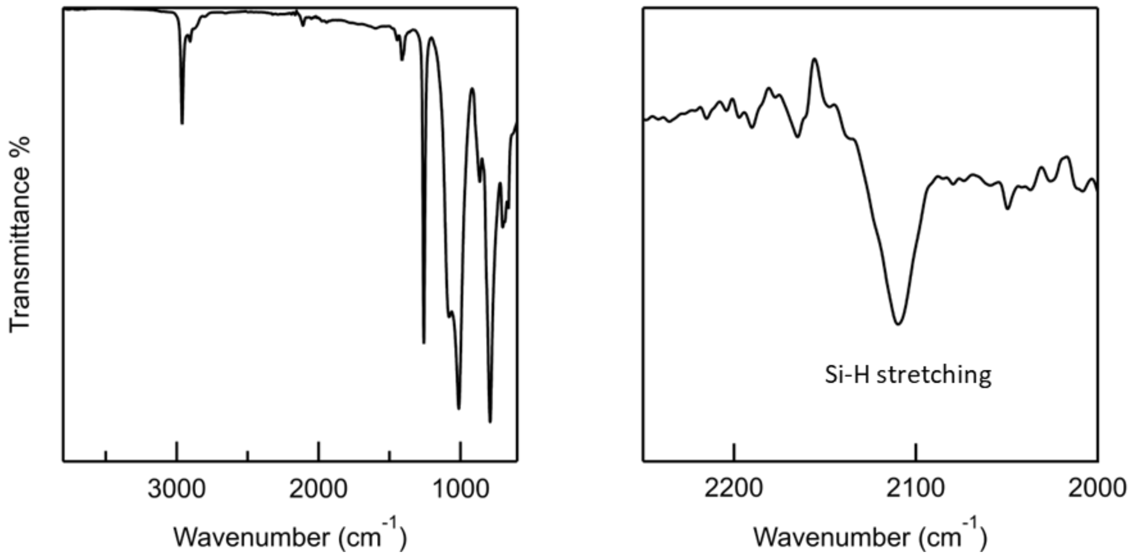

Figure S66. FT-IR of PDMS bottlebrush polymer synthesized by ROMP of H–PDMS–Nb. Si–H stretching peak was observed around 2100 cm^{-1}.

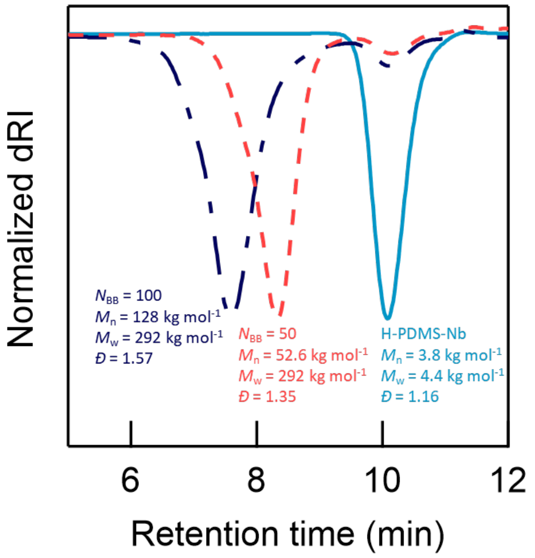

Figure S67. SEC of PDMS bottlebrush polymer synthesized by ROMP on H–PDMS–Nb with targeting N_{BB} of 50 and 100 respectively. The macromonomer (H–PDSM–Nb) peak disappeared and single peak was observed in high molecular weight region after ROMP indicating successful ROMP.

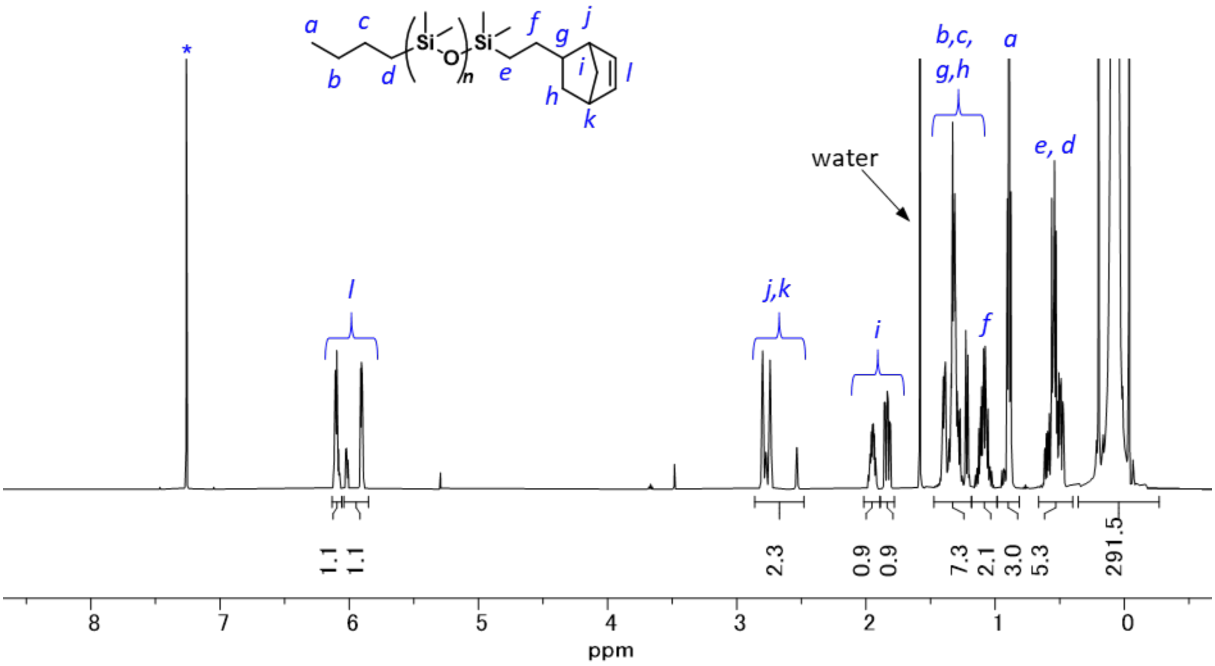

Figure S68. ^{1}H-NMR of Bu–PDMS–Nb in deuterated chloroform.

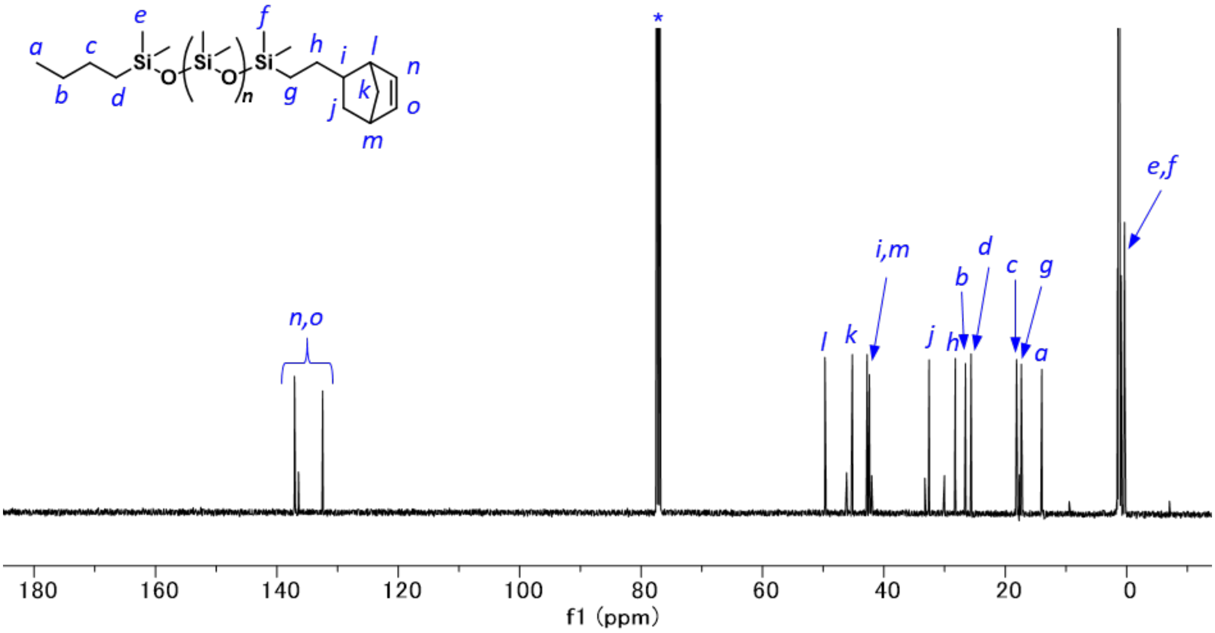

Figure S69. ^{13}C-NMR of Bu–PDMS–Nb in deuterated chloroform.

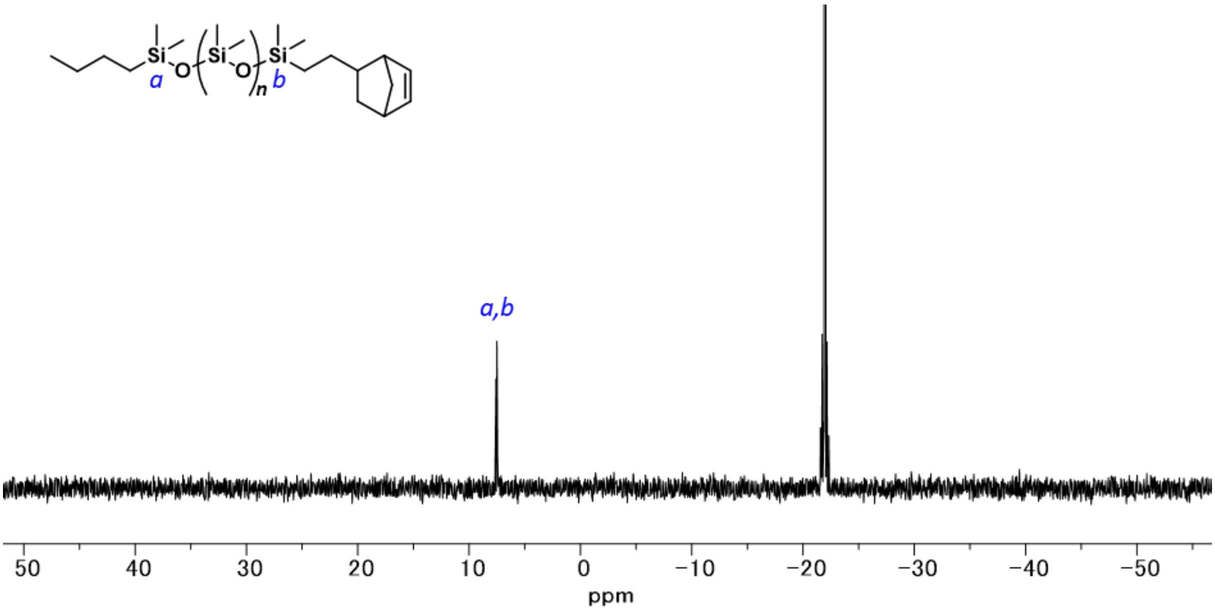

Figure S70. ^{29}Si-NMR of Bu–PDMS–Nb in deuterated chloroform.

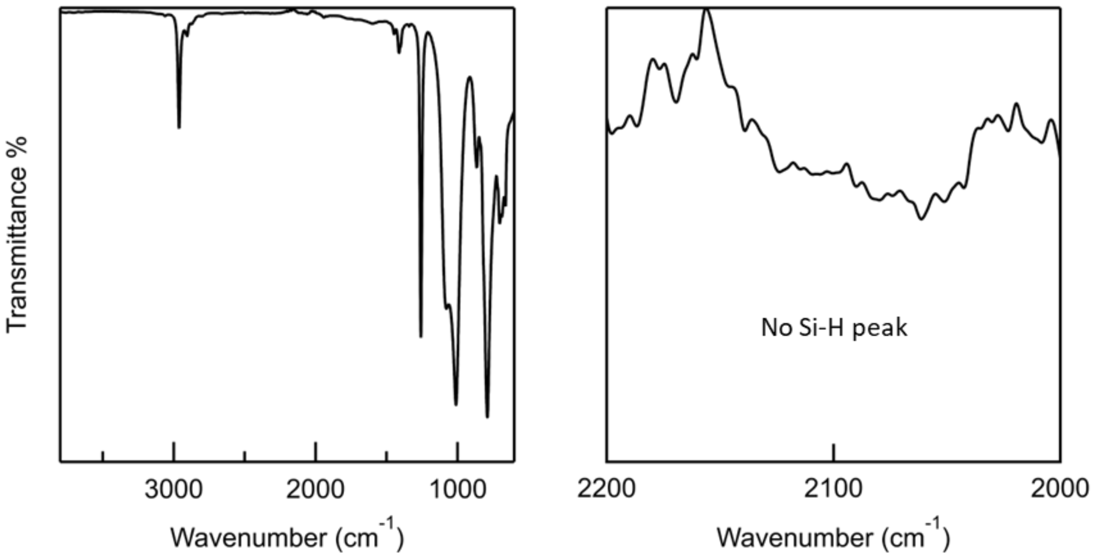

Figure S71. FT-IR of Bu–PDMS–Nb. Si–H stretching peak was not observed.

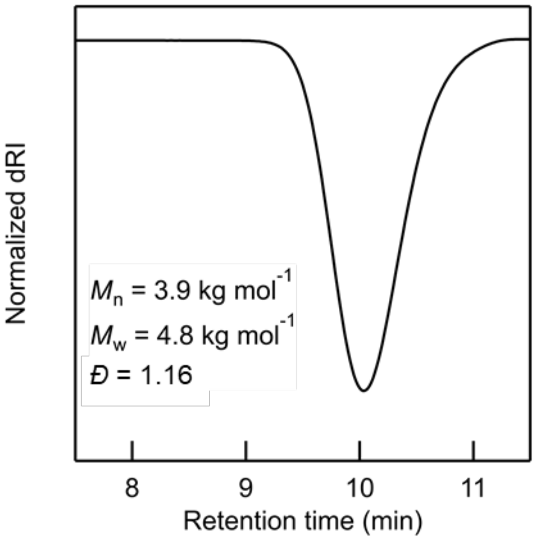

Figure S72. SEC of Bu–PDMS–Nb.

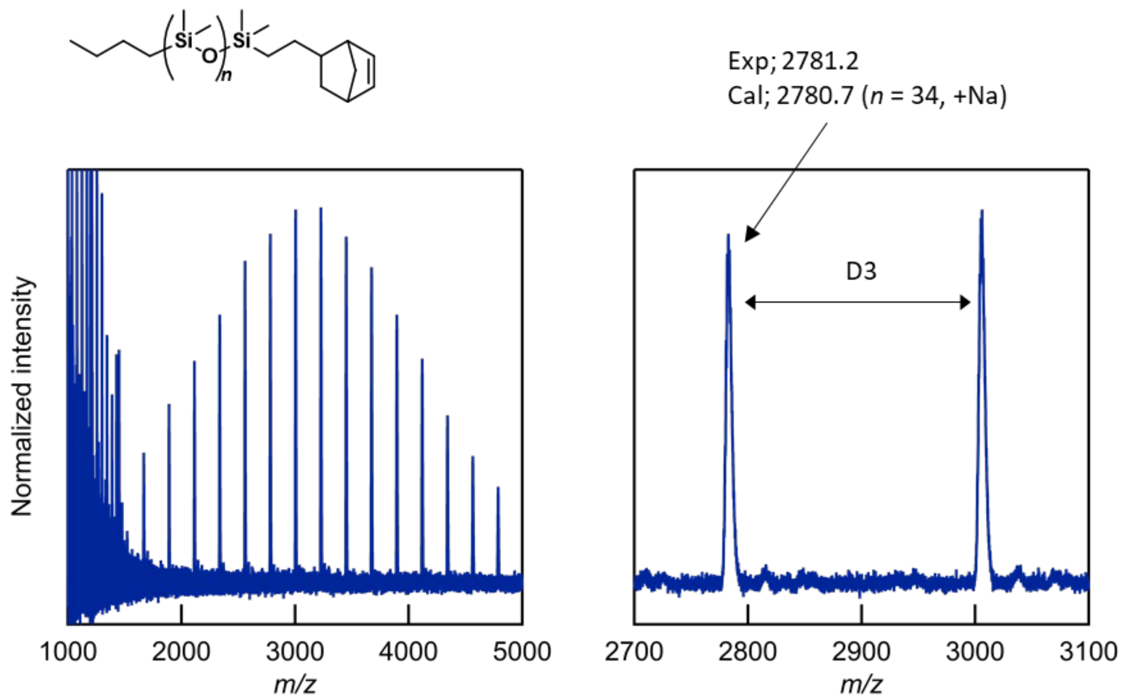

Figure S73. MALDI of Bu–PDMS–Nb.

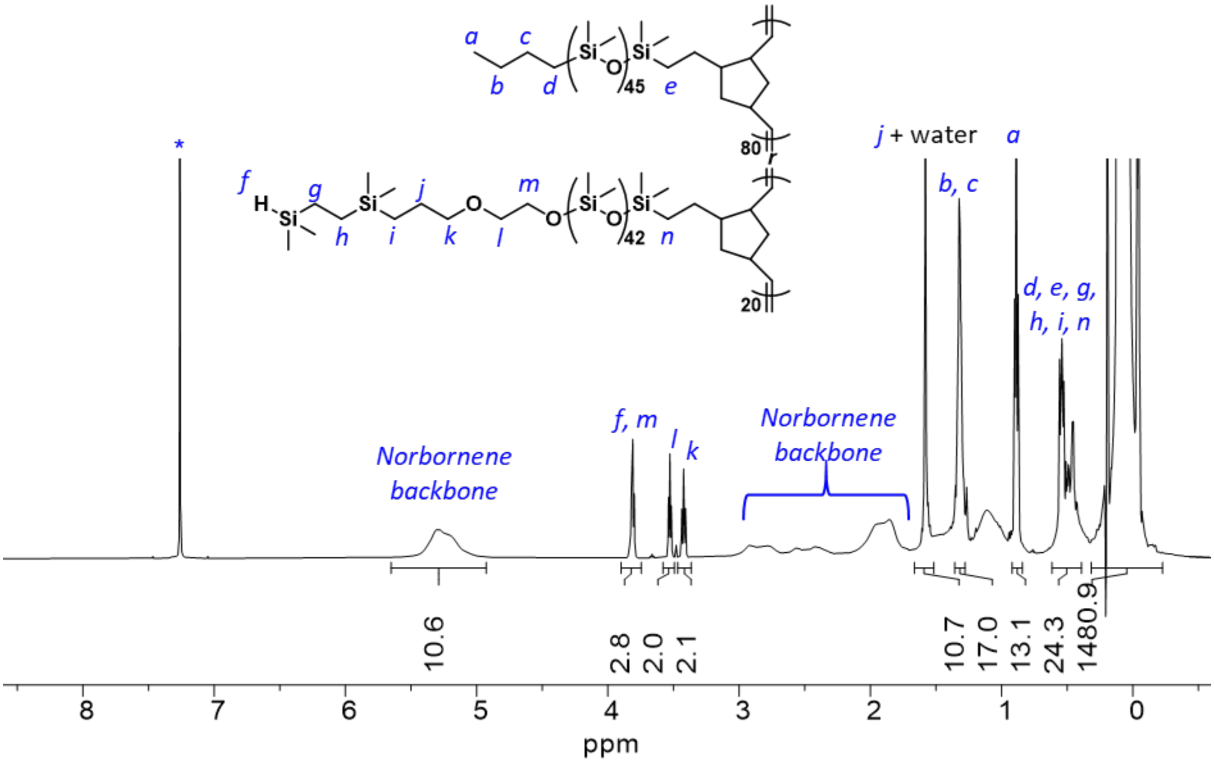

Figure S74. ^{1}H-NMR of PDMS bottlebrush polymer synthesized by ROMP of Bu–PDMS–Nb and H–PDMS–Nb in deuterated chloroform.

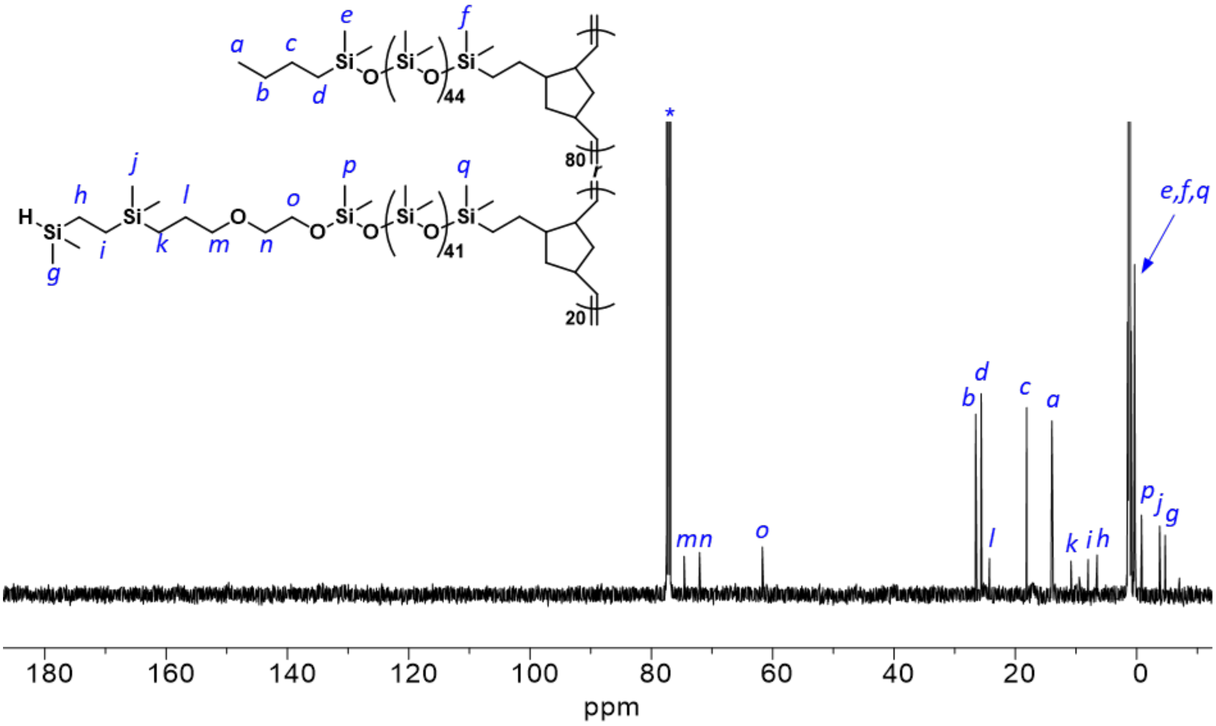

Figure S75. ^{13}C-NMR of PDMS bottlebrush polymer synthesized by ROMP of Bu–PDMS–Nb and H–PDMS–Nb in deuterated chloroform.

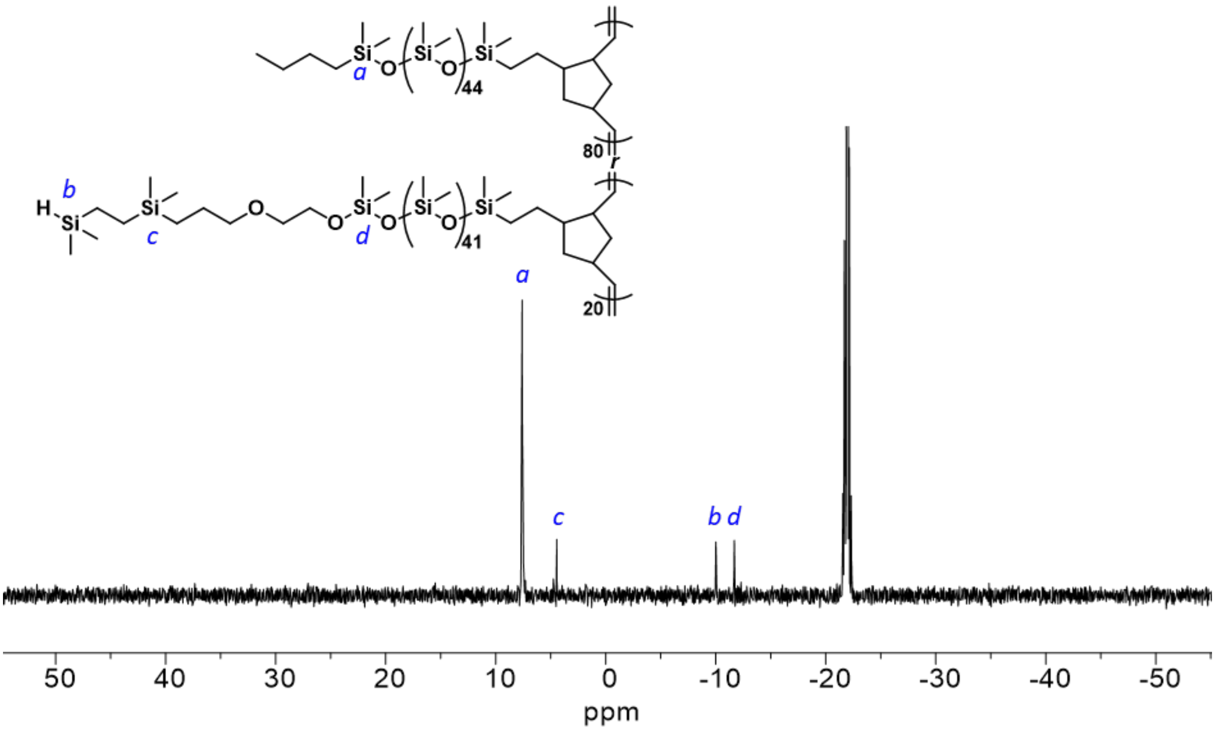

Figure S76. ^{29}Si-NMR of PDMS bottlebrush polymer synthesized by ROMP of Bu–PDMS–Nb and H–PDMS–Nb in deuterated chloroform.

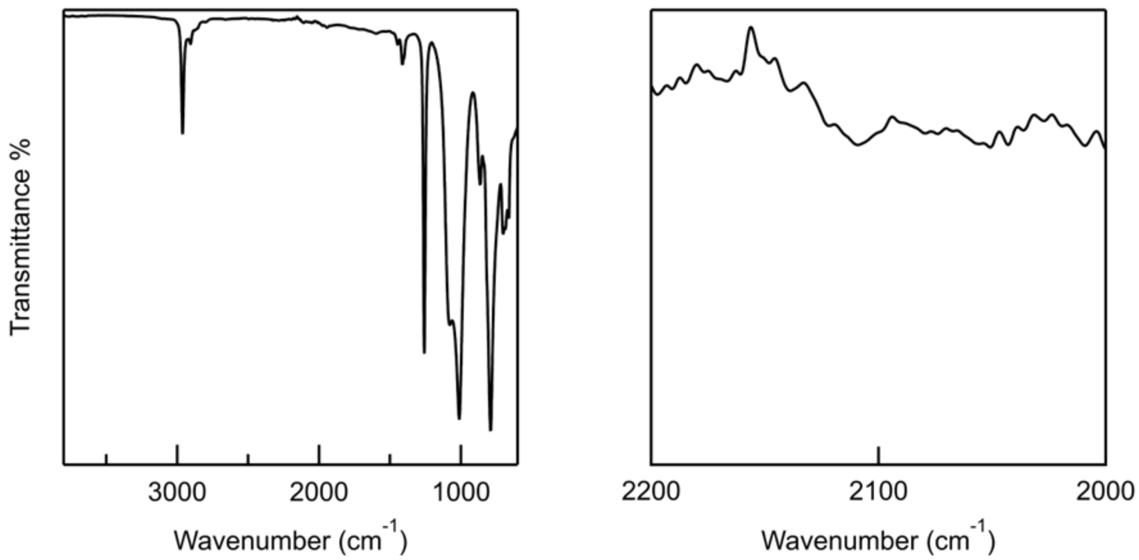

Figure S77. FT-IR of PDMS bottlebrush polymer synthesized by ROMP of Bu–PDMS–Nb and H–PDMS–Nb. Si–H stretching peak was observed around 2100 cm^{-1}.

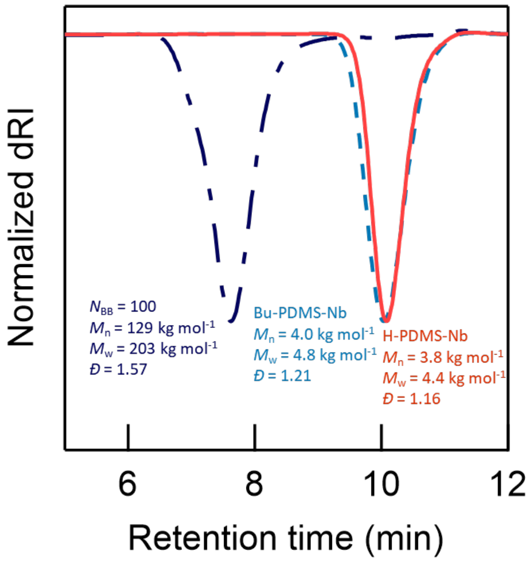

Figure S78. SEC of PDMS bottlebrush polymer synthesized by ROMP of Bu–PDMS–Nb and H–PDMS–Nb. The macromonomers (Bu–PDMS–Nb and H–PDMS–Nb) peaks disappeared and single peak was observed in high molecular weight region after ROMP, indicating successful ROMP.

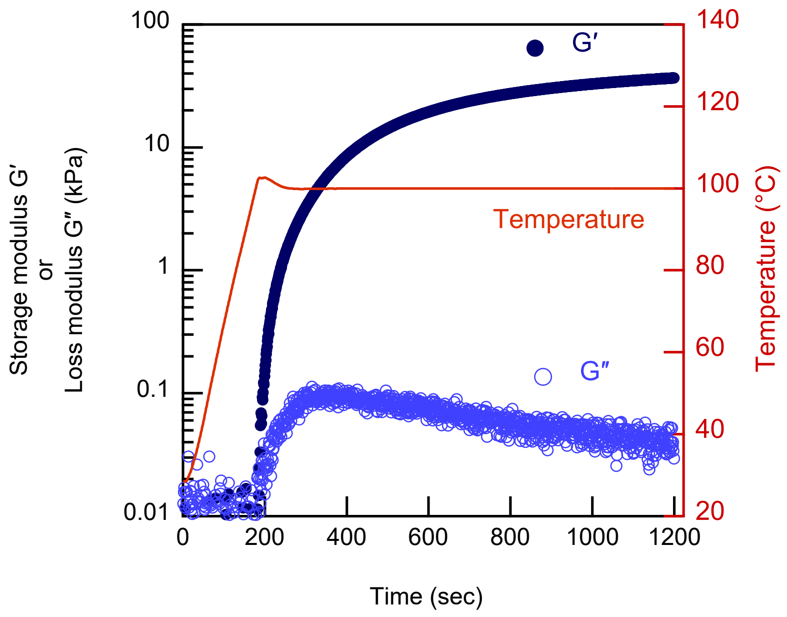

Figure S79. Curing profile of PDMS bottlebrush network formation. Storage modulus dramatically increased once the temperature of rheometer reached 100 °C, then reached plateau within 30 minutes.

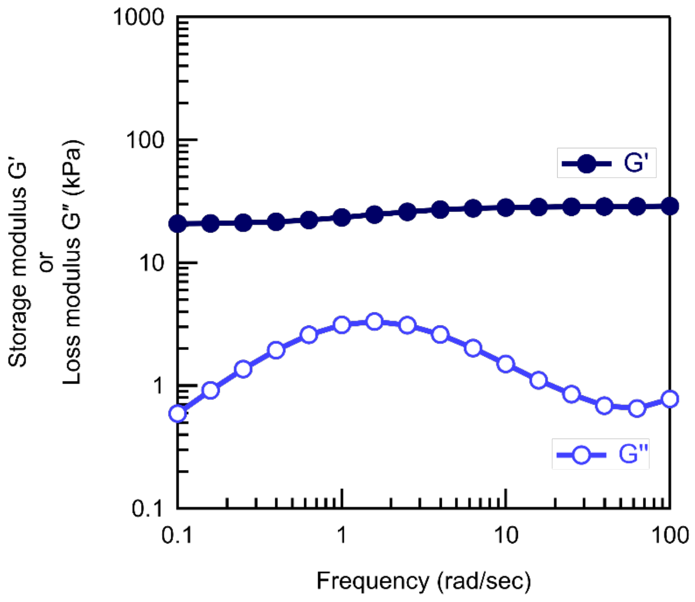

Figure S80. Frequency sweep of PDMS bottlebrush network. Storage modulus was steady and order higher than loss modulus in wide range of frequency indicating the network was solidified by crosslinking. The Shear modulus was about 30 kPa which is super soft as non-solvent network attributed by bottlebrush structure.

References

(1) Lamers, B. A. G.; de Waal, B. F. M.; Meijer, E. W. The Iterative Synthesis of Discrete Dimethylsiloxane Oligomers: A Practical Guide. *J. Polym. Sci.* **2021**, *59* (12), 1142–1150.

(2) Gharakhanian, E. G.; Deming, T. J. Versatile Synthesis of Stable, Functional Polypeptides via Reaction with Epoxides. *Biomacromolecules* **2015**, *16* (6), 1802–1806.

(3) Love, J. A.; Morgan, J. P.; Trnka, T. M.; Grubbs, R. H.; Bissell, N. A.; Boden, R. J.; Bushby, C. W. G.; Fishwick, E.; Holland, B.; Movaghar, A Practical and Highly Active Ruthenium-Based Catalyst That Effects the Cross Metathesis of Acrylonitrile. *Angew. Chem. Int. Ed.* **2002**, 41 (*21*), 4035–4037.
